# Incorporating adaptation and resilience into an integrated watershed and coral reef management plan

**DOI:** 10.1371/journal.pone.0253343

**Published:** 2021-06-24

**Authors:** David A. Gibbs, Jordan M. West, Patricia Bradley

**Affiliations:** 1 Oak Ridge Institute for Science Education fellow at U.S. Environmental Protection Agency, Washington, D.C., United States of America; 2 Office of Research and Development, U.S. Environmental Protection Agency, Washington, D.C., United States of America; 3 Patricia Bradley, Tetra Tech, Inc., Owings Mills, MD, United States of America; Biodiversity Research Center, TAIWAN

## Abstract

Changing environmental conditions are forcing natural resource managers and communities to adapt their strategies to account for global shifts in precipitation, temperature, sea level and more, all of which are occurring in addition to local human impacts. Adapting to threats from climate change requires a fundamental shift in the practice of natural resource management through the development of forward-looking “climate-smart” goals and strategies. Here we present a proof-of-concept application of a decision-support tool to help design climate-smart management actions for the watershed and coral reef management plan for Guánica Bay watershed in southwest Puerto Rico. We also explore the connection between adaptation planning and coral reef resilience, using a recently developed Puerto Rico-wide reef resilience assessment. In the first phase of the study, we used the publicly available Adaptation Design Tool to draft initial climate-smart versions of twelve proposed management actions. In the second phase, two actions (dirt road management on steep slopes, and coral reef restoration) were further refined through consultations with local experts to make more detailed design adjustments; this included the option to use information from the coral reef resilience assessment to inform design improvements. The first phase resulted in moderately detailed assessments that broadly accounted for anticipated direct and indirect effects of climate change on the planned management actions. The second phase resulted in more site-specific technical assessments and additional important design details. The expert panel charged with discussing climate-smart reef restoration around Guánica used the reef resilience assessment to guide discussion of reef restoration, highlighting the importance of having such information available for adaptation planning. This study demonstrates how climate change impacts can be effectively incorporated into a management plan at the most granular level of planning and how a structured, formalized process can be as valuable as the resulting adaptation information.

## Introduction

Natural resource managers and local communities need to make both near-term (years) and long-term (decades) decisions about how to manage environmental challenges. The effectiveness of these decisions increasingly depends on successful adaptation to climate change, which can affect management targets directly and through complex interactions with non-climate stressors [1–4]. Climate change adaptation has been receiving increasing attention in response to societal concerns regarding adverse impacts to species, ecosystems and human well-being. Much of the adaptation guidance developed so far has been generic and aimed at high-level policy rather than on formulating specific actions that are “climate-smart”. Structured approaches to adaptation planning that integrate existing methods for vulnerability assessment with design and evaluation of effective adaptation responses are needed [5,6]. This is because management plans and actions that are well designed for current climatic conditions may not perform as well under future conditions, such that the preferred course of action may differ when climate change is considered. All natural resource management actions should be assessed for their long-term effectiveness and made climate-smart by accounting for potential climate change effects in their design, implementation, and maintenance [5,6]. Practical efforts to operationalize climate-smart adaptation require consideration of climate change impacts throughout the natural resource management planning and implementation process. A key component is collaboration between communities, scientists, and managers in design of the climate-smart management actions.

**Fig 1:**
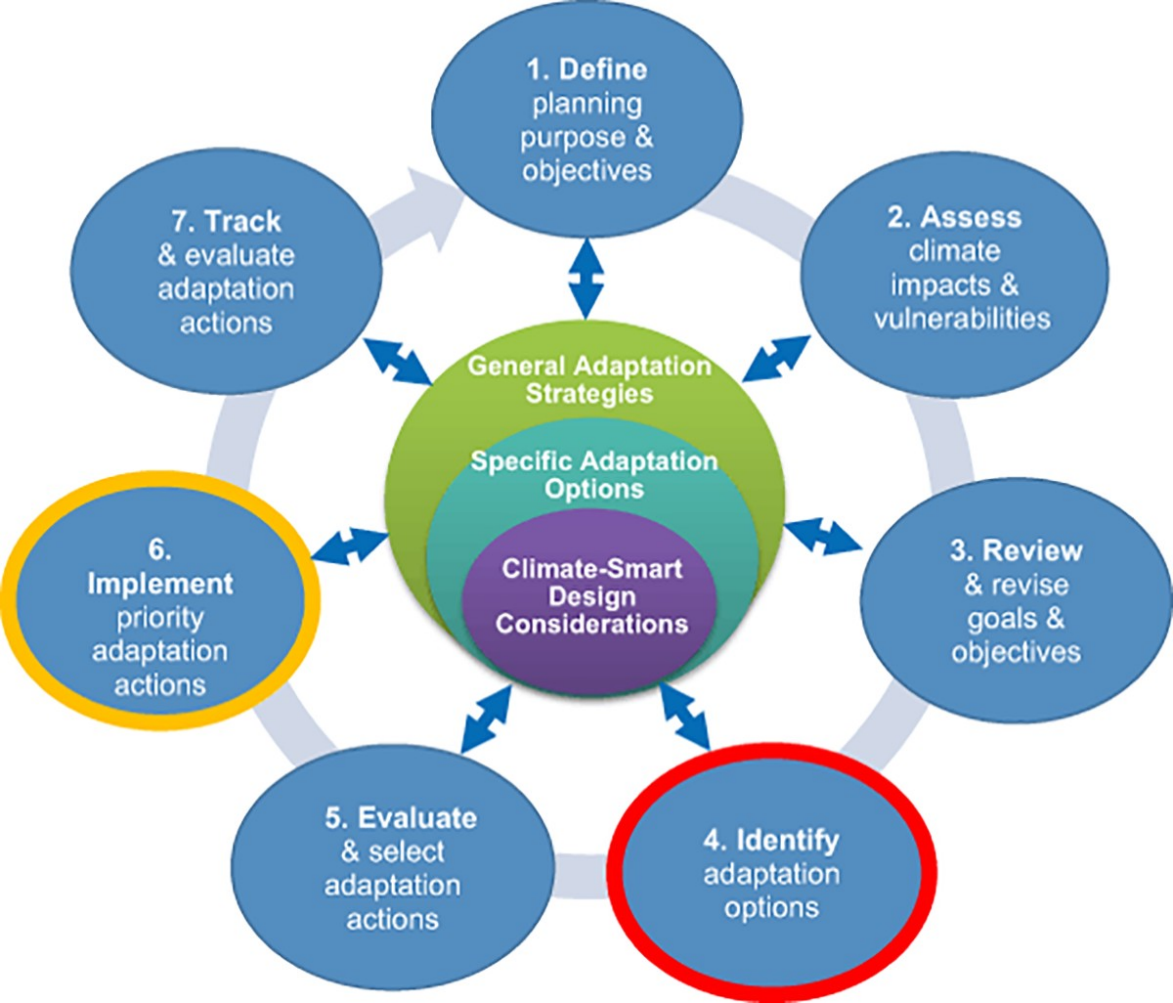
Climate-smart planning cycle with adaptation design framework. The Adaptation Design Tool [15] supports Steps 4 and 6 of the cycle, outlined in red and yellow, respectively.

The climate-smart planning cycle (Fig 1) [6] assists natural resource managers with systematically addressing the challenges associated with adapting to climate change (e.g., potentially large uncertainties around specific effects in a given location, the timing of climate change effects, and indirect effects) during each step of the planning process. Tools, guidance and numerous case studies exist for parts of the climate-smart management cycle, such as Step 2 vulnerability assessments [7–9] and resilience assessments [10–14]. However, there is less guidance on identifying and designing adaptation options for consideration (Step 4), or how to engage in more detailed climate-smart design of selected actions with experts in preparation for implementation (Step 6) [6]. These two steps are the focus of this study.

One available resource for identifying and designing climate-smart adaption options in Steps 4 and 6 is the Adaptation Design Tool (ADT) [15]. The ADT lays out a stepwise process structured through worksheets by which natural resource decision makers can use information collected from local vulnerability and resilience assessments to link management actions to climate-smart adaptations. Through a series of targeted questions about the effects of climate change on stressors being managed and system responses, the ADT can help scientists, natural resource managers, and other stakeholders work together to apply the best available science to brainstorm and design effective actions. The objective is to empower managers to evaluate and select priority actions (Step 5, Fig 1) based on best-available information that proactively takes climate change into consideration, while also identifying and documenting information gaps, uncertainties, interactions among actions, and the sequences in which management actions should be executed [16].

**Fig 2:**
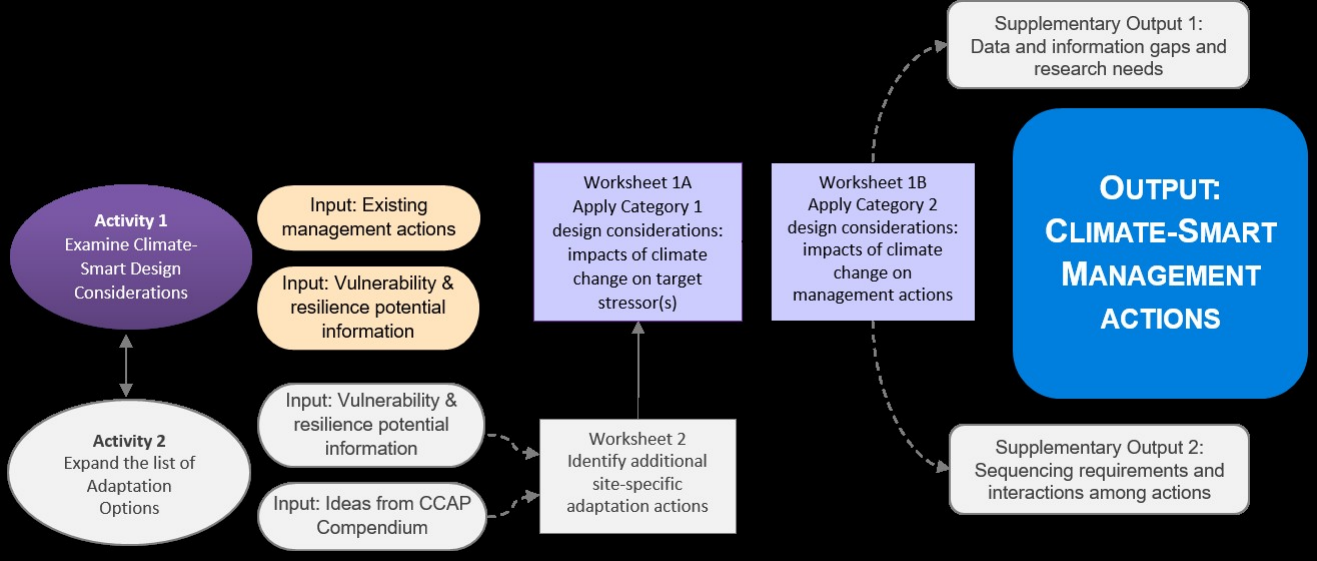
Flow chart of the Adaptation Design Tool [15]. Outputs from worksheets 1A and 1B from this case study can be found in S3 Table. Activity 2 and supplementary outputs have been faded in the figure, as they were not part of this study. The CCAP (Corals and Climate Adaptation Planning) Compendium, a thematically organized compilation of adaptation options for coral reefs, can be found in [6].

In the ADT (Fig 2), Activity 1 helps managers and planners assess the effects of climate change on proposed management actions and adjust their designs to be more robust under changing conditions. Users list each management action under consideration and respond to questions about two categories of design considerations: 1) how climate change may affect stressors that are being managed by, or could affect, the action (worksheet 1A); and 2) what these indirect effects, along with any direct destructive effects of climate change on structural components, may mean for the effectiveness of the management action and how it could be modified to remain effective (worksheet 1B). Activity 2 (worksheet 2) then aids the brainstorming of new actions based on climate impacts and vulnerabilities that have not yet been addressed. The new actions are in turn passed through Activity 1 for analysis and design. The ultimate outputs of this structured process are descriptions of climate-smart management actions that can be evaluated (in Step 5 of the climate-smart planning cycle) instead of the actions that do not explicitly incorporate climate change, thus ensuring that climate change effects are considered in management decisions. While the ADT does not include analysis of cost-benefit ratios, political or social limitations, or other non-climate factors, insights on these issues are documented in the area for notes in Activity 1 and then considered during Step 5 evaluation and selection of priority actions. Once priority actions have been selected, the ADT can be used again with subject matter experts to engage in more detailed implementation planning and design.

Although the ADT was developed and tested with the help of coral reef management practitioners using example actions in specific locations [6,15], its implementation in an actual management planning context has not been formally explored. In this case study, we concentrated on Activity 1 of the tool, examining existing brainstormed actions in a concrete management situation. This proof-of-concept exercise aims to increase the tool’s utility by revealing strengths and identifying areas for improvement during real-world implementation. It also demonstrates how climate change can be integrated into environmental decision making, rather than handled as a separate process. If climate change was not incorporated into the original management plan, a good time to incorporate climate change is during management plan revision because, during this time, activities are being reevaluated, reprioritized, and redesigned. As priority actions are being considered for an updated plan, designing actions to be climate-smart from the beginning may be easier and more economical than retrofitting adaptation into them later.

This study focused on a management plan revision for the Guánica Bay watershed and associated coastal habitats in southwest Puerto Rico, with special focus on coral reef ecosystems. The watershed and coastal area are relatively well studied with active management activities. The current watershed management plan addresses a variety of threats (see Site Description section in Methods), many of which are expected to be affected by climate change. Thus, this was a good opportunity to incorporate climate change considerations in a planning cycle using the ADT, covering both watershed and coral reef management activities. As part of this process, we simultaneously explored the connection between adaptation planning and coral reef resilience, using a recently developed Puerto Rico-wide climate change reef resilience assessment [17] that we modified to focus on southwest Puerto Rico. Reef resilience assessments use reef attributes that are indicators of sensitivity and adaptive capacity to estimate the relative resilience of sites and present spatial information on surveyed reef locations [13]. Like vulnerability assessments, resilience assessments inform management decisions, programs, and activities, and are thus part of the climate-smart cycle and essential inputs to the ADT. However, this is the first case where resilience assessment information and this tool have been applied together in a structured process. In this paper, we describe how the ADT and resilience assessment were used in an actual watershed management planning cycle, report on resulting improvements to adaptation designs, and discuss lessons learned from the case study.

## Methods

### Site description

**Fig 3:**
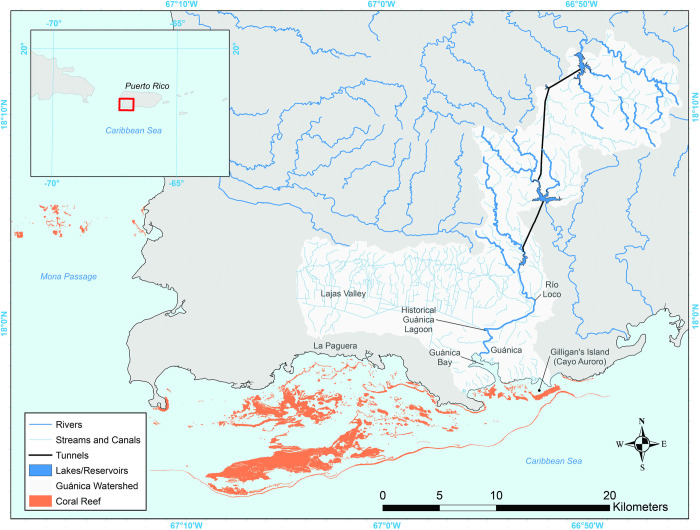
Map of the Guánica Bay watershed and southwest Puerto Rico reef. Rivers and streams from [25] and coral extent from [26].

The Guánica Bay watershed (390 km^2^), located approximately 32 kilometers west of Ponce and 160 kilometers southwest of San Juan, Puerto Rico, is dominated by evergreen forest (51% of land cover) and grassland (26%), with the remaining watershed consisting of agricultural land (e.g., cultivated land, pasture/hay, coffee), scrub/shrub habitat, developed land, wetland, and bare land. Guánica Bay itself receives fresh water primarily from the mouth of the Rio Loco at the town of Guánica near the northern end of the bay. In the 1950s, five reservoirs and two hydroelectric plants were built on the mountain ridges north of Guánica Bay to increase and regulate potable water from the high elevation watersheds of the central mountain range for use by the populations of coastal cities [18–20]. Canals and streams were also constructed to divert water from just below the southernmost reservoir along the foothills to the west to provide agricultural irrigation in the Lajas Valley. A long drainage channel was constructed along the southern edge of the Lajas Valley to return the water eastward into the Rio Loco near its mouth [21–24] (Fig 3).

The Guánica Bay Watershed includes several existing conservation areas, including the Guánica Dry Forest (which is both a state forest and a United Nations International Biosphere Reserve), the Punta Ballenas Reserve (which is along the coast of the Guánica Forest and is managed as part of the Guánica Dry Forest), *Cayo Aurora (*commonly known as Gilligan’s Island also managed as part of the Guánica State Forest), and the Susúa State Forest (located between Yauco and Sabana Grande in the foothills of the Central Range). There is also the Lajas Valley Agricultural Reserve, which is managed for agricultural use. The Guánica Lagoon is also currently managed for agricultural use but has been identified by the Puerto Rico Department of Natural and Environmental Resources as a priority for wetland restoration.

Prior to the 20^th^ century, the coral reefs of Puerto Rico and the wider Caribbean were dominated by branching corals, with structure and diversity enhanced by several species of mounding corals as well as sea urchins, large schools of game fish, and abundant sharks, turtles, and marine mammals [27]. Coral reefs still form extensive structures at the mouths of coastal embayments such as Guánica Bay and fringe many small islands along Puerto Rico’s southern coast [28–30]. However, these coral reefs have been significantly degraded over the past four decades by the cumulative effects of global and local stressors [2,30,31]. Local stressors (e.g., sediment, nutrient, and contaminant efflux from human activities in the adjacent watershed) have affected the reefs near Guánica and La Parguera (to the west), with dramatic reductions in living colonies of reef-building stony corals [32–34]. These losses have been further exacerbated by the impacts of rapid environmental changes and extreme events, such as hurricanes, altered precipitation patterns, droughts, ocean acidification and warm-water bleaching events [29,35].

In 2005, the U.S. National Oceanic and Atmospheric Administration’s (NOAA) Coral Reef Conservation Program contracted the Center for Watershed Protection (CWP) to develop a watershed management plan for the Guánica Bay watershed [21]. The plan identified the principle sources of pollution that threatened coral reef habitats in southwest Puerto Rico: upland erosion in coffee farms, reservoir sedimentation and transport, in-stream channel erosion, loss of the Guánica Lagoon, legacy contaminants, and inadequate sewage treatment. A series of management actions were identified to target these sources of pollution. In 2018, a follow-on report [36] summarized the activities that had taken place since the original management plan was released and provided a list of recommended actions for the future protection and restoration of the watershed and coral reefs. A revised management plan for the Guánica Bay watershed is currently being developed (R. Viquiera Rios, personal communication).

The U.S. Coral Reef Task Force (USCRTF) has identified terrigenous sediment as a major stressor to coral reefs at large and determined that its reduction is essential for maintaining coral reef resilience [37,38]. In southwest Puerto Rico, mass forest clearing, poor soils, and runoff associated with dirt roads, particularly in the mountain ridges, has resulted in increased sediment deposition and transport in the Guánica Bay watershed [39]. At the scale of individual farms, dirt roads have been found to be responsible for more than 90% of the annually generated sediment [40]. For this reason, management of sedimentation from dirt roads (e.g., water conveyance, diversion and flow reduction) is a priority and was examined in greater detail by an expert panel using the ADT (see Application of the Adaptation Design Tool section below).

NOAA’s National Marine Fishery Service (NMFS) released a recovery plan for elkhorn (*Acropora palmata*) and staghorn coral (*A*. *cervicornis*) in 2015 that included several adaptation measures, including increasing monitoring of disease and bleaching events, reducing local impacts of temperature stress (e.g., shading of reefs, pumping cooler waters onto reefs), researching the viability of land-based rearing and wild re-stocking of coral species, and testing approaches to culture resistant and/or resilient strains of corals (e.g., disease or biotoxin resistance, thermal or pH tolerance) [24]. For this reason, coral restoration, including land-based rearing (coral nursery) and wild re-stocking of species (outplanting), were examined in greater detail by an expert panel using the ADT (see Application of the Adaptation Design Tool section below).

Puerto Rico has experienced a variety of climate change effects which are expected to continue this century (S1 Table). Air and sea surface temperatures have increased throughout Puerto Rico, with sea surface temperatures warming faster in southern Puerto Rico than in northern Puerto Rico. Sea level has also risen, while precipitation trends are unclear so far.

### Application of the Adaptation Design Tool

Prior to applying the ADT, we compiled a list of potential management actions being considered for the updated Guánica watershed and reef management plan. These actions were drawn from the 2008 watershed management plan [21], summaries of multiple Guánica stakeholder workshops [22,23], and conversations with local managers about their priorities for the new plan (R. Viquiera Rios and P. Sturm, personal communication). This yielded a list of 80 potential actions, from which we selected using the following criteria: (1) adequate information and specificity about the proposed action and (2) at least some potential effect of climate change on the action. Examples of the types of actions that were excluded based on these criteria included developing rainwater collection systems (non-specific) and improved secondary school education about the importance of coral reefs (the education itself is not directly affected by climate change, though the content is). Using these criteria, we pared down the list to 14 actions (Table 1).

**Table 1:** The twelve first phase management actions and two actions (in *italics*) that could not be addressed during the first phase because no reef resilience assessment had been completed.

Action number	Action
1	Plant cover crops in Guánica Valley farms
2	Plant riparian buffers along the Rio Loco where it passes through farms
3	Replace sun-grown coffee with shade-grown coffee
4	Hydroseed bare soils associated with roads and homes
5	Construct swales to treat urban stormwater (as a type of green infrastructure)
6	Restore Guánica Lagoon
7	Use water diversion structures and flow reduction practices (e.g., water bars, vetiver and rock check dams, culverts) to manage sediment from existing dirt hills and mountain roads
8	Construct wetlands for tertiary treatment at the Guánica WWTP
9	Protect seagrass meadows
10	Protect mangrove forests
11	Capture larval fish of target species and establish reef fish aquarium-based nurseries
12	Collect corals and establish aquarium-based coral nurseries
13	*Release nursery-raised fish (from action 11) on reefs*
14	*Outplant nursery-raised corals on reefs (from action 12) around Gilligan’s Island to protect the coastline*

Once the resilience assessment was available, it was possible to address action 14 along with action 12 in the second phase because they were so closely related to each other. Actions 7, 12, and 14 were covered during the second phase. Action 13 was not addressed in either the first or second phases.

Next, we applied the ADT in two phases. The goal of the first phase was to develop basic climate-smart information for more robust evaluation and selection (Step 5 of the cycle) of priority actions through an initial screening process, corresponding to Step 4 of the climate-smart cycle. The goal of the second phase was to work with panels of subject matter experts to develop more detailed climate-smart designs for three of the management actions, corresponding to Step 6 of the climate-smart cycle; these would later be discussed with community members prior to implementation. Through these two applications, we were able to more comprehensively evaluate the tool in a real-world context.

Of the 14 actions in Table 1, actions 1 through 8 were related to watershed management, actions 9 and 10 to aquatic ecosystem protection, and actions 11 through 14 to aquatic ecosystem restoration. Actions 11 and 12 focused on the establishment of land-based fish and coral nurseries; although terrestrial, nurseries were identified as needing adaptation to climate change because of the threat to infrastructure posed by increasingly powerful storms. In the case of actions 13 and 14, there was insufficient information on coral reef resilience to guide decisions on fish releases and outplanting of corals during the first phase, so action 14 was only addressed during the second phase when resilience information became available (see Coral reef resilience assessment section below). Action 13 was never run through the tool.

One of us (DAG) completed the ADT Activity 1 worksheets for the actions 1 through 12 during the first phase, taking 3–4 hours on each action. Resources used included (1) the existing watershed management plan [21], (2) local reports and background resources on each action, (3) a Puerto Rico climate change vulnerability assessment (prepared by author PB using Puerto Rico Climate Change Council reports [41] and based on the Local Early Action Planning Tool [42] (S1 Table), and (4) focused discussions with local experts for some information on potential actions (e.g., the design status of the wastewater treatment plant’s tertiary filtration wetland and the history of Guánica Lagoon). The tool was applied iteratively to the actions because responses to one action sometimes influenced or informed others. For example, several of the actions related to watershed management were intended to manage the same stressors, so information gathered for one action could sometimes be applied to others. For some of these watershed actions (e.g., water diversion techniques to reduce road erosion), the sole stressor being managed was sediment, while nutrients and toxic chemicals were additional stressors to be managed by other actions (e.g., restoring Guánica Lagoon).

To synthesize the output of the two ADT worksheets, we developed five-column summaries that consolidated outputs for each action into a row that could fit on a single page, rather than on the two pages generally required for the full ADT worksheets. The Guánica Bay watershed managers felt that these five-column summaries would be a preferable format for decision makers and community members who may prefer a more concise synopsis. They suggested that the five-column summaries be included in the bodies of reports in general and the full ADT output be placed in report appendices.

For the second phase, we selected two of the initial 12 management actions (Table 1) that were being implemented or that local managers were highly confident would be implemented: dirt road management in mountainous farms (action 7), and establishment of coral nurseries (action 12). With the reef resilience assessment now available (see next section), it also was possible to examine coral outplanting (action 14) as a closely related reef restoration activity supported by coral nurseries. Both dirt road management and coral reef restoration are long-standing priorities for the Guánica area because mountainous roads are recognized as major contributors to stream and coastal sediment loads [40,43–45] and there is a strong emphasis in the community to improve the health and recovery of reef communities.

The approach for this phase involved working with local experts to elicit the more detailed level of adaptation information needed for implementation planning. Two panels of six experts (one for dirt road management and one for coral reef restoration) were consulted (S2 Table). Experts were selected on the basis of having complementary skills and knowledge of southwest Puerto Rico, as well as combining local actors with scientists. They were fully aware of the existing conservation areas and existing management efforts.

We used the following structure for the discussions. In an initial 90-minute web meeting, we introduced the ADT and the case study. We also provided the results from the first phase on which the second phase would be built, plus the recent preliminary results of the resilience assessment of Puerto Rico’s coral reefs (see next section). We followed the introductory call with two 90-minute web discussions a few weeks apart, during which the authors facilitated discussion of the actions following the structure of the ADT worksheets. Our goal was to elicit as much information on the actions from the experts as possible, rather than achieve consensus among experts. The authors updated the ADT worksheets in real time using the conference system’s screenshare, enabling the experts to share their respective opinions on and reactions to proposed text in the ADT worksheets. During the two web discussions for each panel, we added increasingly more detail to the information from the first phase, with some revising of the focus of the first phase actions. Experts reviewed successive drafts of the worksheets and their comments were collated within the ADT output.

### Coral reef resilience assessment

Concurrently with the ADT case study, a desktop resilience assessment of Puerto Rico’s coral reefs was conducted [17] using data from the most recently completed NOAA National Coral Reef Monitoring Program (NCRMP) survey of Puerto Rico. Based on the methods of [46], it involved selecting seven expert-vetted indicators of coral reef resilience to ocean warming associated with climate change (percent live coral cover, coral diversity, percent algae cover, incidence of coral disease, thermal tolerance of hard corals, biomass of herbivorous fish, and benthic rugosity), then rescaling them so that all indicator sites (n = 103) were assigned fractional values between 0 and 1, with 1 being the site most resilient to thermal stress. The indicators at each site were averaged to calculate a relative resilience score for each site, and sites were ranked by resilience (S1 Fig). Each site was also assigned a relative score for the stressors of fishing pressure [47] and land-based sources of pollution, with the values scaled the same way as the resilience indicators. Land-based pollution was modeled using OpenNSPECT [48] for watersheds and a simple coastal dispersion model to estimate relative sediment loads at reef sites. Indicator values, stressor values, and the resilience score were all relative to the surveyed sites, not based on any absolute scale. Using this assessment, reefs most suitable for specific management actions (e.g. reef restoration or mitigation of land-based sources of pollution) can be identified by defining criteria for those actions based on the individual indicators, stressors, and/or resilience scores (called “management action queries”). For more information, refer to [17].

We presented a preliminary version of this Puerto Rico-wide assessment to both expert panels as potential input for their use of the ADT, with the intent of assessing how the resilience assessment helped inform the design of the management actions that were considered in detail (S1 Fig). Based on feedback from the coral reef restoration panel when they discussed the two coral restoration-related actions, we localized the Puerto Rico-wide assessment to the area surrounding Guánica Bay. For this, we used the same methods as [17], but included just the 15 NCRMP survey sites in southwest Puerto Rico that had the full suite of collected survey data, and rather than rescaling indicators and resilience to the “best” value for all of Puerto Rico, we rescaled among the 15 local sites. This approach allowed the Guánica sites to be considered relative to each other (rather than sites throughout Puerto Rico) region, making the approach more relevant for Guánica-area planning (S1 File and https://github.com/dagibbs22/Puerto_Rico_Resil_assmnt).

## Results

### Adaptation Design Tool

The main output of this case study is the adaptation information from the first and second phases of using the ADT, as well as the comparison between them. Tables 2 and 3 present combined and condensed versions of both the first phase (black text) and second phase (blue text) results for the selected actions. Table 2 summarizes the climate change effects on stressors (worksheet 1A), while Table 3 summarizes the climate change effects on the management action (worksheet 1B). The information from both worksheets culminates in Column B7, which is the climate-smart design (highlighted in Table 3). The full-length versions of the first phase results for all 12 actions are available in S3 Table, the results for the second phase are in S4 Table, and the five-column summaries for the 12 actions evaluated in the first phase are in S5 Table. Uncertainties in and limitations of existing data are found in the “Notes” columns of all tables.

**Table 2:** Worksheet 1A from the Adaptation Design Tool.

A1	A2	A3	A4	A5	A6	A7
Management action number	Existing management action	Target stressor(s)	Climate change effects on stressor(s): direction, magnitude, mechanism, uncertainty	Timing of climate change effects	Implications for effectiveness metrics and how to measure them	Notes
7	Use flow diversion structures and flow reduction practices (e.g., water bars, vetiver and rock check dams, culverts) to manage sediment from coffee plantation dirt roads. *Use water conveyance practices (e*.*g*., *water bars*, *culverts) to manage sediment from coffee plantation dirt roads* *NOTE*: *Some flow diversion practices*, *and road stabilization measures are included with water conveyance practices because implementing them together increases their effectiveness*.	Terrestrial sediment	• Storms may become more intense, leading to precipitation events that may erode dirt roads faster, particularly on steep slopes. The percent increases in erosion and runoff will likely be greater than the percent increase in precipitation. High magnitude, low uncertainty. • The rainfall threshold for erosion of dirt roads appears to be around 0.1 cm, with dirt released within 1–2 minutes (Ramos-Scharrón and Thomaz 2016). This threshold may be reached during a higher proportion of storms. High magnitude, low uncertainty. • Stormwater plumes may extend further into the ocean, impacting more coral reefs. High magnitude, low uncertainty. • Storms of sufficient intensity to release levels of sediment exceeding reefs’ tolerance may occur more frequently. High magnitude, medium uncertainty. • *Increased resuspension of sediment in coastal waters in between storms due to stronger waves*. *High magnitude*, *medium uncertainty*. • Road erosion may become more intermittent *due to longer periods of drought punctuated by larger storms*. *More intermittent storms could themselves cause larger loads per event because drier soil will erode more easily*. *On the other hand*, *hard pans could form on roads during dry periods*, *causing initial rain to run off without sediment*. Medium magnitude, medium uncertainty. • *There may be shifts in eroded sediment particle sizes due to larger storms*, *potentially including more clay particles which stay suspended in water longer and chronically expose reefs to sediment*. *Or*, *larger particles may be increased*. *Low magnitude*, *high uncertainty*.	• Increasingly violent storms are already occurring. • Storm intensity will likely continue to increase over the coming decades. • *Prolonged dry periods are already occurring*.	**Effectiveness metrics:** Targeted percent reduction of sediment loads originating from dirt roads in coffee plantations. Reduction in number of roads that need rebuilding or significant management after storms. *Amount of sediment resuspension*. *Frequency of roads requiring regrading*. *Effective lifespan of conveyance structures*. **Implications for effectiveness metrics:** *Loads following rain events will need to be reduced by a larger percentage to keep sediment from crossing reefs’ sediment tolerance thresholds*. *Because these may be crossed more often due to increased storm intensities*, *the impact of acute sediment loads may increase relative to the impact of chronic loads*. *Sediment resuspension will occur more often*, *potentially contributing more to the sediment exposure of reefs*. *Road regrading will be necessary more often due to increased frequency of larger storms*. *Lifespan of conveyance structures will decrease*. **Implications for how to measure effectiveness metrics:** Water quality monitoring stations should be located *at the site of management practices and* down-channel of dirt roads with and without altered water management strategies (for comparison). It will become more important to have long-term sampling that reflects extreme storms (including sampling during storms). Ideally, water flow around roads would be monitored during some storms to see how the management practices are working. Sampling will likely need to be able to record a broader range of loads. *With increasing sediment resuspension*, *more effort will have to be made to measure resuspension during and after storms*. *If road regrading is required often enough due to more frequent larger storms*, *it will not be an effective success metric*. *Perhaps the extent of road regrading needed will be more informative*.	• How much are 2-, 5-, 10-, and 25-year storms expected to change by 2050? • What is the total sediment runoff reduction target for reefs? • How much of a reduction in runoff from use of road maintenance practices is necessary to reduce runoff to levels that reefs can tolerate in conjunction with other management measures? • How do road maintenance measures interact with other mountain erosion measures (e.g., shade-grown coffee) to reduce sediment? • Roads really need to be monitored during storms to detect locations and timing of most severe sediment runoff. • *No management measures will protect dirt roads from large storms*. *There is a limit on how large a storm event dirt roads can be designed to handle*. *That limit may be reached more often under climate change*. • *Residence times of sediment particles around reefs outside Guánica Bay could determine the relative contribution to coral exposure of sediment resuspension vs*. *new sediment*. • *How will climate change affect the size of sediment particles mobilized*? • *Information on changes in runoff coefficients are necessary*. • *Pattern of sediment pulses on coffee plantation dirt roads under climate change is very important and needs exploration*. •
12 14	First and second phases: Collect corals and establish aquarium-based coral nurseries. *Second phase only*: *Outplant corals on reefs around Gilligan’s Island to protect the coastline*.	Coral loss from: • Warmer ocean water • Lower pH ocean water • Terrestrial sediment and nutrients • Sea level rise • *Storm damage (especially for acroporids)* • *Anchor damage* • *Spills from industrial facilities* • *Large vessel traffic*, *and resulting oil spills and sediment resuspension* • *Stormwater from urbanized areas* • *Ongoing development around the Bay* • *Nutrients from wastewater*: *septic systems*, *and bad connections to municipal systems*	• Warmer waters may increase bleaching episodes and disease outbreaks. High magnitude, low uncertainty. • Storms may become more intense, leading to precipitation events with more runoff carrying sediment and nutrients from land. The percent increases in erosion and runoff will likely be greater than the percent increase in precipitation. High magnitude, low uncertainty. • Stormwater plumes may extend further into the ocean, impacting more coral reefs. High magnitude, low uncertainty. • Sediment and nutrient runoff may be exacerbated by warmer air temperatures that are expected to render soils more erosion-prone. Medium magnitude, low uncertainty. • Storms of sufficient intensity to release levels of sediment and nutrients exceeding reefs’ tolerance may occur more frequently. High magnitude, medium uncertainty. • Sea level rise may occur faster than reef accretion, leading to “sinking reefs”. Medium magnitude, low uncertainty. • Ocean acidification could decrease successful recruitment if corals cannot find settlement sites (e.g., due to altered chemosensory abilities) or calcify (due to acidic conditions). It may also reduce existing colonies’ growth rates. High magnitude, medium-high uncertainty. • Sediment and nutrient delivery may become more intermittent *due to increased drought periods*. Medium magnitude, medium uncertainty. • *Ocean acidification could decrease reproduction because colonies will need to put extra energy into skeletal growth*. *High magnitude*, *medium uncertainty*.	• Temperature effects have already occurred, with increasing magnitude through mid-century. • Increasingly violent storms are already occurring. • Storm intensity will likely continue to increase over the coming decades. • *Acidification beyond coral optima may have already occurred for some taxa and is expected to worsen*.	**Effectiveness metrics:** Number of colonies in nurseries, number of climate-tolerant genotypes in nurseries, and colony survival in nurseries. *Survival and growth rates of outplanted colonies*, *considering maintenance of genotypic and phenotypic diversity*. *Use of outplanted colonies by other organisms*. *Sexual and asexual reproduction of colonies*. *Reduction in storm surge reaching coast*. *Reduction in coastal storm damage*. **Implications for effectiveness metrics:** *Survival rates over longer time periods (multiple years) may decrease due to episodic events*, *like storms or bleaching events*. *Growth rates may decrease and time until sexually reproductive may increase due to acidification*. *Settlement of other calcifying organisms among corals may be impacted by climate change*, *as well*. *Storm surge will become more extreme*. **Implications for how to measure effectiveness metrics:** *Monitor survival for longer after outplanting because of more episodic climate change-associated events*, *like bleaching or storms*. *Monitor longer for reproduction because of delayed sexual maturity*. *Survival surveys should occur immediately after relevant stressor events to characterize responses of outplants*, *requiring rapid-response monitoring*.	• What coral species are best to use? • *Is a certain level of rugosity of outplants desirable for maximum wave reduction*? • *Some micro-fragging is being used to “re-sheet” dead boulder colonies*, *like Orbicella*. • *Since this action is so closely related to the nursery action*, *it will be important to adjust nursery rearing practices in response to outplanting results*. • *To what extent will larger storms with longer droughts between increase the delivery of legacy PCBs to reefs*?

This covers climate change’s effects on stressors. First phase text is in non-italics. Material added during the second phase is in *italics*. Text is condensed and edited for this table, and actions are numbered is as in Table 1. The full worksheets from the first and second phases, including column heading descriptions, are in S3 and S4 Tables, respectively. For condensed summaries of the initial phase, refer to S5 Table.

**Table 3:** Worksheet 1B from the Adaptation Design Tool.

B1	B2	B3	B4	B5	B6	B7	B8
Management action number	Existing management action	Changes in effectiveness of management action due to climate impacts on target stressor	Changes in effectiveness of management action due to climate impacts on management action	Time frame or constraint for using the action and implementation (e.g., urgency, longer or shorter term)	What changes are needed to adapt the action (place, time, and engineering design)	Climate-Smart Management Action	Notes
7	Use flow diversion structures and flow reduction practices (e.g., water bars, vetiver and rock check dams, culverts) to manage sediment from coffee plantation dirt roads *Use water conveyance practices (e*.*g*., *water bars*, *culverts) to manage sediment from coffee plantation dirt roads* *NOTE*: *Some flow diversion practices*, *and road stabilization measures are included with water conveyance practices because implementing them together increases their effectiveness*.	• Water diversion structures may not be able to divert all water off roads, producing downhill road erosion. • Flow and erosion reduction practices may not be able to slow down water sufficiently to prevent further erosion and trap sediment. • Vertical dirt walls along roads may be more likely to collapse under increased precipitation. *Sediment trapping structures may not be able to handle the resulting increased loads*. • Interstices between stones in check dams or stone swales may become clogged with sediment more quickly from larger or more frequent storms. *This will reduce the cross-sectional area available for water to flow through and the area available for conveying water*.	• Larger storms may wash out existing water conveyance structures (e.g., water bars, the stones comprising check dams, *or culverts*). • Larger storms may wash out existing flow and erosion reduction practices (e.g., vetiver and rock check dams). • Larger storms may overflow culverts or wash them out entirely, *leading to stream crossings being washed out*. • Road regrading may be complicated by increased risk of raised road banks slipping onto road during or after regrading. Slippage is already happening but could happen more under some precipitation scenarios. • *Droughts may compromise the effectiveness of vegetative solutions*. *Vetiver probably does fine in droughts; native plants (investigated by the US Fish and Wildlife Service) may not do as well*. • *Work completed less than one week to one month before a large storm may be undone*, *i*.*e*. *a completed project can take a month to establish*. *Depending on the pattern of large storms*, *this could increase*, *decrease*, *or simply shift the window for completing projects*.	This suite of actions can be implemented immediately. They have the potential to quickly affect sediment loads.	• Compact dirt roads that have been topped with aggregated crushed stone. • Make road crown higher to consistently drain water to sides of road, if not using insloped/outsloped roads. • Implement stronger/more frequent debris barriers upstream of culverts to prevent culvert clogging. • Use larger rip-rap stones or more extensive vetiver patches on downstream sides of culverts to diffuse faster and larger flows. • Increase frequency of rolling dips or water bars on steeper-sloped roads or stretches likely to experience heavy erosion. • Clean accumulated sediment out of interstices between sediment trap rocks more frequently. • Roads on particularly steep slopes may need to be paved (with asphalt or concrete) if other management actions are not working. • *Increase the size of culverts to some standard minimum size that will handle storms of a specified size (e*.*g*., *the predicted 5-year storm)*. • *Build maintenance into construction of the projects*. *Maintenance will have to be more frequent*. • *Use vegetation that can withstand both dry periods and stronger flows*.	Minimize sediment from existing dirt mountain roads by building water diversions more frequently along roads, sloping roads more heavily to promote faster drainage, and augmenting barriers on upstream sides of culverts and flow diffusers on downstream sides of culverts. *Culvert size should be increased to a standard minimum size in preparation for consistently larger flows*. Locations requiring flow control may change due to altered precipitation patterns. Check integrity and repair diversion structures after larger storms; remove sediment from sediment traps after large storms. Compact roads with surfaces made of small rocks and granular material to stabilize road surfaces. Pave roads that have already repeatedly washed out if other mitigation techniques are not possible.	• These structures and practices are considered together (as a suite of actions) because they must be implemented in combination in order to be effective. • To what extent can dirt roads traversing hills and mountains be retrofitted? • Which roads are most important to work on? • This is a feedback loop: the worse the road water management actions perform, the more erosion there will be, and the worse they will perform. • *When is paving dirt roads an option*? *Are they generally too ephemeral to justify paving*? • *Dirt roads can be stable for a long time if managed properly*. *However*, *once they begin to degrade*, *they can fall apart very quickly*. *They have a failure threshold and maintenance keeps them from reaching that threshold*. • *Road maintenance is not generally funded in grants*, *yet it is essential for long-term performance of structures*. *Thus*, *maintenance falls to the property owners (farmers)*. *Pretty much all maintenance will be affected by climate change*. • *No-cost extensions would increase flexibility in completing work under greater weather uncertainty*. • *Climate change may reduce the lifespans of projects*. *Standard lifespan now is 25 years*. • *Project locations are based on which farmers are willing to collaborate*, *as well as slope*, *potential of land to erode*, *connectivity to water bodies*, *and traffic load*. *To what extent will climate change affect these*? • *Supply farmers with culverts of the right size so they are not just using whatever they have handy*.
12 14	First and second phases: Collect corals and establish aquarium-based coral nurseries. *Second phase only*: *Outplant corals on reefs around Gilligan’s Island to protect the coastline*.	• Fewer fragments of coral colonies (especially of certain species) will be available for collection after storms due to reduced coral cover. • On the other hand, opportunities for collecting new nursery stock may increase since stock will only be collected after storms (and at construction sites). • Available coral colonies may be more resistant to higher temperatures and existing diseases (through natural selection). • Propagating coral genotypes without regard to their resilience to climate stressors will reduce action effectiveness because individuals in the nursery will have the same tolerance of climate change conditions as wild corals. • *Outplanting sites selected because they are currently in deep water may be in too deep water in the future due to sea level rise*. • *Turbidity or coastal erosion plumes/hotspots may change in such a way that outplanting locations selected to avoid hotspots or plumes may no longer do so*. • *Outplanting locations not currently exposed to legacy sediment contaminants could become exposed from increased resuspension or runoff*. • *Decreased growth and reproduction rates of outplants may occur from ocean acidification*. • *Above stressor effects may reduce the genetic diversity of outplanted colonies below the intended level*.	• Climate change may physically damage or destroy nurseries through more powerful storms. • *Nurseries must be prepared for power failures*, *loss of clean water*, *and other emergencies*. • *Increased physical destruction of outplanted colonies by storms*, *especially for more delicate species* • *Increased death of outplanted colonies from bleaching and diseases*.	• It will be 2–4 years before corals can be outplanted, so this is a medium-term action. It should probably be started immediately, to reduce long-term coral losses. On the other hand, if coastal water quality does not improve, outplanted corals may not survive. • *The sooner this is done*, *the better* • *Outplanting will have to be a sustained*, *long-term effort*.	• Preferentially grow coral genotypes that are disease-, heat-, and sediment-tolerant/resistant. In general, grow genotypes that will be able to survive projected future conditions. As conditions change further on reefs (e.g., disease outbreak, major sedimentation event), collecting new coral fragments for the nursery will introduce colonies that have survived the latest round of selective pressure. • Preferentially grow species that will restore ecosystem function by building up a protective reef structure. • Due to increased coral mortality from bleaching and disease, collect more colony fragments due to higher mortality of outplanted colonies. • The similarity between the water supplying the rearing aquaria and the place(s) where the corals will be outplanted also needs to be considered. Presumably, these should be similar in temperature, pH, and chemical composition. • *Focus on outplanting coral strains with a variety of types of tolerance to climate-change effects*, *including being bred to tolerate multiple stressors*. • *May need to focus on outplanting at shallower sites due to sea level rise*, *although that must be balanced against the impacts of larger storm and terrestrial runoff at shallower depths (in general)*. • *If outplanting in deep water*, *focus on using species with a broad depth range to account for sea level rise*. • *Outplant some aquarium-bred colonies with the hope that they will be naturally fragmented and propagate themselves*, *and outplant others to build up reef where they are*. • *Cement may need to be used more often to attach colonies to substrate due to increased risk from storms*. • *Time outplanting to avoid periods of enhanced runoff and land-based pollution*. • *Factor changes in sediment plume location and direction into outplanting site selection*. • *Monitoring may need to be extended longer after outplanting to include observation of how outplants handle extreme events and to what extent they reproduce*.	Develop multi-species aquarium-based coral nurseries which can produce a continuous supply of coral colonies through repeated fragmentation. Species that can survive the current temperature, pH, sediment and nutrient regime should be used until water quality is restored. This may involve collecting new colony fragments that have survived widespread bleaching or disease or survived large sedimentation events. Coral strains should be heat-tolerant (to reduce risk of bleaching) and show some resistance to the relevant coral diseases that are associated with higher temperature. They should also be effective at removing deposited sediment and maintaining growth in lower pH water. Water used in the aquaria should be from the general area where the corals will be outplanted. *Outplant colonies that are heat-*, *disease-*, *sediment-*, *and low pH-tolerant*. *Species mix should be optimized for robust coastal defense*. *Outplanting during high sediment or precipitation periods should be avoided to reduce initial stressor exposure to outplants*. *Balance outplanting locations between deeper and shallower sites*, *accounting for how sea level rise may make deeper sites inhospitable in the future and how shallower sites may be more heavily exposed to land-based pollution*. *Colonies in shallower sites may need to be affixed to reefs using cement more frequently due to larger storms*. *Colonies in deeper sites should have a broad range of depth tolerances*. *In either case*, *it may be desirable to locate branching colonies where they will be naturally fragmented*. *Site selection may also be affected by shifting plumes of land-based pollutants from the bay*. *Because survival through extreme events is an important part of the nursery program*, *colonies should be monitored through extreme events such as high temperatures and large storms*. *Monitoring periods for outplants may need to be extended to do this*.	• Preferentially grow corals that are disease, heat-, and sediment-tolerant/resistant. In what order should those traits be prioritized or how should they be balanced? Many organizations are working on creating hybrids that combine these traits. • The nurseries must be large to have an ecologically meaningful amount of coral in them. How much is that? • *Can bathymetry be used to predict where storms will be most or least damaging*? • *Reefs to the west of Guánica Bay receive more sedimentation than those to the east*. *Reef resilience assessment shows some potentially suitable sites to the east of the bay*. • *Most people are attaching colonies individually at this point*. *What can be done to usefully attach multiple colonies simultaneously*? • *Need to focus on other activities that are important for improving the outplanting environment*.

This covers climate change effects on the management action and implications for climate-smart design. First phase text is in non-italics. Material added during the second phase is in *italics*. Text is condensed and edited for this table, and actions are numbered is as in Table 1. The full worksheets from the first and second phases, including column heading descriptions, are in S3 and S4 Tables, respectively. For quick summaries of the initial phase, refer to S5 Table. Column B7 is highlighted because Worksheets 1A and 1B culminate in this description of the climate-smart management action.

#### First phase results

Because the eight watershed-focused actions addressed similar stressors, the tool outputs were similar for the effects of climate change on stressors (worksheet 1A); however, the outputs diverged for the effects of climate change on management actions and how to adapt (worksheet 1B) (S3 Table). For the most part, the watershed-focused management actions (e.g., management action 7 in Tables 2 and 3) centered on adapting to changes in precipitation patterns due to climate change and being able to mitigate impacts from both drought and flood conditions. Indeed, most of the actions had notes about similar information gaps in precipitation projections, suggesting that the same research could be used to fill knowledge gaps for many of the actions. The actions also had similar monitoring concerns, such as capturing action effectiveness under the full range of stressor loads during storms of increased intensity. The importance of adaptive monitoring to measure the effectiveness of actions was highlighted as a priority.

One key piece of information that was consistently missing from the watershed management actions was what the target land-based pollution reduction should be for a given action at a specific site, as well as for all actions in aggregate. Part of the uncertainty was due to a lack of information on the historical and projected effects of land-based pollution on coastal ecosystems, including coral reefs. Potential effects on coral reefs, and uncertainties about those effects, were included in the watershed actions insofar as climate change affects how runoff is distributed in the ocean (plume extent, frequency, particle size, etc.) and how often reefs’ tolerance thresholds for land-based pollution might be exceeded.

The final outputs (descriptions of climate smart versions of the actions) included not just changes to actions’ physical sizes (i.e., climate change adaptation does not simply require making the action physically bigger), but also included changes to siting and construction methodologies or materials. Additionally, many of the climate smart actions specifically identified adaptations that might be needed for maintaining the action, not just adaptations in the construction. For example, hydroseeding bare soils will require more follow-up to sustain soil coverage (S3 Table). Because the exact location of where most of these actions would be implemented was not specified in the sources they were extracted from, the adaptations were based on general principles for prioritizing locations (e.g., soil types or slopes). Finally, some of the climate-smart actions had to address the possibility of a wider range of levels due to fewer or stronger storms (e.g., restoration of Guánica Lagoon). This was accomplished by including recommendations for vegetation that were both drought-resistant and well-rooted.

The first-phase focus for coastal actions (seagrass and mangrove protection, creating larval fish nurseries, creating coral nurseries) was altering the location of interest (habitat protection) and target species and traits (fish and coral nurseries). As with watershed actions, the two habitat protection actions and the two nursery actions were more similar in worksheet 1A (effects of climate change on stressors) than in worksheet 1B (effects of climate change on management actions and how to adapt). For targeting the action location, emphasis was placed on maximizing intra- and inter-habitat connectivity under climate change, filtering/trapping land-based pollution, and identifying habitats that could serve as climate refugia while accounting for existing conservation areas (S3 Table). Because sea level rise is a threat to both mangroves and seagrasses, it was considered a protection criterion for both resources. For the nurseries (actions 11 and 12 in Table 1), the focus was on how climate change could affect continuous operation of the nurseries during extreme events, the acquisition of fish and corals for the nurseries, and what species and traits to genetically select for propagation. Terrestrial nurseries are expected to be threatened, and their operations interrupted, more often by large storms and hurricanes, and therefore require additional emergency abilities (e.g., how to handle lack of clean water and prolonged power failures). Aside from resilience of the nursery facilities themselves, the water in the aquaria should be as similar as possible to the water at the outplanting sites (e.g., temperature, pH, sediment loads). In general, adaptive traits for corals and fish were theorized to include lower sensitivity to high temperatures, tolerance of more acidic water, and disease resistance (for corals). Finally, a key precondition for these two actions was noted: *in situ* water characteristics and habitat must be of sufficient quality to make undertaking these worthwhile. If local stressors are not addressed and there is no suitable habitat into which to release nursery-raised fish or corals, the effort put into the nurseries may be better directed elsewhere.

#### Second phase results

During the second phase analysis by the expert panel, the focus of the actions was refined and shifted based on new considerations (Tables 2 and 3, S4 Table). For dirt road management in coffee farms, the experts wanted to focus on a narrower set of actions, so they shifted from flow diversion and flow reduction practices to water conveyance practices. However, they noted that water conveyance, flow diversion, and flow reduction practices are often implemented concurrently, so they ultimately included a broader suite of interventions in their discussion. In order to show development of more location-specific detail using an example, the experts discussed a farm known to have owners who were interested in erosion control and would be favorably inclined to make their plans more focused on adaptation. While using that farm as a specific case, the experts also generalized their observation to other locations with similar conditions during the discussions.

After narrowing of the types of water management actions under consideration, the road management expert panel generally agreed with the first phase information but added new details as well as important research needs captured in the Notes (Tables 2 and 3). For example, they proposed a few situations where climate change could affect the system in divergent ways. One such situation was whether a hard crust of dirt (hard pan) might form during dry periods, thereby reducing sediment loss during some ensuing storms. They also discussed changes in sediment particle sizes due to changing precipitation patterns (including resuspension on coral reefs), although they concluded there was not enough information for them to make any predictions about how sediment particle sizes would change. They also emphasized the potential reduction in project lifespans under climate change due to more rapid wear and greater chances of destruction, and that those revised lifespans must be built into project planning and budgets. Similarly, they noted that maintenance needs for projects would likely increase and would need to be accounted for early in project development. Just as in the first phase, they were not able to assign a target percent reduction for sediment loads from dirt roads in coffee farms because too little is known about the system. Finally, prompted by their experience during Hurricane Maria (2017), they acknowledged that adaptation to storms above some intensity is infeasible, although they did not identify where that threshold would occur.

For the coral reef restoration action, the experts discussed both nursery creation (included in the first phase) and outplanting steps of coral restoration (not included in the first phase) because they are so closely related and because outplanting was of particular interest to coral reef managers in Guánica. The outplanting action had not been considered in the first phase because it was judged that a resilience assessment was needed for effective site selection before designing an outplanting scheme. However, with the help of the draft resilience assessment that was completed between the first phase and the second phase (see resilience assessment results below), the experts were able to choose an outplanting location and objective for restoration using the resilience assessment’s suggestion of suitable sites for targeting interventions. Hence, they expanded their focus to examine outplanting of corals (action 14 in Table 1) around Gilligan’s Island (Cayo Auroro) with the objective of protecting the coastline from erosion. Gilligan’s Island, located a mile off the coast of Guánica, is part of the Biosphere Reserve of Guánica, and is managed by the Puerto Rico Department of Natural Resources. Although there are no reef resilience assessment sites at Gilligan’s Island, it is relatively near and shares characteristics with one of the survey sites that was identified as suitable for restoration.

For the coral reef restoration second phase, the experts emphasized ocean acidification and legacy sediments more heavily than was done during the first phase analysis. They also noted that site conditions are changing directionally (lower pH, higher temperature) and that nursery conditions need to reflect those ongoing shifts. Their proposed success metrics included maintaining genetic diversity of corals, benefits throughout the full life cycle of corals, the community consequences of restoration (e.g., functional roles served by outplants for other taxa), and reductions in storm surge and coastal damage. The experts were more specific about the traits that corals will need under climate change, including heat tolerance, sediment removal, and maintaining growth in low pH water, and how there might be tradeoffs among these. They proposed conducting long-term monitoring that would capture effects from the rare, large storm or bleaching event and would be highly responsive to such events. In terms of the siting for outplanting, they noted the need to account for potential shifts in locations and extents of sediment plumes under climate change, although they did not have specific predictions or recommendations for the study area. A final theme was the depth for out-planting corals, recognizing tradeoffs between risks from increased storms and temperature at shallow depths versus limits of each species depth range.

### Coral reef resilience assessment

**Fig 4:**
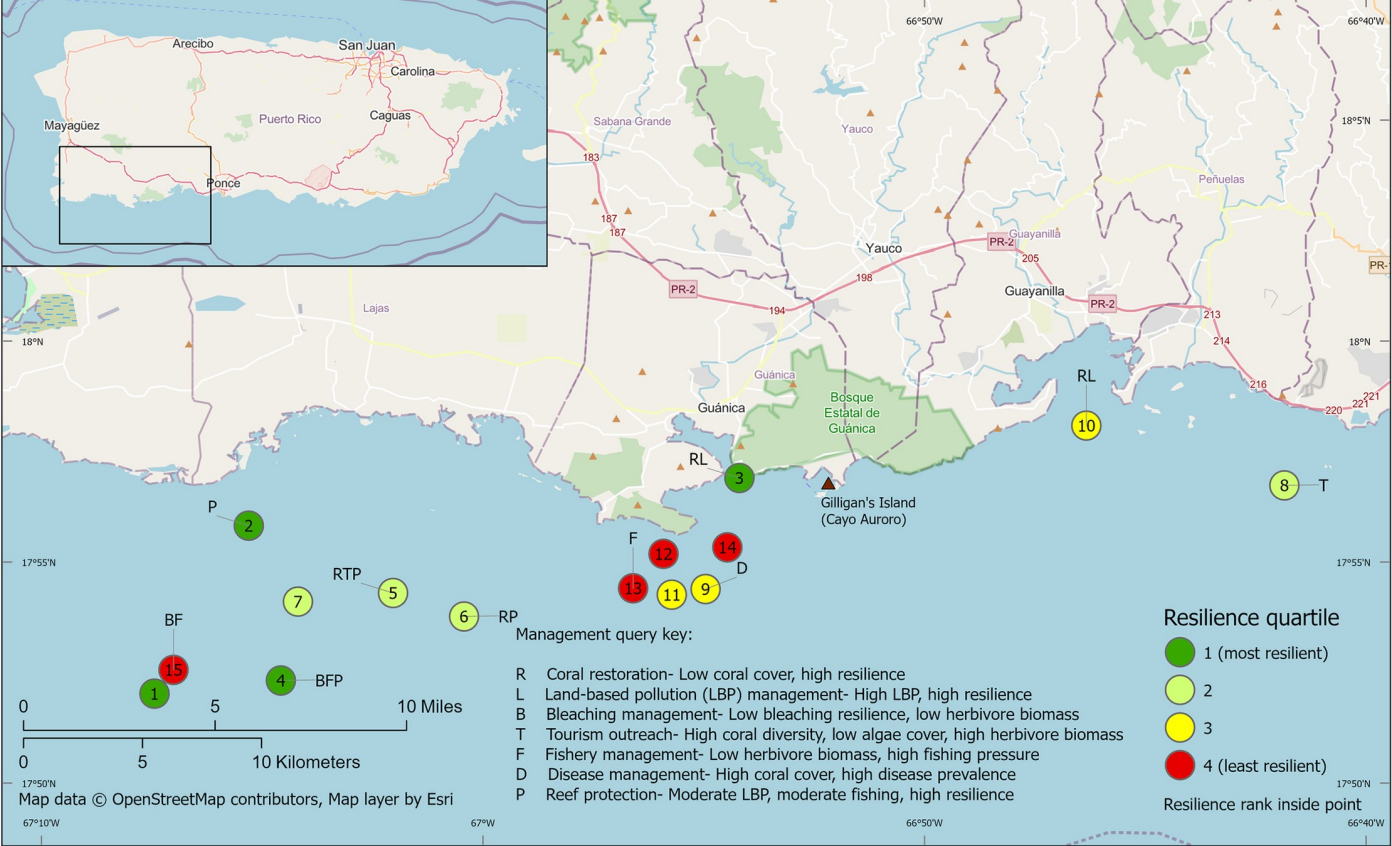
Reef resilience assessment for southwest Puerto Rico, including sites’ resilience ranks (1–15) and potential management actions at each site.

The road design expert panel acknowledged that watershed management activities like road design affect coral reefs, but the group was primarily interested in practices to reduce overall erosion from dirt roads rather than how road specifications were related to reef resilience; thus they did not see a map of reef resilience as informative for road design. In contrast, the coral restoration expert panel did use the coral reef resilience assessment to identify areas of high and low resilience and areas that might be good restoration opportunities based on pre-determined criteria for selecting coral restoration sites (management query, per [17]). However, they noted that the Puerto Rico-wide coral reef resilience assessment was too large-scale for reef actions focused on Guánica, prompting us to localize the resilience assessment to just the Guánica region (Fig 4). This approach allowed the Guánica sites to be considered relative to each other (rather than relative to all sites throughout Puerto Rico), making the approach more relevant for Guánica-area planning. One of the two sites that fit the criteria for coral restoration was just to the east of the mouth of Guánica Bay (number 3 in Fig 4), not far from Gilligan’s Island, the site that the restoration panel considered for the second phase. It likely would not receive much land-based pollution from the Guánica watershed because the major ocean currents in this area flow to the west. The next steps after identifying this specific survey site as having high potential for restoration based on resilience would be to discuss with stakeholders and confirm benthic habitat suitability through field investigations.

## Discussion

This is the first application of the ADT during a management planning exercise. To that end, selecting potential management actions, running them through the tool as a first phase, and determining how best to summarize them (e.g., five-column summaries, S5 Table) for use in evaluation and prioritization was novel. Likewise, this is the first application of the ADT to explicitly produce the detailed level of information needed to carry out the implementation step of the climate-smart planning cycle (Step 6 of the climate-smart planning cycle, Fig 1). This dual use of the ADT allowed for a comparison between the pre-climate-smart management actions, first phase management actions made partially climate-smart by a single non-expert, and second phase actions made more thoroughly climate-smart by groups of experts. The generalities of the first phase outputs lend themselves to more regionally applicable guidance on making management actions climate smart that can be further customized and elaborated upon for specific locations; the outputs could serve as reference material for related actions in similar situations. We also identified the extent to which the ADT can contribute to planning for implementing actions; experts can provide significant details towards implementation while using the tool, assuming the action is sufficiently specific in location and objective. These outputs need to be further discussed with local stakeholders if they have not already been included during the use of the ADT to make sure stakeholder knowledge is reflected and buy-in is achieved. The integration of stakeholder knowledge with expert use of the ADT during project implementation is an opportunity for further research.

The two expert panels found the ADT worksheets useful for systematically evaluating the potential impacts of climate change on intended management actions and for working through adaptation strategies. During the two 90-minute discussion sessions for each panel, the experts added considerable detail to the first phase of the actions and moved toward plans for action implementation for specific locations in the Guánica region. The experts provided in-depth knowledge on the limits of management actions (e.g., when it is just not possible to have a large enough culvert to mitigate more intense/frequent storm runoff) and the timing needed for actions to be effective.

We also explored how spatial resilience assessments could be used in conjunction with the ADT. The climate-smart cycle indicates that information on resilience along with vulnerability are inputs to Step 4 of the climate-smart cycle (identifying adaptation options); however, direct use of a resilience assessment in identifying adaptation options had not previously been tested. While spatial patterns of coral reef resilience were not deemed relevant to road design, the coral reef restoration expert group used the island-wide resilience assessment to discuss outplanting design, providing insights into the connection between spatial resilience data and the ADT. There are two general ways in which resilience data can inform use of the ADT: site selection and identifying and designing climate-smart management actions.

First, maps of relative resilience can be used to select priority sites for management activities, whether the objective is to maintain resilient sites or to improve less resilient sites. For example, the survey site just east of the mouth of Guánica Bay has the third-highest resilience based upon multiple contributing factors. The prevailing ocean currents are driven by trade winds and flow westward, transporting effluent from Guánica westward towards La Parguera [31,49]. The Guánica State Forest (Bosque Estatal de Guánica) and UNESCO Biosphere Reserve extend to the east of Guánica Bay. The Guánica Dry Forest has been a protected ecosystem with minimal anthropogenic land use (United Nations Biosphere Reserve since 1981 and a commonwealth forest since 1917) [50]. The Guánica State Forest also manages Punta Ballenas Reserve, which contains mangrove forest, submerged aquatic vegetation and coral reefs, and the Cayos de Caña Gorda, a group of three uninhabited, mangrove-covered cays. The natural, native terrestrial landscape, protected mangroves, seagrass and coral reefs, combined with the westward ocean currents contribute to the high resilience of these coastal systems. Aware of these conditions contributing to the relatively high resilience of the site, the experts chose one of the nearby three cays (Gilligan’s Island) as the focus for reef restoration (Fig 4).

Second, in terms of identifying and designing actions, the management queries of the resilience assessment help managers identify which actions can maintain or improve resilience at a given site. The ADT can then be used to design those actions so that they are successful under current and future conditions. For example, the Gilligan’s Island site is indicated for coral restoration, so restoration interventions in this location would then be put through the ADT. Information on the relative resilience of candidate sites—and what is conferring that resilience—also can be essential for aiding climate-smart management action design. For example, ADT worksheets for activities involving acquiring resilient corals (e.g., collecting corals for nurseries and outplanting, or identifying potential refugia for protection) ask for what is known about the location of resilient corals and/or the condition of the site with regard to key factors that support resilience (such as healthy herbivore populations). This explains why, in this study, the resilience maps were not as helpful for experts whose main objective was reducing sediment loads throughout a watershed that had already been selected (e.g., Guánica Bay). However, the resilience maps were of greater interest to the expert group focused on setting up nurseries and outplanting corals; for them, the resilience maps provided some clues as to where resilient corals might reside and be collected for propagation, and what sites are already resilient and ready to receive outplants.

A different extent of resilience assessment might be needed when considering how to reduce sediment throughout the watershed. In natural systems, multiple processes operate simultaneously at numerous spatial and temporal scales [51–53], and patterns or relationships observed at one scale may be invisible when examined at another scale [54]. Climate change adaptation requires consideration of the broader landscape context [5]. In this case, consideration of the number and location of dirt road restorations required to protect/increase coral reef resilience could be a follow-on study. Additional information at the watershed scale would be required, including locations of all dirt roads, identification of which streams would be receiving the stormwater runoff from the dirt roads and how that would affect the streams themselves, and how much water could be diverted from the dirt roads under various rainfall scenarios. This could be added to the resilience assessment as a best practice for those facilitating the process. The alignment of scale and extent between the resilience assessment and ADT is one difficulty that future researchers and managers should consider early in the management cycle.

Finally, it is important to note that use of resilience assessments and the ADT is iterative. As resilience assessments are updated, it can signal to managers when site selection and selected actions need to be re-evaluated. Likewise, as new actions or locations are evaluated and selected, a new resilience assessment may be needed, including updated and/or new types of data. The management action designs crafted with the tool are only as good as the information available to managers (e.g. vulnerability and resilience assessments), but the ADT can be used iteratively as new information becomes available.

In conclusion, the use of the Adaptation Design Tool in conjunction with a coral reef resilience assessment is a proof-of-concept consistent with adaptive management, a key component of which is to proceed with the best information available and work iteratively. We have demonstrated how a spatial resilience assessment can (1) contribute to identifying sites at which to prioritize adaptation actions and (2) help with designing actions. The ADT leverages existing information to more effectively protect and restore valuable natural systems such as watersheds and coral reefs, and combines that information to provide insights into adaptation. As the expert panels noted, the value of convening an interdisciplinary group of experts to tackle a complex problem using a logical decision process is as valuable as the resulting information itself. This process of combining multidisciplinary information on resilience, vulnerability and their implications for adaptation design supports robust decision making within the context of the uncertainties associated with climate change, by using the best available information while allowing the flexibility for continued improvements in the future.

## Supporting information

S1 Fig Puerto Rico-wide coral reef resilience assessment. The 103 sites are from the NOAA National Coral Reef Monitoring Program 2014 survey. Sites are shown by resilience rank (1 is most resilient, 103 is least resilient) and resilience quartile. *Reprinted from [17] under a CC BY license, with permission from PLoS One, original copyright 2019*.
(DOCX)

S1 Table Vulnerability assessment for Guánica Bay watershed and associated coral reefs (prepared by author PB for expert consultations described in [15]. (DOCX)

S2 Table The experts who participated in the second phase panels. Institutional affiliations are those at time of panel meetings (summer 2017).
(DOCX)

S3 Table First phase output for Adaptation Design Tool Worksheets 1A (effect of climate change on stressors) and 1B (climate change effects on the management action and implications for climate-smart design) for 12 management actions for the Guánica Bay watershed and associated coral reefs. (DOCX)

S4 Table Second phase output for Adaptation Design Tool Worksheets 1A (effect of climate change on stressors) and 1B (effects of climate change on management actions and how to adapt management actions) for three management actions for the Guánica Bay watershed and associated coral reefs. (DOCX)

S5 Table Five-column summaries of Adaptation Design Tool Worksheets 1A and 1B (effect of climate change on stressors and effects of climate change on management actions and how to adapt management actions, respectively) for 12 management actions for the Guánica Bay watershed and associated coral reefs. (DOCX)

S1 File R script for conducting the southwest Puerto Rico resilience assessment. (RMD)

S1 Data Inputs and outputs for southwest Puerto Rico coral reef resilience assessment. Spreadsheet includes raw indicator values, rescaled indicator values, stressor values, resilience ranks and quartiles, and more. These data were used to make Fig 4.
(XLSX)
