# Supplementary material for: Incorporating adaptation and resilience into an integrated watershed and coral reef management plan
Source: PLoS One. 2021 Jun 24;16(6):e0253343. doi: 10.1371/journal.pone.0253343 (PMC8224911; doi:10.1371/journal.pone.0253343)
Supplement: S1 Fig — The 103 sites are from the NOAA National Coral Reef Monitoring Program 2014 survey. Sites are shown by resilience rank (1 is most resilient, 103 is least resilient) and resilience quartile. Reprinted from [17] under a CC BY license, with permission from PLoS One, original copyright 2019. (DOCX) [file pone.0253343.s001.docx]

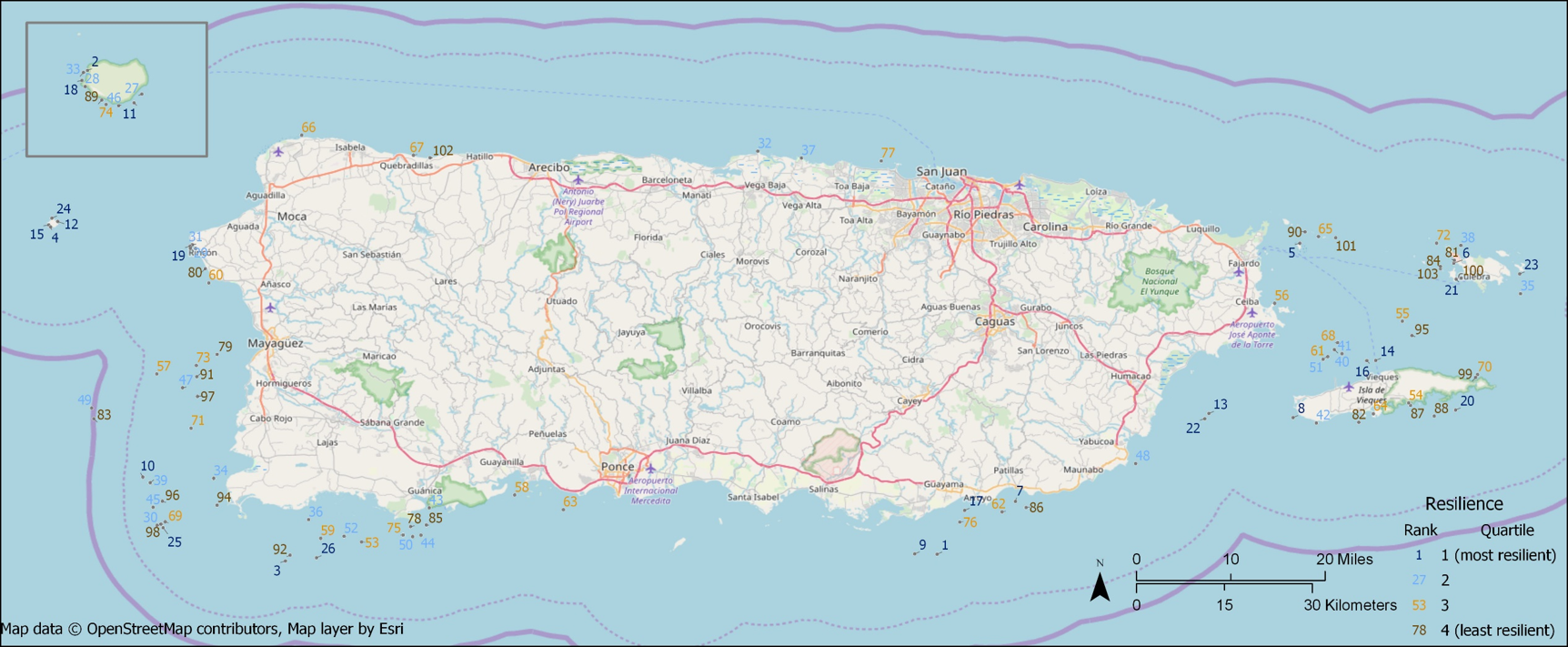


**S1 Fig. Puerto Rico-wide coral reef resilience assessment.** The 103 sites are from the NOAA National Coral Reef Monitoring Program 2014 survey. Sites are shown by resilience rank (1 is most resilient, 103 is least resilient) and resilience quartile. From Gibbs and West 2019.
