# Supplementary material for: Incorporating adaptation and resilience into an integrated watershed and coral reef management plan
Source: PLoS One. 2021 Jun 24;16(6):e0253343. doi: 10.1371/journal.pone.0253343 (PMC8224911; doi:10.1371/journal.pone.0253343)
Supplement: S1 Table — (DOCX) [file pone.0253343.s002.docx]

**Table S1. Vulnerability assessment for Guánica Bay watershed and associated coral reefs (prepared by author PB for expert consultations described in West et al. (2018)**

| **COMMUNITY NAME:** *Guánica Bay Watershed* | | | |
| --- | --- | --- | --- |
| **Indicators of a Changing Climate** | | | |
| **Climate Threat** | | | **Impacts** |
| **Indicator** | **Magnitude and direction of change over time based on community knowledge and latest climate science** | **Changes in environmental conditions**  **(Climate Stressors)** | **Potential impacts to natural and social resources** |
| - **Air temperature** | **Air temperature has increased and is projected to continue to increase in Puerto Rico.**  *Historical:* Mendez (2010) showed that twelve of sixteen stations in Puerto Rico had significant increases in annual average temperature from 1948 to 2007. Statistically significant (p<0.05) increases in monthly average temperature trends are observed for most months and stations. From these results, Mendez (2010) concluded that the annual temperature increased over this period throughout Puerto Rico. The number of very warm nights in Puerto Rico has increased by 15 or more per year from 1950 to 2004 (USGCRP 2009). Additionally, the NOAA National Weather Service’s 113 yr record shows a lower frequency of days with temperature equal to or below 75^o^F. Climate Forecast System Reanalysis Temperature trends shows that the central (elevated) parts of Puerto Rico are warming faster than the coasts. However, in the San Juan region, warming is attributed at least in part due to the urban heat island effect (Velazquez-Lozada et al. 2006; Gonzalez and Comarazamy 2009).  *Projected:* Projections for Puerto Rico show as little as 0.02°C/year warming through 2050, in other words at least 0.8 °C by mid-century. | *Warmer temperatures, higher rates of evapotranspiration, changes in rainfall patterns with potential increase in drought conditions* | *Shifts in composition and distribution of native and non-native species, leading to losses of soil-stabilizing vegetative cover that could result in increased soil erosion* |
| - **Sea-surface temperature (SST)** | **Sea surface temperature has increased and is projected to continue to increase Puerto Rico***.*  *Historical:* Caribbean SST has warmed by 1.5°C over the last century (IPCC 2007), and by 0.27°C per decade over the period 1985 – 2009 (PRCCC 2013). SST warming trends south of Puerto Rico are faster than to the north and sub-surface temperatures are warming faster than the surface, particularly south of Puerto Rico. The higher rate of warming south of Puerto Rico is related to a significant weakening of trade winds, evaporation and westward currents (Jury 2011).  *Projected:* The observed warming trend is projected to remain similar in the 21st century. SST above the threshold for coral bleaching will be exceeded over a third of the year (Hoegh-Guldberg 1999). | *Warming seas, changes in ocean stratification* | *Coral bleaching and potential loss of reef structure and associated fish; shifts in marine species distribution and migration patterns; impacts to fishing sector* |
| - **Sea level** | **Sea level has risen and is projected to continue to rise in Puerto Rico.**  *Historical:* Tide gauge records from Isla Magueyes (south coast of PR) and from San Juan (north coast of PR) contain the longest sea level time series in the U.S. Caribbean (56.7 and 49.4 years respectively). Analyses show a rise of at least 1.4 mm/year (PRCCC 2013).  *Projections:* If the observed Puerto Rico sea level rise trend continues linearly, with no acceleration in rate, by 2100 the sea level around Puerto Rico will have risen by at least 0.4 meters. One analysis projected possible future sea level rise estimates ranging from 0.07 to 0.57 meters (0.20 to 1.87 feet) above current mean sea level by the year 2060 and between 0.14 and 1.70 meters (0.40 to 5.59 feet) above current mean sea level by the year 2110. Based on this information and future projections for sea level rise the PRCCCC recommends planning for a rise of 0.5-1.0 meters by 2100. | *Increased storm surges and king tides, more frequent coastal inundation, larger areas of inundation, greater rates of coastal erosion* | *Damage to key infrastructure, homes, and culturally important areas; decreased near-coastal water quality, coastal flooding and drainage issues* |
| - **Rainfall** | **Drier conditions, rather than wetter, are predicted for Puerto Rico.**  *Historical:* One analysis of weather station data from the period of 1948 to 2007 found no clear trends in total annual rainfall for the island as a whole, while another analysis showed decreases in rainfall for the island as a whole, and a third showed decreases in rainfall from -0.01 to -0.1 mm/day/year (Mendez 2010). Regionally within the island, there are indications that the southern region of Puerto Rico has experienced positive trends in annual rainfall while the western and a portion of the northern region showed decreases. Additionally, seasonal trends with observations show negative trends in summer and positive trends in winter (Mendez 2010; PRCCC 2013).  *Projections:* Model projections range from -78 to +10% (with a few models showing +30%) and current evidence suggests drier conditions are more likely than wetter for Puerto Rico, a contrast to the global precipitation signal (Neelin et al. 2006a and 2006b). The PRCCCC analysis found that past and future trends are similar, a decrease of rainfall of - 0.0012 to -0.0032 mm/day/year, projected to continue through 2050. | *Potentially more droughts and extreme events, increased runoff of sediments and pollutants* | *Loss of property, impacts to crops and livestock, issues with freshwater availability, degrading water quality along the coast; increased sedimentation that could impact reefs* |
| - **Extremes: Drought & Heavy Rain** | **It is likely that the frequency of heavy precipitation events will increase by 2050, and then decrease by the end of the century.**  *Historical:* Droughts are not very common in Puerto Rico. However, in recent decades, major prolonged droughts occurred between 1966-68, 1971-74, 1976-77, 1993-94, and 1998. In 2015, Puerto Rico experienced the third driest period since 1898 due to a particularly harsh [El Niño](https://en.wikipedia.org/wiki/El_Ni%C3%B1o). From 1958 to 2007, Puerto Rico experienced a 37% increase in very heavy precipitation (USGCRP 2009).  *Projected:* Regional downpours, defined as intense precipitation at sub daily (often sub hourly) timescales, are likely to increase in frequency and intensity in a warmer climate due to the greater capacity of warmer air to hold water vapor. Puerto Rico climate projections for the future show a probable increase in regional downpours, particularly downpour events in May through 2050. However, McSweeney et al. (2010) project a decrease in heavy rainfall events by the end of the century. | *Potentially more droughts and extreme events, increased runoff of sediments and pollutants* | *Loss of property, impacts to crops and livestock, issues with freshwater availability, degrading water quality along the coast* |
| - **Ocean pH** | **Ocean acidity has increased and is projected to continue to increase.**  *Historical:*  Currently Ωarg typically ranges from 4 to 3.7 in tropical oceanic surface waters of the Caribbean and is believed to be adequate to support robust calcification. However, these values are declining within the Caribbean region at a rate of about 3% per decade. Maximum values are in early spring and winter. During the summer and fall these values considerably decrease due to the “local effects” (PRCCC 2013).  *Projections:* It is unknown if future saturate state scenarios will continue to decline at the current rate of about 3% per decade in the Caribbean (PRCCC 2013). | *Less available carbonate to form skeletons (coral) and shells (shellfish, diatoms)* | *Potential slower growth of coral skeletons and shellfish; impacts to the habitat of reef fish; potentially weaker shells/skeletons of marine plankton and shellfish* |
| **Ocean circulation** | *No consensus, but ENSO will continue to be a source of consistent climate variability* | *Potential changes in circulation, sea level, rain patterns, drought, extreme rain events, king tides* | *Changes in freshwater availability; changes in connectivity and larval movement for corals, fish, etc.* |
| - **Hurricanes/Severe storms** | **Puerto Rico will experience a decrease in tropical cyclone frequency, but an increase in the frequency of the most intense events.**  *Historical:* Hurricanes reaching Puerto Rico gradually declined from 1730 to 2005 (Nyberg et al. 2007), where “reaching” means hurricanes that have come close enough to cause a turbidity signal in coral growth (e.g., heavy run-off and strong currents). There have been on average 3 to 3.5 major hurricanes per year from 1730-2005 (PRCCC 2013).  *Projections:* Current global climate models are rather poor in simulating tropical cyclones, due in part to the coarse spatial resolution of these models, however  IPCC climate simulations suggests that Puerto Rico will experience a decrease in tropical cyclone frequency, but an increase in the frequency of the most intense events. There may also be an increase in the intensity of winter swells reaching Puerto Rico’s coasts (PRCCC 2013). | *Potentially more intense; possible change in tracks* | *More damage caused by severe storms to community resources* |
| **OBSERVATIONS OF PAST AND PRESENT IMPACTS: Based on historical trends, what climate events are most frequent, and which have the greatest impacts? What changes to the normal seasons is the community noticing, and what are the impacts of those changes that most concern the community? Where are these changes or hazards occurring?** (Use Historical Timeline, Community Map.)  *Growing impacts to water resources, forests, coastal communities, and marine ecology are anticipated as a result of climate change.* | | | |
| **FUTURE: Based on the community’s experience and the current situation, which of the projected climate threats and impacts most concern your community, and why?** (Use available scientific projections, the indicators of a changing climate, and observations from the past and present.) *Guánica* *is most concerned with drought, heavy rainfall events, and increasing sea-surface temperatures, as these are all likely to increase and impact the resources on which the community depends.* | | | |

|  | | | | | |
| --- | --- | --- | --- | --- | --- |
| **Target Resource:** *Coral reefs in Guánica* | | | | | **Condition and Trend Rating** |
| **Condition and Trends:** *Coral reefs in the Guánica Bay Watershed area are in fair-poor condition and declining due to unsustainable and illegal fishing, invasive species, and sediment, nutrient and contaminant efflux from human activities in the adjacent watershed. Nutrient and contaminant sources include wastewater, fertilizers, stormwater runoff, atmospheric deposition, and legacy contaminants (CWP 2008). Land-based and sediment sources to coral reefs include runoff from agriculture on both steep slopes and the Lajas Valley Agricultural District, historic irrigation infrastructure in stream channels, cleared riparian areas, and draining of the historic Guánica Lagoon (CWP 2008). Overfishing and lack of enforcement are also contributing to the coral reef decline (Jackson et al. 2001; Garcia-Sais et al. 2008;* *Bradley et al. 2014).* | | | | | ***Fair-Poor ↓*** |
| **Climate Threats** | **EXPOSURE** | **SENSITIVITY** | **POTENTIAL IMPACT**  **(Exposure + Sensitivity)** | **ADAPTIVE CAPACITY** | **VULNERABILITY**  **(Potential Impact + Adaptive Capacity)** |
| *Increased sea-surface temperature resulting in increased incidence of coral bleaching*  *Ocean acidification resulting in declining coral calcification*  *Increased incidence of drought combined with heavy rainfall events resulting in increased soil erosion from existing and new non-vegetated areas* | *Very little protection from high degrees of exposure to a number of climate threats, particularly increased runoff, sea-surface temperature, and ocean acidification* | *Coral reefs have medium sensitivity to increased sea-surface temperature due to climate change. A massive bleaching event in 2005 had a dramatic impact on Puerto Rican coral reefs (Garcia-Sais et al. 2006, 2008).* | *Medium-high potential impact - Coral reefs are highly exposed to climate threats and moderately-highly sensitive to these threats.* | *Medium-low adaptive capacity – coral reefs exposed to the 2005 bleaching event have not recovered.*  *Improved watershed management is needed to reduce land-based pollution* | *Coral reef vulnerability to climate change is rated as high.* |
|  | **Exposure Rating** | **Sensitivity Rating** | **Potential Impact Rating** | **Adaptive Capacity Rating** | **Vulnerability Rating** |
|  | ***High*** | ***Medium*** | ***Medium/HIGH*** | ***Medium/LOW*** | ***HIGH*** |
|  | **VulnerabilIty statement:**   - **Condition and Trends:** *Coral reefs in the nearshore waters of the community are in fair-poor condition but are becoming degraded by sedimentation and eutrophication from land-based pollution and unsustainable and illegal fishing* - ***Vulnerability:*** *Coral reefs are vulnerable to increased sea-surface temperature, ocean acidification, and increased sediment runoff from severe storms.* - **Resource Dependency:** *Guánica coral reefs provide jobs and support a thriving tourism industry in the area.* | | | | |

References for vulnerability assessment:

Bradley P, Santavy DL and Gerritsen J. 2014. Workshop on Biological Integrity of Coral Reefs, August 21-22, 2012, Caribbean Coral Reef Institute, Isla Magueyes, La Parguera, Puerto Rico. US Environmental Protection Agency, Office of Research and Development, Atlantic Ecology Division, Narragansett, RI. EPA/600/R-13/350.

Center for Watershed Protection (CWP). 2008. *Guánica Bay Watershed Management Plan: A Pilot Project for Watershed Planning in Puerto Rico*. Ellicott City, MD.

García-Sais J, Appeldoorn R, Battista T, Bauer L, Bruckner A, Caldow C, Carrubba L, Corredor J, Diaz E, Lilyestrom C, García-Moliner G, Hernández-Delgado E, Menza C, Morell J, Pait A, Sabater J, Weil E, Williams E and Williams S. 2008. The State of Coral Reef Ecosystems of Puerto Rico. In: *The state of coral reef ecosystems of the United States and Pacific Freely Associated States: 2008*. Waddell JE and Clarke AM. (Eds). NOAA Technical Memorandum NOS NCCOS 73. 569 pp.

García-Sais JR, Castro R, Sabater-Clavell J, Esteves R and Carlo M. 2006. Monitoring of coral reef communities from natural reserves in Puerto Rico, 2006: Isla Desecheo, Rincon, Mayagüez Bay, Guánica, Ponce and Isla Caja de Muerto. Final Report submitted to the Department of Natural and Environmental Resources of Puerto Rico. San Juan, PR. 151 pp.

Gonzalez JE and Comarazamy DE. 2009. *Climate Impacts of LCLU Changes on a Tropical Coastal Region Under a Changing Climate*. 2nd International Conference on Countermeasures to Urban Heat Islands. Environmental Energy Technologies Department, E.O. Lawrence Berkeley National Laboratory, Berkeley, CA.

Hoegh-Guldberg O. 1999. Climate change, coral bleaching and the future of the world’s coral reefs. *Marine and Freshwater Research* **50**: 839-866.

Jackson JBC, Kirby MX, Berger WH, Bjorndal KA, Botsford LW, Bourque BJ, Bradbury RH, Cooke R, Erlandson J, Estes JA, Hughes TP, Kidwell S, Lange CB, Lenihan HS, Pandolfi JM, Peterson CH, Steneck RS, Tegner MJ and Warner RR. 2001. Historical overfishing and the recent collapse of coastal ecosystems. *Science* **293**:629–637.

Jury MR. 2011. Long-Term Variability and Trends in the Caribbean Sea. *International Journal of Oceanography* **2011**: 9.

McSweeney C, New M, Lizcano G and Lu X. 2010. The UNDP Climate Change Country Profiles: improving the accessibility of observed and projected climate information for studies of climate change in developing countries. *Bulletin of the American Meteorological Society* **91**: 157-166.

Mendez P. 2010. *Análisis de tendencias hidroclimáticas recientes y transformación del paisaje en la isla de Puerto Rico*. Doctoral dissertation. Universidad de Salamanca, Salamanca, España.

Neelin JD, Münnich M, Su H, Meyerson JE and Holloway CE. 2006b. Tropical drying trends in global warming models and observations. *Proceedings of the National Academy of Sciences* **103**: 6110-6115.

Nyberg J, Malmgren B, Winter A, Jury MR, Kilbourne H and Quinn T. 2007. Low Atlantic hurricane activity in the 1970s and 1980s compared to the past 270 years. *Nature* **447**: 698-701.

U.S. Global Climate Change Research Program (USGCRP). 2009. *Global Climate Change Impacts in the United States*. TR Karl, JM Melillo, and TC Peterson (eds.). Cambridge University Press, 2009.

Velazquez-Lozada A, Gonzalez JE and Winter A. 2006. Urban heat island effect analysis in San Juan Puerto Rico. *Atmospheric Environment* **40**: 1731-1741.
