# Supplementary material for: Incorporating adaptation and resilience into an integrated watershed and coral reef management plan
Source: PLoS One. 2021 Jun 24;16(6):e0253343. doi: 10.1371/journal.pone.0253343 (PMC8224911; doi:10.1371/journal.pone.0253343)
Supplement: S2 Table — Institutional affiliations are those at time of panel meetings (summer 2017). (DOCX) [file pone.0253343.s003.docx]

**Table S2. The experts who participated in the second phase panels.** Institutional affiliations are those at time of panel meetings (summer 2017).

| Dirt road management panel | Coral reef restoration panel |
| --- | --- |
| Anne Kitchell (Horsley Witten Group)   - Senior watershed planner; currently supporting NOAA through contract to provide technical support services for watershed and stormwater work | Lisamarie Carrubba (NOAA Fisheries)   - Ecologist, fisheries scientist, currently working with Caribbean endangered marine species. |
| Carlos Ramos-Scharrón (University of Texas-Austin)   - Studies land-based runoff and sediment production in tropical settings. - Has worked extensively in Puerto Rico and U.S. Virgin Islands | Paul Sturm (Director, Ridge to Reefs, Inc.)   - 20+ years of experience in watershed management - Wrote the 2008 Guánica Bay Watershed Management Plan |
| Jeiger Medina Muniz (Protectores de Cuencas)   - Involved in watershed management and restoration projects, including hydroseeding and runoff diversion to reduce sedimentation | Chris Jeffrey (NOAA National Ocean Service)   - Marine biologist/spatial ecologist with focus on the relationship between habitat destruction and exploitation on reef fish populations, and developing successful management policies that will promote sustainable use coral reef fishery resources |
| Lisa Vandiver (NOAA Restoration Center)   - Habitat restoration specialist, with focus on coastal ecology, watershed management, water quality restoration | David Vaughan (President, Plant A Million Corals, LLC/ Mote Marine Lab) Coral restoration, developer of micro-fragmentation methodExperienced in establishing and maintaining the Mote Marine land-based coral nursery |
| Rob Ferguson (CSS employee on contract with NOAA)   - Coral reef watershed management specialist and land-based sources of pollution impacts research coordinator | Sean Griffin (NOAA Restoration)   - Involved in ridge-to-reef watershed management planning for US Caribbean - Developed and oversees in-situ coral nurseries and outplanting efforts in the U.S. Caribbean |
| Roberto Viqueira Rios (Executive Director, Protectores de Cuencas)   - Watershed Restoration Coordinator for the Guánica Bay Watershed in Puerto Rico and has worked with NOAA to successfully develop a Watershed Management Plan to identify and prioritize the region's watershed restoration needs - Work has spanned across eight watersheds in Puerto Rico | Simon Pittman (NOAA National Centers for Coastal Ocean Sciences)   - Coral reef ecology and seascape ecology, spatial structure of biodiversity patterns |
