# Supplementary material for: Incorporating adaptation and resilience into an integrated watershed and coral reef management plan
Source: PLoS One. 2021 Jun 24;16(6):e0253343. doi: 10.1371/journal.pone.0253343 (PMC8224911; doi:10.1371/journal.pone.0253343)
Supplement: S3 Table — (DOCX) [file pone.0253343.s004.docx]

**S3 Table. First phase output for Adaptation Design Tool Worksheets 1A (effect of climate change on stressors) and 1B (climate change effects on the management action and implications for climate-smart design) for 12 management actions for the Guánica Bay watershed and associated coral reefs**

| **A1** | **A2** | **A3** | **A4** | **A5** | **A6** | **A7** |
| --- | --- | --- | --- | --- | --- | --- |
| **Action number** | **Existing management action** | **Target stressor(s)** | **Climate change effects on stressor(s): direction, magnitude, mechanism, uncertainty** | **Timing of climate change effects** | **Implications for effectiveness metrics and how to measure them** | **Notes** |
| 1 | Plant cover crops in Guánica Valley farms | - Terrestrial sediment and nutrients | - Storms may become more intense, leading to precipitation events with more runoff carrying sediment and nutrients from land (SWCS 2003). The percent increases in erosion and runoff will likely be greater than the percent increase in precipitation (SWCS 2003; Nearing et al. 2004). High magnitude, low uncertainty. - Stormwater plumes may extend further into the ocean, impacting more coral reefs. High magnitude, low uncertainty. - Sediment and nutrient runoff may be exacerbated by warmer air temperatures that are expected to render soils more erosion-prone (Farrell 2014). Medium magnitude, low uncertainty. - Storms of sufficient intensity to release levels of sediment and nutrients exceeding reefs’ tolerance may occur more frequently. High magnitude, medium uncertainty. - Sediment and nutrient delivery may become more intermittent due to changing precipitation patterns. Medium magnitude, medium uncertainty. | - Increasingly violent storms are already occurring. - Mudslides have been occurring on steep slopes with greater frequency. - Storm intensity will likely continue to increase over the coming decades. | **Effectiveness metrics:** Targeted percent reductions of sediment and nutrient (N and P) loads originating from farms.  **Implications for effectiveness metrics:** In addition to reducing monthly average loads, loads following storms will need to be reduced by a larger percentage to keep LBSP from crossing reefs’ thresholds. Because reefs’ LBSP thresholds may be crossed more often due to increased storm intensities, the impact of LBSP from individual storms may increase relative to the impact of chronic LBSP loads.  **Implications for how to measure effectiveness metrics:** Water quality monitoring stations should be located down-channel of farms with cover crops and those without (for comparison). It will become more important to have long-term sampling that reflects extreme storms (including throughout storms). Sampling will likely need to record a broader range of LBSP loads. | - Sediment loads may need to be minimized over a multi-day timescale, while nutrients may need to be minimized over a multi-week timescale. - How much are 2-, 5-, 10-, and 25-year storms expected to change by 2050? - What is the total runoff reduction target for reefs? - How much of a reduction in runoff from use of cover crops is necessary to reduce runoff to levels that reefs can tolerate in conjunction with other management measures? - How do cover crops in the Guánica Valley interact with hydrologically connected measures to reduce LBSP? - Downscaled climate models for Puerto Rico are at: <http://caribbeanlcc.org/interactive-map/> |
| 2 | Plant riparian buffers along the Rio Loco where it passes through farms | - Terrestrial sediment and nutrients | - Storms may become more intense, leading to precipitation events with more runoff carrying sediment and nutrients from land (SWCS 2003), especially on high slope farms along the Rio Loco. The percent increases in erosion and runoff will likely be greater than the percent increase in precipitation (SWCS 2003; Nearing et al. 2004). High magnitude, low uncertainty. - Stormwater plumes may extend further into the ocean, impacting more coral reefs. High magnitude, low uncertainty. - Sediment and nutrient runoff may be exacerbated by warmer air temperatures that are expected to render soils more erosion-prone (Farrell 2014). Medium magnitude, low uncertainty. - Storms of sufficient intensity to release levels of sediment and nutrients exceeding reefs’ tolerance may occur more frequently. High magnitude, medium uncertainty. - Sediment and nutrient delivery may become more intermittent due to changing precipitation patterns. Medium magnitude, medium uncertainty. | - Increasingly violent storms are already occurring - Mudslides have been occurring on steep slopes with greater frequency. - Storm intensity will likely continue to increase over the coming decades. | **Effectiveness metrics:** Targeted percent reductions of sediment and nutrient (N and P) loads originating from farms.  **Implications for effectiveness metrics:** In addition to reducing monthly average loads, loads following storms will need to be reduced by a larger percentage to keep LBSP from crossing reefs’ thresholds. Because reefs’ LBSP thresholds may be crossed more often due to increased storm intensities, the impact of LBSP from individual storms may increase relative to the impact of chronic LBSP loads.  **Implications for how to measure effectiveness metrics:** Water quality monitoring stations should be located down-channel of farms with cover crops and those without (for comparison). It will become more important to have long-term sampling that reflects extreme storms (including throughout storms). Sampling will likely need to record a broader range of LBSP loads. | - Sediment loads may need to be minimized over a multi-day timescale, while nutrients may need to be minimized over a multi-week timescale. - How much are 2-, 5-, 10-, and 25-year storms expected to change by 2050? - What is the total runoff reduction target for reefs? - How much of a reduction in runoff from use of riparian buffers is necessary to reduce runoff to levels that reefs can tolerate in conjunction with other management measures? - How does use of riparian buffers interact with hydrologically connected measures to reduce LBSP? |
| 3 | Replace sun-grown coffee with shade-grown coffee | - Terrestrial sediment and nutrients | - Storms may become more intense, leading to precipitation events with more runoff carrying sediment and nutrients from land (SWCS 2003), especially on high slope coffee farms. The percent increases in erosion and runoff will likely be greater than the percent increase in precipitation (SWCS 2003; Nearing et al. 2004). High magnitude, low uncertainty. - Stormwater plumes may extend further into the ocean, impacting more coral reefs. High magnitude, low uncertainty. - Sediment and nutrient runoff may be exacerbated by warmer air temperatures that are expected to render soils more erosion-prone (Farrell 2014). Medium magnitude, low uncertainty. - Storms of sufficient intensity to release levels of sediment and nutrients exceeding reefs’ tolerance may occur more frequently. High magnitude, medium uncertainty. - Sediment and nutrient delivery may become more intermittent due to changing precipitation patterns. Medium magnitude, medium uncertainty. | - Increasingly violent storms are already occurring - Mudslides have been occurring on steep slopes with greater frequency. - Storm intensity will likely continue to increase over the coming decades. | **Effectiveness metric:** Targeted percent reductions of sediment and nutrient (N and P) loads originating from coffee plantations.  **Implications for effectiveness metric:** In addition to reducing monthly average loads, loads following storms will need to be reduced by a larger percentage to keep LBSP from crossing reefs’ thresholds. Because reefs’ LBSP thresholds may be crossed more often due to increased storm intensities, the impact of LBSP from individual storms may increase relative to the impact of chronic LBSP loads.  **Implications for how to measure effectiveness metrics:** Water quality monitoring stations should be located downstream of shade-grown coffee and sun-grown coffee (for comparison). It will become more important to have long-term sampling that reflects extreme storms (including throughout the hydrograph). Sampling will likely need to record a broader range of LBSP loads. | - Sediment loads may need to be minimized over a multi-day timescale, while nutrients may need to be minimized over a multi-week timescale. - What is the total runoff reduction target for reefs? - How much of a reduction in runoff from use of shade-grown coffee is necessary to reduce runoff to levels that reefs can tolerate in conjunction with other management measures? - How does use of shade-grown coffee interact with hydrologically connected measures to reduce LBSP? - Does shade-grown coffee help with erosion reduction for storms across all ranges of intensity? - Shade-grown coffee is relatively well researched. There should be extensive literature on how to deploy it in Latin America, if not Puerto Rico specifically. - Shade-grown coffee has marketing benefits, so even though it is less space-efficient, that may be partially compensated for by its higher sale cost. Paul Sturm and others have had a roundtable to develop branding and promotion of local shade-grown coffee—can build off this. |
| 4 | Hydroseed bare soils associated with roads and homes | - Terrestrial sediment and nutrients | - Storms may become more intense, leading to precipitation events with more runoff carrying sediment and nutrients from land (SWCS 2003). The percent increases in erosion and runoff will likely be greater than the percent increase in precipitation (SWCS 2003; Nearing et al. 2004). High magnitude, low uncertainty. - Stormwater plumes may extend further into the ocean, impacting more coral reefs. High magnitude, low uncertainty. - Sediment and nutrient runoff may be exacerbated by warmer air temperatures that are expected to render soils more erosion-prone (Farrell 2014). Medium magnitude, low uncertainty. - Storms of sufficient intensity to release levels of sediment and nutrients exceeding reefs’ tolerance may occur more frequently. High magnitude, medium uncertainty. - Sediment and nutrient delivery may become more intermittent due to changing precipitation patterns. Medium magnitude, medium uncertainty. | - Increasingly violent storms are already occurring - Mudslides have been occurring on steep slopes with greater frequency. - Storm intensity will likely continue to increase over the coming decades. | **Effectiveness metrics:** Targeted percent reductions of sediment and nutrient (N and P) loads originating from bare soils. A more proximate effectiveness metric is what percent of the hydroseeded area germinates and remains green after the next few big storms.  **Implications for effectiveness metrics:** In addition to reducing monthly average loads, loads following storms will need to be reduced by a larger percentage to keep LBSP from crossing reefs’ thresholds. Because reefs’ LBSP thresholds may be crossed more often due to increased storm intensities, the impact of LBSP from individual storms may increase relative to the impact of chronic LBSP loads.  **Implications for how to measure effectiveness metrics:** Water quality monitoring stations should be located downstream of slopes with and without hydroseeding (for comparison). It will become more important to have long-term sampling that reflects extreme storms (including throughout storms). Sampling will likely need to record a broader range of LBSP loads. It will also be important to continue to monitor the vegetation coverage of the hydroseeded areas to make sure they persist. | - Sediment loads may need to be minimized over a multi-day timescale, while nutrients may need to be minimized over a multi-week timescale. - How much are 2-, 5-, 10-, and 25-year storms expected to change by 2050? - What is the total runoff reduction target for reefs? - How much of a reduction in runoff from use of hydroseeding is necessary to reduce runoff to levels that reefs can tolerate in conjunction with other management measures? - How does hydroseeding interact with hydrologically connected measures like road stabilization to reduce LBSP? |
| 5 | Construct swales to treat urban stormwater (as a type of green infrastructure) | - Urban stormwater with sediment, nutrients, and chemicals (oils, PAHs, etc.) | - Storms may become more intense, leading to precipitation events with more runoff carrying sediment, nutrients, and chemicals (SWCS 2003). The percent increases in runoff will likely be greater than the percent increase in precipitation (SWCS 2003; Nearing et al. 2004). High magnitude, low uncertainty. - Storms of sufficient intensity to release levels of sediment, nutrients, and chemicals exceeding reefs’ tolerance may occur more frequently. High magnitude, medium uncertainty. - Stormwater delivery may become more intermittent due to changing precipitation patterns. Medium magnitude, medium uncertainty. - More intense storms may erode soils with higher concentrations of legacy contaminants. Medium magnitude, high uncertainty. | - Increasingly violent storms are already occurring. - Storm intensity will likely continue to increase over the coming decades. | **Effectiveness metrics:** Targeted percent reductions of sediment, nutrients (N and P), and chemicals released from urban stormwater.  **Implications for effectiveness metrics:** In addition to reducing monthly average loads, loads following storms will need to be reduced by a larger percentage to keep LBSP from crossing reefs’ thresholds. Because reefs’ LBSP thresholds may be crossed more often due to increased storm intensities, the impact of LBSP from individual storms may increase relative to the impact of chronic LBSP loads.  **Implications for how to measure effectiveness metrics:** It will become more important to have long-term sampling that reflects extreme storms (including throughout storms). Capturing peak flow will become more important because peak flow may be important for distinguishing altered hydrology. Sampling will likely need to record a broader range of LBSP loads. | - Sediment loads may need to be minimized over a multi-day timescale, while nutrients may need to be minimized over a multi-week timescale. - How much are 2-, 5-, 10-, and 25-year storms expected to change by 2050? - What is the total runoff reduction target for reefs? - How much of a reduction in runoff from use of urban swales is necessary to reduce runoff to levels that reefs can tolerate in conjunction with other management measures? - How do swales in Guánica and other urban areas interact with hydrologically connected measures to reduce LBSP? - How much will sea level rise by 2050 and what will the storm surges be? How far into Guánica will they reach? |
| 6 | Restore Guánica Lagoon | - Terrestrial sediment and nutrients | - Storms may become more intense, leading to precipitation events with more runoff carrying sediment and nutrients from land (SWCS 2003). The percent increases in erosion and runoff will likely be greater than the percent increase in precipitation (SWCS 2003; Nearing et al. 2004). High magnitude, low uncertainty. - Stormwater plumes may extend further into the ocean, impacting more coral reefs. High magnitude, low uncertainty. - Sediment and nutrient runoff may be exacerbated by warmer air temperatures that are expected to render soils more erosion-prone (Farrell 2014). Medium magnitude, low uncertainty. - Storms of sufficient intensity to release levels of sediment and nutrients that exceed reefs’ tolerance may occur more frequently. High magnitude, medium uncertainty. - Sediment and nutrient delivery may become more intermittent due to changing precipitation patterns. Medium magnitude, medium uncertainty. | - Increasingly violent storms are already occurring. - Mudslides have been occurring on steep slopes with greater frequency. - Storm intensity will likely continue to increase over the coming decades. | **Effectiveness metrics:** Targeted percent reductions of sediment and nutrient (N and P) loads leaving the lagoon’s catchment.  **Implications for effectiveness metrics:** Ensuring that loads associated with individual storms do not exceed a designated threshold for reefs may be more important than weekly or monthly loads. Sediment and nutrients may have different timeframes for those thresholds (e.g., sediment may have to be minimized over days while nutrients over weeks).  **Implications for how to measure effectiveness metrics:** Water quality monitoring stations should be located upstream and downstream of the lagoon. It will become more important to have long-term sampling that reflects extreme storms (including throughout storms). Sampling will likely need to record a broader range of LBSP loads. | - Lagoon restoration must fully calm fears about saltwater getting into the irrigation water supply on nearby farm/pastureland; reports already written about this. - There are major political barriers to executing this plan, but it could have very significant impacts on sediment/nutrient release to reefs. - Land acquisition needs to be fully resolved. - Ability of the lagoon to detain sediment and nutrients during large storms and not overflow/dump into river is important. - Guánica Lagoon currently serves as detention storage during floods. Restoration of the lagoon that includes refilling it will reduce the capacity of the lagoon for flood storage (GME 1999). |
| 7 | Use flow diversion structures and flow reduction practices (e.g., water bars, vetiver and rock check dams, culverts) to manage sediment from coffee plantation dirt roads | - Terrestrial sediment | - Storms may become more intense, leading to precipitation events that may erode dirt roads faster, particularly on steep slopes. The percent increases in erosion and runoff will likely be greater than the percent increase in precipitation (SWCS 2003; Nearing et al. 2004). High magnitude, low uncertainty. - The rainfall threshold for erosion of dirt roads appears to be around 0.1 cm, with dirt released within 1-2 minutes (Ramos-Scharrón and Thomaz 2016). This threshold may be reached during a higher proportion of storms. High magnitude, low uncertainty. - Stormwater plumes may extend further into the ocean, impacting more coral reefs. High magnitude, low uncertainty. - Storms of sufficient intensity to release levels of sediment exceeding reefs’ tolerance may occur more frequently. High magnitude, medium uncertainty. - Road erosion may become more intermittent due to changing precipitation patterns. Medium magnitude, medium uncertainty. | - Increasingly violent storms are already occurring. - Storm intensity will likely continue to increase over the coming decades. | **Effectiveness metrics:** Targeted percent reductions of sediment loads originating from dirt roads in coffee plantations. Reduction in number of roads that need rebuilding or dramatic management after storms.  **Implications for effectiveness metrics:** In addition to reducing monthly average loads, loads following storms will need to be reduced by a larger percentage to keep sediment from crossing reefs’ thresholds. Because reefs’ sediment thresholds may be crossed more often due to increased storm intensities, the impact of acute sediment loads may increase relative to the impact of chronic sediment loads.  **Implications for how to measure effectiveness metrics:** Water quality monitoring stations should be located down-channel of dirt roads with and without altered water management strategies (for comparison). It will become more important to have long-term sampling that reflects extreme storms (including throughout storms). Ideally, water flow around roads would be watched during some storms to see how the management practices are working. Sampling will likely need to be able to record a broader range of sediment loads. | - “Expanding Conservation/Sediment Control Practices in Priority Form Areas of the Guánica Bay Watershed” (Protectores de Cuencas 2016) has extensive background on this. Says that regrading roads must be performed before other management practices are implemented. - These structures and practices are considered together because they must be implemented in combination in order to be effective (Protectores de Cuencas 2016). - How much are 2-, 5-, 10-, and 25-year storms expected to change by 2050? - What is the total sediment runoff reduction target for reefs? - How much of a reduction in runoff from use of road maintenance practices is necessary to reduce runoff to levels that reefs can tolerate in conjunction with other management measures? - How do road maintenance measures interact with other mountain erosion measures (e.g., shade-grown coffee) to reduce sediment? - Roads really need to be monitored during storms to detect locations and timing of most severe sediment runoff. |
| 8 | Construct wetlands for tertiary treatment at the Guánica WWTP | - Terrestrial nutrients | Climate change is not likely to affect nutrient input to the wetland because:   - What and how much effluent is released from secondary treatment and enters the wetlands is human-controlled, and; - The catchment for the constructed wetlands is small and runoff to the wetlands is minimal (Paul Sturm, pers. comm). | - Climate change is already acting on nutrient delivery to reefs. | **Effectiveness metric:** Nutrient loads leaving the tertiary treatment wetlands.  **Implications for effectiveness metric:** Ensuring that loads associated with individual storms do not exceed a designated threshold for reefs may be more important than weekly or monthly loads  **Implications for how to measure effectiveness metrics:** It will become more important to have long-term sampling that reflects extreme storms (including throughout storms). Sampling will likely need to record a broader range of LBSP loads. Also, important to record any times that the wetlands’ berms are breached by storm surge, particularly as sea level rises. | - Is there any way in which human behavior affects nutrient input to the constructed wetlands from secondary treatment? |
| 9 | Protect seagrass meadows | - Terrestrial sediment and nutrients (seagrass filters runoff) - Coastal and near-shore erosion and sediment release (seagrass protects shoreline and binds sediment) - Reduced inter-habitat connectivity (seagrass provides nursery for young fish stock and hunting grounds for adults) | - Storms may become more intense, leading to precipitation events with more runoff carrying sediment and nutrients from land (SWCS 2003). The percent increases in erosion and runoff will likely be greater than the percent increase in precipitation (SWCS 2003; Nearing et al. 2004). High magnitude, low uncertainty. - Stormwater plumes may extend further into the ocean, impacting more coral reefs. High magnitude, low uncertainty. - Coastal erosion and near-shore sediment resuspension may increase if storms become stronger (2013 Baseline Assessment suggests that resuspension is common). Medium magnitude, low uncertainty. - Ocean acidification could alter fishes’ olfactory abilities, which could reduce inter-habitat connectivity by reducing larvae’s ability to find seagrass nurseries (Munday et al. 2009- PNAS, Munday et al. 2012- Marine Biology, Munday et al. 2014- Nature Climate Change), juvenile fishes’ ability to find the coral reef once they’re ready to move out of the seagrass, or adult fishes’ ability to nocturnally forage in seagrass (Devine et al. 2012- Global Change Ecology, Devine and Munday 2013- Marine Biology). Unknown magnitude, medium uncertainty. | - Increasingly violent storms are already occurring. - Mudslides have been occurring on steep slopes with greater frequency. - Storm intensity will likely continue to increase over the coming decades, with resuspension increasingly commensurately. | **Effectiveness metrics:** Acreage of seagrass within an ecologically meaningful distance of reefs. Reduction in loads of sediment and nutrients reaching coral areas. Species richness and diversity of juvenile (for use as reef nursery) and adult (for use as hunting ground) reef fish in seagrass.  **Implications for effectiveness metric:** Seagrass acreage may fluctuate more wildly. Some areas historically suitable for seagrass may no longer be so, while other areas may now be suitable. Seagrass species composition may change (Fourqurean et al. 1995, Frankovitch and Fourqurean 1997). In addition to reducing monthly average loads, loads following storms will need to be reduced by a larger percentage to keep LBSP from crossing reefs’ thresholds. Because reefs’ LBSP thresholds may be crossed more often due to increased storm intensities, the impact of LBSP from individual storms may increase relative to the impact of chronic LBSP loads.  **Implications for how to measure effectiveness metrics:** Longer term and more spatially extensive seagrass monitoring will be necessary to capture potentially larger shifts in acreage. Only certain substrate is habitable by seagrass (2013 Baseline Assessment), so that area must be measured. More monitoring after storms will be needed to measure sediment and nutrient loads reaching reefs. Instruments with a wider measurement range may be needed. | - Which seagrass beds are most important to protect? - What is the relative importance of the following contributions of seagrass to coral reefs: nutrient/sediment reduction/baffling, young fish nursery, adult hunting ground? - Will climate change affect ability of reef fish to hunt in seagrass or to successfully complete ontogenetic habitat changes? - NOAA has daytime fish survey data from seagrass. |
| 10 | Protect mangrove forests | - Terrestrial sediment and nutrients (mangroves filter runoff) - Coastal erosion (mangroves stabilize shoreline) - Reduced inter-habitat connectivity (mangroves provide nursery for young fish) | - Storms may become more intense, leading to precipitation events with more runoff carrying sediment and nutrients from land (SWCS 2003). The percent increases in erosion and runoff will likely be greater than the percent increase in precipitation (SWCS 2003; Nearing et al. 2004). High magnitude, low uncertainty. - Stormwater plumes may extend further into the ocean, impacting more coral reefs. High magnitude, low uncertainty. - Coastal erosion may increase if storms become stronger (2013 Baseline Assessment suggests that resuspension is common). Medium magnitude, low uncertainty. - Ocean acidification could alter fishes’ olfactory abilities, which could reduce inter-habitat connectivity by reducing either larvae’s ability to find a mangrove nursery or juvenile fishes’ ability to find the coral reef once they’re ready to move out of the mangroves (Munday et al. 2009- PNAS, Munday et al. 2012- Marine Biology, Munday et al. 2014- Nature Climate Change). Unknown magnitude, high uncertainty. | - Increasingly violent storms are already occurring. - Mudslides have been occurring on steep slopes with greater frequency. - Storm intensity will likely continue to increase over the coming decades. | **Effectiveness metric:** Kilometers of mangrove forest within an ecologically meaning distance of reefs. Reduction in loads of sediment and nutrients arriving at the reef. Species richness and diversity of juvenile reef fish in mangroves (for use as reef nursery).  **Implications for effectiveness metric:** Some areas historically suitable for mangroves may no longer be so, while other areas may become suitable. In addition to reducing monthly average loads, loads following storms will need to be reduced by a larger percentage to keep LBSP from crossing reefs’ thresholds. Because reefs’ LBSP thresholds may be crossed more often due to increased storm intensities, the impact of LBSP from individual storms may increase relative to the impact of chronic LBSP loads.  **Implications for how to measure effectiveness metrics:** More monitoring after storms will be needed to measure sediment and nutrient loads reaching reefs. Instruments with a wider measurement range may be needed. | - Which mangrove forests are most important to protect? - What is the relative importance of mangroves reducing nutrients and sediment loads to reefs and serving as nurseries for young reef animals? - USGS and Eckerd College found that in the USVI mangrove roots act as a refuge for corals. In 2015, exposed colonies bleached more frequently than did colonies amidst the mangrove roots. - To what extent will climate change affect ability of reef fish to successfully complete ontogenetic habitat shifts? - NOAA has fish survey data from mangroves. |
| 11 | Capture larval fish of target species and establish reef fish aquarium-based nurseries | - Fishing pressure - Reduced fish recruitment | - Water temperature and chemistry changes could alter fish recruitment to reefs due to range shifts or increased mortality (Pankhurst and Munday 2011- Marine and Freshwater Research, Munday et al. 2011- Coral Reefs, Munday et al. 2012- PNAS). High magnitude, high uncertainty. - Fishing pressure around Guánica may increase as land-based livelihoods (e.g., farming) become more challenging (Daw et al. 2009). Medium magnitude, high uncertainty. - Outbreaks of fish diseases may increase with warmer water. Low magnitude, medium uncertainty. - More intense storms may reduce available fishing days, concentrating fishing into a smaller number of days per year, which may intersect differently with fish spawning and recruitment periods or locations. Medium magnitude, high uncertainty. - More intense storms may impair fishing infrastructure, reducing fishing pressure. Low magnitude, high uncertainty. | - No readily available information on whether people are relying more on fishing now because their old profession had become too unreliable due to climate change. | **Effectiveness metric:** Number of individuals and species in nurseries, survival in nurseries.  **Implications for effectiveness metric:** None are evident.  **Implications for how to measure effectiveness metrics:** None are evident. | - This action assumes that there is habitat for reef fish to live on. If there isn’t a living reef, or at least a reef structure, coral-dependent fish species may not survive once released. - How can managers tell if people are increasing fishing effort because other livelihoods are becoming less reliable? Puerto Rico Sea Grant does periodic surveys on fishing socioeconomics. - Or could fishing decrease due to climate change because it is viewed as not rewarding enough and too unreliable? Caribbean experience suggests that reefs undergo much more intensive fishing pressure than Guánica has been (e.g., Jamaica, see Hawkins and Roberts 2004). - Ecocean website: <http://www.ecocean.fr/en/> - Biological condition gradient (BCG) fish community rules are one way to measure success of fish releases. - Recreational fishing pressure is increasing, and artisanal fishing pressure is decreasing around Guánica (Bradley et al. 2016). |
| 12 | Collect corals and establish aquarium-based coral nurseries | Coral loss from:   - Warmer ocean water - Lower pH ocean water - Terrestrial sediment and nutrients - Sea level rise | - Warmer waters may increase bleaching episodes and disease outbreaks. High magnitude, low uncertainty. - Storms may become more intense, leading to precipitation events with more runoff carrying sediment and nutrients from land (SWCS 2003). The percent increases in erosion and runoff will likely be greater than the percent increase in precipitation (SWCS 2003; Nearing et al. 2004). High magnitude, low uncertainty. - Stormwater plumes may extend further into the ocean, impacting more coral reefs. High magnitude, low uncertainty. - Sediment and nutrient runoff may be exacerbated by warmer air temperatures that are expected to render soils more erosion-prone (Farrell 2014). Medium magnitude, low uncertainty. - Storms of sufficient intensity to release levels of sediment and nutrients exceeding reefs’ tolerance may occur more frequently. High magnitude, medium uncertainty. - Sea level rise may occur faster than reef accretion, leading to “sinking reefs”. Medium magnitude, low uncertainty. - Ocean acidification could decrease successful recruitment if corals cannot find settlement sites (e.g., due to altered chemosensory abilities) or calcify (due to acidic conditions) (Doropoulos et al. 2012- Ecology Letters, Webster et al. 2012- Global Change Biology). It may also reduce existing colonies’ growth rates. High magnitude, medium-high uncertainty. - Sediment and nutrient delivery may become more intermittent due to changing precipitation patterns. Medium magnitude, medium uncertainty. | - Temperature effects have already occurred, with increasing magnitude through mid-century. - Timing of acidification is unknown. - Increasingly violent storms are already occurring. - Storm intensity will likely continue to increase over the coming decades. | **Effectiveness metrics:** Number of colonies in nurseries, number of climate-tolerant genotypes in nurseries, and colony survival in nurseries.  **Implications for effectiveness metrics:** None are evident.  **Implications for how to measure effectiveness metrics:** None are evident. | - What coral species are best to use? - Dave Vaughan (Mote Marine Lab) is reproducing a variety of coral species by fragmentation in lab aquaria. |

| **B1** | **B2** | **B3** | **B4** | **B5** | **B6** | **B7** | **B8** |
| --- | --- | --- | --- | --- | --- | --- | --- |
| **Action number** | **Existing management action** | **Changes in effectiveness of management action due to: climate impacts on target stressor** | **Changes in effectiveness of management action due to: climate impacts on management action** | **Time frame or constraint for using the action and implementation (e.g., urgency, longer or shorter term)** | **What changes are needed to adapt the action (place, time, and engineering design)** | **Climate-Smart Management Action** | **Notes** |
| 1 | Plant cover crops in Guánica Valley farms | - Cover crops may become less effective at retaining sediment and nutrients due to more intense precipitation events (greater 30-minute intensity maximum; Revised Universal Soil Loss Equation- RUSLE) and more rain overall. - Soil may become more erosion-prone if it becomes drier and more compacted (smaller soil pores leading to less infiltration and more surface flow). | - Cover crops may be uprooted by more powerful surface flows from stronger storms, especially in steeper sloped areas (valley sides). - Cover crops may not be sufficiently drought- and heat-resistant to survive and retain soils made drier by warmer air and more intermittent precipitation. - Large storms between cash crop harvesting and cover crop planting could cause ponding in poor drainage areas, making conditions unfavorable for cover crops. | - This can be implemented immediately and should be re-implemented after each harvest. - It has the potential to quickly affect sediment and nutrient loads. - Timing of planting may need to be altered so that cover crops provide maximal soil coverage during the strongest storms. - Timing of cover crop termination may need to be shifted due to altered soil moisture. | - Cover crops should be selected for both drought and high-flow resistance. It might be possible to plant different cover crops depending on the forecast for the fallow period (wet versus dry). - Prioritize fields near irrigation channels and on steeper slopes (where erosivity is higher). - Plant the cover crops as soon as is feasible after the previous crop has been harvested so that fields are uncovered less time. But planting time should also be cognizant of whether a large storm might come and pond water on poorly draining fields. | Annually plant a mixture of cover crops that are drought- and flood-resistant, timing them such that the cover crops will protect soil from rain during the strongest storms. Preferentially plant cover crops near irrigation channels and on more erosion-prone soils. Monitor and be prepared to replant cover crops in case of damage from extreme events. | - Which fields and in which parts of the fields is this the highest priority? - What would be good cover crops in the valley floor versus lower sides? - How can we get buy-in from farmers for this effort? - Nitrogen-fixing cover crops would be an added benefit if they reduce farmers’ needs to use fertilizers. - How much of a reduction in sediment and nutrients will e.g. 10 acres of cover crops produce? - A roundtable as was conducted for hydroseeding would gather local knowledge. |
| 2 | Plant riparian buffers along the Rio Loco where it passes through farms | - Riparian buffers may not be wide enough to slow surface runoff from stronger storms sufficiently to let sediment settle out and prevent channels through the buffer from forming. - Riparian buffers may not be wide enough to allow adequate infiltration for roots to absorb dissolved nutrients. - Runoff balance could shift between subsurface flow and surface runoff, allowing more runoff to bypass roots and reach waterbodies. - Soil may become more erosion-prone if it becomes drier and more compacted (smaller soil pores leading to less infiltration and more surface flow), producing more sediment and surface runoff. | - Riparian buffers may be eroded more easily by more powerful surface flows in stronger storms. - Riparian buffers nearest the streams/Rio Loco may be eroded more easily by larger in-stream flows. - Buffer vegetation may need to be more drought- and heat-resistant to survive and retain soils made drier by warmer air and more intermittent precipitation. - Wherever the Rio Loco or its tributaries are very deeply incised, the roots of buffer vegetation may not extend deep enough to reach streamflow or groundwater, and therefore may not hold banks together, may not intercept subsurface flow, and may die. - Grass buffers strips may need to be maintained (cleaned out) more often because of faster sediment accumulation from larger storms (http://www.soil.ncsu.edu/publications/BMPs/buffers.html) | - This can be implemented immediately. It has the potential to quickly affect sediment and nutrient loads. | - Riparian buffer plants need to be selected for both drought and high-flow resistance. They should tolerate higher temperatures and drier soils than they currently experience. - Buffer widths need to be designed to trap sediment in surface flow and allow infiltration of water with dissolved nutrients into soil during larger flows (or larger flows more frequently). - It might be best to preferentially buffer ones that have larger catchments, so that more lateral flow is intercepted. But buffer locations should also consider how water flow will change across farms and in streams due to altered precipitation patterns and soil properties. - Use vegetation that will send roots to soil depths that are expected to intercept runoff under new soil properties, air temperatures, and precipitation regimes. | Plant riparian buffers that will be an adequate width to withstand and intercept potentially larger surface and subsurface flows across farms. The buffers should be planted in locations that will intercept the maximum runoff possible (probably determined by hydrological models). Buffer plants should be able to withstand larger in-stream flows and across-farm flows, hold together drier soils, and withstand higher air temperatures with potentially longer dry periods. | - Where are the best places to plant buffers (particular farms and locations in farms)? - What would be good buffer plants at high altitudes and steep slopes? - How can we get buy-in from farmers for this effort? - How will balance of runoff shift between surface and sub-surface? - River restoration in these areas would provide a larger floodplain. - How to balance buffering larger (year-round) streams against buffering smaller (seasonal) streams? Research from temperate regions might be applicable here, in addition to tropical buffer research. - Modeled buffer-climate change interaction here: http://adsabs.harvard.edu/abs/2010AGUFMGC51I0836C |
| 3 | Replace sun-grown coffee with shade-grown coffee | - Tree canopies currently used with shade grown coffee may not be sufficient to control greater erosion expected with increased storm intensity. - Increased peak intensity of precipitation may increase erosion (Revised Universal Soil Loss Equation- RUSLE). - Soil may become more erosion-prone if it becomes drier and more compacted (smaller soil pores leading to less infiltration and more surface flow). | - Higher temperatures may be unsuitable for certain shade trees, though the downstream effects of that are unclear. Could shade trees experience higher mortality, requiring more human maintenance of the canopy? - In warmer air, the decomposition rate of shade tree litter may increase. Could that increase the overall levels of nutrients in the soil, leading to higher nutrient loadings for a rainstorm of a given size? Or could higher decomposition rates leave less leaf litter to be carried away in storms, thereby reducing nutrient loading? Very speculative but nutrient cycling may be affected. - Warmer temperatures may also shift the balance between coffee plants/shade trees and their parasites/herbivores, or between their parasites and the predators/parasitoids of their parasites. How those community interactions will be altered are very uncertain but negative outcomes could include increased herbivory/parasitism of coffee plants and their shade plants. | - This action should be enduring and employed as quickly as shade-tolerant coffee shrubs can be planted. | - Shade trees will need to intercept more rain to prevent excessive erosion. Increased rain interception could come from more densely planted shade trees or shade trees with denser foliage. However, coffee plants should not be deprived of too much rain and light, so pruning of shade trees may be necessary based on changing rain regimes. - The shade trees will need to be more tolerant of heat and possible longer dry periods. - Coffee near streams or on particularly erosion-prone soil (e.g., steeper slopes) should preferentially be converted to shade-grown. | Plant shade-tolerant coffee with heat-tolerant shade plants, with priority to more erosion-prone slopes. Plant understory/secondary canopy plants that will effectively use or provide nutrients (e.g., n-fixation if nitrogen is likely to become more limiting) and stabilize soil under potentially increased storms, perhaps informed by FWS studies. Prune shade plants as necessary to allow adequate light and rain to reach coffee plants. Implement first on more erosion-prone slopes. | - Shade-grown coffee should also affect nutrient cycling. Shade-grown coffee is demonstrated to retain more nutrients, but how would climate change affect that? And would it be a significant change? - Certain shade tree species may be better suited to the warmer air and potentially longer dry periods. - Fish & Wildlife Service (FWS) developed matrix of plants to use in shade-grown coffee communities in Puerto Rico several years ago (according to Pat Bradley). |
| 4 | Hydroseed bare soils associated with roads and homes | - Hydroseeded areas may become less effective at retaining soil due to more intense precipitation events (greater 30-minute intensity maximum; Revised Universal Soil Loss Equation- RUSLE) and more rain overall. - Soil may become more erosion-prone if it becomes drier and more compacted (smaller soil pores leading to less infiltration and more surface flow). - Altered precipitation regime may change the kind of erosion along road slopes, which may affect the appropriate species for used in hydroseeding. For example, due to altered soil properties and prior moisture content the balance between gulley runoff and slumping/mass wasting might change. | - Deployed hydroseeding mixture or germinated plants could be uprooted more easily by more powerful surface flows in stronger storms. - Hydroseeded locations may have longer dry periods, increasing the risk of seed or grass mortality which could expose more soil surface and increase the potential for erosion and gully formation. - Hydroseeded grass may grow faster due to increased CO_2_ availability, improving the speed at which it consolidates soil and shorting the time until which it becomes effective. | - This can be implemented immediately. It has the potential to quickly affect sediment and nutrient loads. | - Use plants which will be able to withstand potentially stronger storms and longer dry periods. Their root systems may need to be longer (reach further into soil) than previously necessary to keep soil in place. - The plant(s) should have high density coverage even during dry periods to keep soil top covered so that when it rains, soil is not more exposed to rain drops. - The hydroseeding mixture may need to be altered to bind soil together better and retain moisture for longer periods since dry periods may be longer. | Hydroseed bare soils that are especially erosion-prone. Use a hydroseeding mixture that will be able to effectively bind the soil during stronger storms until and after the plants germinate. The plants should be selected to protect soil during longer dry periods. Monitor slopes following large storms and be prepared to reseed more frequently. | - Hydroseeding has already occurred in some areas as part of implementing the 2008 WMP. Use those methods as a starting place? - Need to make sure to check germination performance after storms. - What mixture(s) will perform best with current rainfall and temperature predictions on steep slopes? - Fertilizer is often included in hydroseeding mixtures. Can its use be avoided here? - Use bonded fiber matrix (BFM) due to predictions of strong storms? - Which slopes are most important to protect? |
| 5 | Construct swales to treat urban stormwater (as a type of green infrastructure) | - Swales may be flooded by more intense storms producing larger runoff volumes, thereby allowing stormwater to avoid infiltration. - Water may stay in swales longer if infiltration rates are slower, keeping swale water levels higher. - Swales may fill more rapidly with sediment and debris (trash, plants, etc.) that are carried by stormwater, which will reduce the ability of the swales to store water. | - Salty Bay water may flood swales within flooding range of the coast during storms, killing freshwater plants and reducing storage volume available for urban stormwater. - Larger storms may cause swales to erode, releasing more sediment. - Longer dry periods may hurt or kill plants in swales. | - This can be implemented immediately. Swales can be re-designed for progressively larger storms. | - Swales need to be larger to handle larger storms. The extra size should not result in a steeper slope, so that soil erosion within the BMP does not increase. - Swales within likely storm surge areas of Guánica should be vegetated with salt-tolerant plants. - Swales will need to be cleaned of debris and perhaps sediment more frequently and/or more quickly after storms. - Swales will need overflow mechanisms to divert excess stormwater. | Construct swales in areas that will not likely be flooded by storm surges or use saltwater-tolerant plants in swales if they are likely to be repeatedly inundated by storm surges. Size them according to projected future storm intensities, with side slopes that are less erodible. Size them to retain greater volumes of water, for longer after storms, and handle more debris and sediment. | - What parts of Guánica and other urbanized areas will regularly be subjected to storm surges by 2025 and 2050? - What plants are best for vegetating swales? - Swales can be used in combination with rain gardens and other urban green infrastructure. - Can swales be large enough to handle runoff from the largest storms? What other BMPs should they be combined with? - Where should swales be located? |
| 6 | Restore Guánica Lagoon | - Lagoon restoration may not be sufficient to manage the greater amounts of sediment, nutrients, and debris due to stronger storms that will cause more Lajas Valley irrigation ditch erosion (more precipitation, larger peak flows.) - Drier, more compact soil could be eroded more easily, exacerbating over-loading of lagoon’s retention capacity. - Lagoon may become more effective at nutrient removal; some evidence shows that warmer water leads to faster nutrient uptake. - Maintaining preferred water depth in the lagoon could become more difficult during more intense storms. Also, longer dry periods and higher temperatures could make water levels too low. | - Warmer temperatures could harm the lagoon’s flora and fauna, reducing nutrient processing efficiency. - More rapid sediment accumulation could alter flow through the lagoon or fill it up. - Storm surge reaching further inland from larger storms on top of sea level rise could introduce salty Bay water to the lagoon and kill flora. | - Ridge to Reef has prioritized this measure. | - The lagoon needs to be able to accommodate potentially larger storms, as well as associated sediment loads. The lagoon should be designed in such a way that it retains sediment but won’t get backed up and won’t have a giant washout into Guánica Bay during larger storms. - An altered water replenishment rate due to changed precipitation patterns also needs to be considered. Insufficient lagoon water level could be problematic, though probably not as problematic as excessively high. Perhaps lagoon flora can be selected with the idea that water levels will tend to the lower side. - The lagoon and its surrounding infrastructure may need to be built further inland or have higher protective berms to protect it from larger storm surges. - The Lajas Valley drainage canal needs to be redesigned to reduce sediment delivery (separate management action). | Restore Guánica Lagoon to handle flows and associated sediment loads projected by climate models. Consider extra sediment settling or diversion mechanisms to keep the lagoon from filling up with larger sediment loads during storms, and additional sediment removal following storms. The lagoon should also be designed to handle potentially longer dry periods, during which water levels will not be replenished. The lagoon should be physically protected from storm surges by berms of adequate height. | - Not clear how rising sea levels or water table will affect lagoon’s functioning. Will it need to be further inland? - There are major political barriers to executing this plan, but it could have very significant impacts on sediment/nutrient release to reefs. - Should the canals and reservoirs be concurrently revised to reduce sediment delivery to the lagoon? Would reservoir water be released prior to storms? Their drainage pipes are generally below the sediment level, so this could be a major delivery of sediment to the lagoon. - Guánica Lagoon currently serves as detention storage during floods. Restoration of the lagoon that includes refilling it will reduce the capacity of the lagoon for flood storage, though without increasing the 100-year flood elevation beyond permitted levels (GME 1999 H&H analysis). |
| 7 | Use flow diversion structures and flow reduction practices (e.g., water bars, vetiver and rock check dams, culverts) to manage sediment from coffee plantation dirt roads | - Water diversion structures may not be able to divert all water off roads, producing downhill road erosion. - Flow and erosion reduction practices may not be able to slow down water sufficiently to prevent further erosion and trap sediment. - Vertical dirt walls along roads may be more likely to collapse under increased precipitation. - Interstices between stones in check dams or stone swales may become clogged with sediment more quickly from larger or more frequent storms. | - Larger storms may wash out existing water diversion structures (e.g., water bars or the stones comprising check dams). - Larger storms may wash out existing flow and erosion reduction practices (e.g., vetiver and rock check dams). - Larger storms may overflow culverts or wash them out entirely. - Road regrading may be complicated by increased risk of raised road banks slipping onto road during or after regrading. Slippage is already happening but could happen more under some precipitation scenarios. | - This suite of actions can be implemented immediately. They have the potential to quickly affect sediment loads. | - Compact dirt roads that have been topped with aggregated crushed stone. - Make road crown higher to consistently drain water to sides of road, if not using insloped or outsloped roads. - Implement stronger and more frequent debris barriers upstream of culverts to prevent culvert clogging. - Use larger rip-rap stones or more extensive vetiver patches on downstream sides of culverts to diffuse faster and larger flows. - Increase frequency of rolling dips or water bars on steeper-sloped roads or stretches likely to experience heavy erosion. - Be prepared to clean accumulated sediment out of interstices between sediment trap rocks more frequently. - Roads on particularly steep slopes may need to be paved (with asphalt or concrete) if other management actions are not working. | Minimize sediment from existing dirt mountain roads by building water diversions more frequently along roads, sloping roads more heavily to promote faster drainage, and augmenting barriers on upstream sides of culverts and flow diffusers on downstream sides of culverts. Locations requiring flow control may change due to altered precipitation patterns. Check integrity and repair diversion structures after larger storms; remove sediment from sediment traps after large storms. Compact roads with surfaces made of small rocks and granular material to stabilize road surfaces. Pave roads that have already repeatedly washed out if other mitigation techniques are not possible. | - These structures and practices are considered together (as a suite of actions) because they must be implemented in combination in order to be effective. - To what extent can dirt roads traversing hills and mountains be retrofitted? - Which roads is it most important to work on? - This is a feedback loop: the worse the road water management actions perform, the more erosion there will be, and the worse they will perform. |
| 8 | Construct wetlands for tertiary treatment at the Guánica WWTP | - Wetlands may become more effective at nutrient removal; some evidence shows that warmer water leads to faster nutrient uptake. - Maintaining optimal water depth (1-2 ft.) for effective treatment beds could become more difficult during more intense storms. Also, longer dry periods and higher temperatures could lower water levels below that of a functional wetland. | - Warmer water in wetlands may be injurious or lethal to the current plant community (whether it is natural or human assembled). - With sea levels expected to rise between 0.5 and 2 m by 2100, saltwater intrusion could occur, or storm surges could overtop the protective berms (2.2 m above MSL), exposing freshwater wetland plants to lethal salinities. | - The sooner the better. The wetlands are already largely designed. After implementation, some changes may need to be made later, like using a different grass species or raising the berms. | - One or more different species of grass may need to be planted to withstand warmer wetland water. - The berms around the cells may need to be taller. - Controls of inflow and outflow may need to be refined if larger storms add more water, but higher temperatures and generally reduced precipitation reduce water levels. - A bottom and side lining that is resistant to saltwater may be necessary to prevent intrusion. | Construct tertiary treatment wetlands at the GB WWTP using grass species that are tolerant of warmer water. Use linings to protect from saltwater intrusion, or else prepare for a shift to salt tolerant plants in the future. The protective berms should be tall enough to withstand storm surge from higher sea levels and potentially more powerful storms. Water level control in the cells will need to consider greater evaporation rates and larger storms. | - Is a different species or genus of grass more appropriate for warmer water? - What is the risk of saltwater intrusion from below as sea levels rise? - How much fluctuation will there be in wetland cell water levels and will it exceed the acceptable range? - Does the currently designed berm height take into account storm surge heights expected under climate change with sea level rise? - Presumably the location of the wetland cells is locked in because of land acquisition by PRASA and proximity to the primary/secondary WWTP. So, no refuge from taller surge by moving them inland. |
| 9 | Protect seagrass meadows | - Seagrass acreage may be inadequate to filter or trap enough sediment/nutrients in runoff produced by larger storms. - Seagrass acreage and density may be inadequate to sufficiently mitigate resuspension of sediments during larger storms. - Protected seagrass meadows may not be optimally located to maintain nursery and predation ground connectivity with reefs. - Seagrass acreage may be inadequate for fish nurseries. | - Warming water may kill seagrasses that are near the upper limits of their thermal tolerance. - Rising sea levels will reduce light reaching seagrasses at marginal depths and may slow their growth. - Epiphytic or benthic algae could have a competitive advantage over seagrasses due to slower seagrass growth rates. - Stronger and/or more frequent storms will increase the disturbance regime, which could kill more seagrasses. - Increased CO_2_ in the water could alter competition between species by differentially affecting growth rates of species (for nutrient example, see Fourqurean et al. 1995). The resulting shift in seagrass community composition could affect terrestrial sediment and nutrient baffling and connectivity to reefs. | - Existing seagrass meadows should be protected now and in perpetuity. This must be an ongoing effort. - Connectivity may be reduced once water’s pH falls below the ability of fishes’ ability to acclimate (Donelson et al. 2012- Nature Climate Change, Miller et al. 2012- Nature Climate Change), but timing for that is unknown. | - Seagrass meadows in shallower water should be prioritized for protection since they are less likely to be living in too deep water as sea levels rise. - Seagrass meadows near freshwater sources (e.g., within Guánica Bay or near the mouth of the Bay) should be prioritized for protection because of their prime location for filtering terrestrial input. If grasses in those locations appear to be dying, perhaps they can be replaced by more appropriate species (as identified by research). - Seagrass meadows at distances from coral reefs useful for altered nursery and feeding connectivity should have priority protection. Those distances may need to be shorter if altered water chemistry restricts the ability of fish to use multiple habitats. | Preferentially protect seagrass meadows between large freshwater inputs and priority reef tracts, especially in shallower locations.  Protect seagrass meadows that will maintain their function as nurseries and feeding grounds given potentially altered abilities of fish to complete habitat shifts (meadows nearer reefs). | - Where do seagrass communities most need to be protected to shield existing (and future) reefs from terrestrial sediment/nutrients and resuspension? Coastal flow is from east to west. - What seagrass species are most storm- and heat-resistant? - What seagrass communities are most important for reef fish nurseries and for reef fish hunting grounds? - What seagrass communities filter sediment and nutrients out of the water the best? - How can existing seagrass communities be modified to make them serve the above purposes better? - Seagrass restoration is a separate management action. |
| 10 | Protect mangrove forests | - Extent of mangroves may be inadequate to filter or trap enough terrestrial runoff produced by larger storms. - Extent of mangroves may be inadequate to prevent excessive coastal erosion. - Protected mangroves may not be optimally located to maintain nursery connectivity with reefs. - Extent of mangroves may be inadequate for fish nurseries. | - Stronger and/or more frequent storms will increase the likelihood of mangrove destruction and severe coastal erosion. - If mangrove sediment accretion rates are lower than sea level rise rates, mangrove forests retreat landward. If there is limited space for landward migration, the mangrove forest will become narrower. - More intense storms interspersed with longer dry periods will cause salinity in mangrove forests to fluctuate more, which will decrease productivity (Gilman et al. 2008). - Higher temperatures and higher CO_2_ levels could increase productivity of mangroves, though this depends on species, salinity, etc. (Gilman et al. 2008; Alongi 2015). | - Existing mangrove forests should be protected now and in perpetuity. This must be an ongoing effort. - Connectivity will be reduced once water’s pH falls below the ability of fishes’ ability to acclimate (Donelson et al. 2012- Nature Climate Change, Miller et al. 2012- Nature Climate Change), but timing for that is unknown. | - Because more sediment may be delivered from the bay, mangroves should be preferentially protected to intercept as much of that sediment as possible. - Wherever possible, there should be landward space for mangroves to migrate in case they are not able to keep up with sea level rise. Areas in which mangroves are anticipated to migrate inland and where there’s space could be planted with buttonwoods. - Any mangroves that serve as refugia for corals or coral-associated organisms should be preferentially protected. Likewise, mangroves that could serve as refugia (positioned to provide a good amount of shading) should be protected so that corals can be planted there. - Mangroves at distances from coral reefs useful for altered nursery connectivity should have priority protection. Those distances may need to be shorter if altered water chemistry restricts the ability of fish to use multiple habitats. | Protect mangroves that will be positioned to intercept as much runoff as possible from climate-altered hydrology. Also, protect mangroves that serve as climate refugia for reef organisms and that are likely to continue to be nurseries, taking into account potentially altered reef-mangrove connectivity.  Where possible, preserve landward space for mangroves to retreat into if necessary. | - Where do mangroves most need to be protected to help existing (and restored) reefs from terrestrial sediment/nutrients? - Do any mangroves nearby serve as refugia for corals by buffering pH and/or providing shading? - Does mangrove protection need to occur at the same time as seagrass protection since the habitats are linked?   NOTE: Mangrove forest restoration is not considered here. |
| 11 | Capture larval fish of target species and establish reef fish aquarium-based nurseries  (Since this action is about the “supply side” of the fish replenishment plan, this is about how climate change affects the supply of larvae for the management action and what happens in the nursery. Releasing the fish is part of a separate follow-on action, which would benefit from a resilience assessment.) | - Supply of post-larvae for the rearing program may be affected by changing water chemistry and temperature. These could alter spawning timing, location, and amount of reproductive fish (e.g., Miller et al. 2013- Global Change Biology, (Munday et al. 2009- PNAS, Munday et al. 2012- Marine Biology, Munday et al. 2014- Nature Climate Change), the effects from which may be perpetuated from pelagic development to post-larval return and capture. | - None, unless climate change effects somehow physically destroy the aquaria on shore. | - Unless this action is limited to fish that can survive in existing coral-depleted habitat, it should follow habitat reconstruction (e.g., coral outplanting). It should also probably come after fishing regulation is enforced (perhaps in conjunction with an MPA) so that fish are given time to mature and reproduce naturally. - Also, it should follow protection or restoration of mangroves and seagrasses necessary for a fully connected ecosystem. | - Assuming that there are fewer post-larval individuals of desirable fish species, it may take more effort to capture the desired numbers for rearing. That means more boat trips out at night and paying more crews. - The similarity between the water supplying the rearing aquaria and the place(s) where the fish will be released also needs to be considered. Presumably, these should be similar in temperature, pH, and chemical composition. - More post-larvae will need to be captured to sufficiently replenish target populations due to higher mortality once fish are returned to the reef. This will increase the space and equipment requirements of the nursery. | Establish reef fish aquarium-based nurseries that use water similar to where the fish will be released. Capture and rear more fish to release since mortality may be higher from multiple causes during rearing and after release. Post-larvae may be found in different places due to altered water chemistry and circulation.  Species that can survive in the current reef matrix should be used until habitat is restored for coral-dependent species. Among those, species that rely less on having nearby mangroves and seagrass are especially important to capture and rear. | - This action is based on techniques like those of Ecocean (<http://www.ecocean.fr/en/>) - This action must be paired with other actions that would ensure good habitat to be effective for coral-dependent fish species. - Can fish be propagated in aquaria or will post-larvae have to be captured repeatedly? - To what extent is the reef ecosystem being managed for fishing versus tourism versus as a whole ecosystem? That affects how replenishment is executed, the time scale of success, and how important climate change is. - Regarding the above, which species of fish are important for rearing? How does climate change affect the viability of post-larval reef fish in aquaria? |
| 12 | Collect corals and establish aquarium-based coral nurseries  (Since this action is about the “supply side” of the coral nursery plan, this is about how the stressors affect the supply of coral for the nursery and what happens in the nursery. Planting the corals on the reef is part of a separate follow-on action, which would benefit from a resilience assessment.) | - Fewer fragments of coral colonies (especially of certain species) will be available for collection after storms due to reduced coral cover. - On the other hand, opportunities for collecting new nursery stock may increase since stock will only be collected after storms (and at construction sites). - Available coral colonies may be more resistant to higher temperatures and existing diseases (through natural selection). - Propagating coral genotypes without regard to their resilience to climate stressors will reduce action effectiveness because individuals in the nursery will have the same tolerance of climate change conditions as wild corals. | - Climate change can physically damage or destroy nurseries through more powerful storms. | - It will be 2-4 years before corals can be outplanted, so this is a medium-term action. It should probably be started immediately, to reduce long-term coral losses. On the other hand, if coastal water quality does not improve, outplanted corals may not survive. | - Preferentially grow coral genotypes that are disease-, heat-, and sediment-tolerant/resistant. In general, grow genotypes that will be able to survive projected future conditions. As conditions change further on reefs (e.g., disease outbreak, major sedimentation event), collecting new coral fragments for the nursery will introduce colonies that have survived the latest round of selective pressure. - Preferentially grow species that will restore ecosystem function by building up a protective reef structure. - Due to increased coral mortality from bleaching and disease, collect more colony fragments due to higher outplanted colony mortality. - The similarity between the water supplying the rearing aquaria and the place(s) where the corals will be outplanted also needs to be considered. Presumably, these should be similar in temperature, pH, and chemical composition. | Develop multi-species aquarium-based coral nurseries which can produce a continuous supply of coral colonies through repeated fragmentation. Species that can survive the current temperature, pH, sediment and nutrient regime should be used until water quality is restored. This may involve collecting new colony fragments that have survived widespread bleaching or disease or survived large sedimentation events.  Coral strains should be heat-tolerant (to reduce risk of bleaching) and show some resistance to the relevant coral diseases that are associated with higher temperature. They should also be effective at removing deposited sediment and maintaining growth in lower pH water. Water used in the aquaria should be from the general area where the corals will be outplanted. | - Preferentially grow corals that are disease, heat-, and sediment-tolerant/resistant. In what order should those traits be prioritized or how should they be balanced? Mote Marine is working on creating hybrids that combine these traits. - The nurseries must be large to have an ecologically meaningful amount of coral in them. How much is that? |
