# Supplementary material for: Incorporating adaptation and resilience into an integrated watershed and coral reef management plan
Source: PLoS One. 2021 Jun 24;16(6):e0253343. doi: 10.1371/journal.pone.0253343 (PMC8224911; doi:10.1371/journal.pone.0253343)
Supplement: S4 Table — (DOCX) [file pone.0253343.s005.docx]

**S4 Table. Second phase output for Adaptation Design Tool Worksheets 1A (effect of climate change on stressors) and 1B (effects of climate change on management actions and how to adapt management actions) for three management actions for the Guánica Bay watershed and associated coral reefs**

| **A1** | **A2** | **A3** | **A4** | **A5** | **A6** | **A7** |
| --- | --- | --- | --- | --- | --- | --- |
| **Action number** | **Existing Management Action** | **Target Stressor(s)** | **Climate change effects on stressors (mechanism, direction, magnitude, uncertainty)** | **Timing of climate change’s effects** | **Implications for success metrics and how to measure them** | **Notes** |
| 7 | Use water conveyance practices (e.g., water bars, culverts) to manage sediment from coffee plantation dirt roads  NOTE: Some flow diversion practices, and road stabilization measures are included with water conveyance practices because implementing them together increases their effectiveness. | Terrestrial sediment | - Storms may become more intense, leading to precipitation events that may erode dirt roads faster, particularly on steep slopes. The percent increases in erosion and runoff will likely be greater than the percent increase in precipitation (SWCS 2003; Nearing et al. 2004). High magnitude, low uncertainty. - The rainfall threshold for erosion of dirt roads in the area appears to be around 0.1 cm, with dirt released within 1-2 minutes (Ramos-Scharrón and Thomaz 2016). This threshold may be reached during a higher proportion of storms. High magnitude, low uncertainty. - Stormwater plumes may extend further into the ocean, impacting more coral reefs. High magnitude, low uncertainty. - Storms of sufficient intensity to release levels of sediment exceeding reefs’ tolerance may occur more frequently. High magnitude, medium uncertainty. - Increased resuspension of sediment in coastal waters in between storms due to stronger waves. High magnitude, medium uncertainty. - Road erosion may become more intermittent due to longer periods of drought punctuated by larger storms. More intermittent storms could themselves cause larger loads per event because drier soil will erode more easily. On the other hand, hard pan could form on roads during dry periods, causing first rain to run off without sediment. Medium magnitude, medium uncertainty. - Shifts in eroded sediment particle sizes due to larger storms, potentially including more clay particles which stay suspended in water longer and chronically expose reefs to sediment. On the other hand, larger particles (which are easier to trap) may be increased. Low magnitude, high uncertainty. | - Increasingly violent storms are already occurring. - Storm intensity will likely continue to increase over the coming decades. - Prolonged dry periods are already occurring. | **Effectiveness metrics:** Reduction by X percent of sediment loads originating from dirt roads in coffee plantations. Amount of sediment resuspension. Number of roads that need rebuilding or significant management after storms. Frequency of roads requiring regrading. Effective lifespan of conveyance structures.  **Implications for effectiveness metrics:** Loads following rain events will need to be reduced by a larger percentage to keep sediment from crossing reefs’ thresholds. Because reefs’ sediment thresholds may be crossed more often due to increased storm intensities, the impact of acute sediment loads may increase relative to the impact of chronic sediment loads. Sediment resuspension will occur more often, potentially contributing proportionately more to the sediment exposure of reefs. Road regrading will be necessary more often due to increased frequency of larger storms. Lifespan of conveyance structures will decrease from their standard duration.  **Implications for how to measure effectiveness metrics:** Water quality monitoring stations should be located at management practices and down-channel of dirt roads with and without altered water management strategies (for comparison). It will become more important to have long-term sampling that reflects extreme storms (including throughout storms). Ideally, water flow around roads would be watched during some storms to see how the management practices are working. Sampling will likely need to be able to record a broader range of sediment loads. With increasing sediment resuspension, more effort will have to be made to measure resuspension during and after storms. If road regrading is required often enough due to more frequent larger storms, it will not be an effective success metric. Perhaps the extent of road regrading needed will be more informative. | - “Expanding Conservation/Sediment Control Practices in Priority Form Areas of the Guánica Bay Watershed” (Protectores de Cuencas 2016) has extensive background on this. Says that regrading roads must be performed before other management practices are implemented. - How much are 2-, 5-, 10-, and 25-year storms expected to change by 2050? - What is the total sediment runoff reduction target for reefs? - How much of a reduction in runoff from use of road maintenance practices is necessary to reduce runoff to levels that reefs can tolerate in conjunction with other management measures? - How do road maintenance measures interact with other mountain erosion measures (e.g., shade-grown coffee) to reduce sediment? - Roads really need to be monitored during storms to detect locations and timing of most severe sediment runoff. - No management measures will protect dirt roads from large storms. There is a limit on how large a storm event dirt roads can be designed to handle. That limit may be reached more often under climate change. - Residence times of sediment particles around reefs outside Guánica Bay could determine the relative contribution to coral exposure of sediment resuspension vs. new sediment. - Puerto Rico is preparing an updated climate change effects report for Puerto Rico. - How will climate change affect the size of sediment particles mobilized? - Information on changes in runoff coefficients are necessary. - Pattern of sediment pulses on coffee plantation dirt roads under climate change is very important and needs exploration. |
| 12 | Collect corals and establish aquarium-based coral nurseries | Coral loss from:   - Warmer ocean water - Lower pH ocean water - Terrestrial sediment and nutrients - Sea level rise - Storm damage (especially for acroporids) - Anchor damage - Spills from industrial facilities - Large vessel traffic, and resulting oil spills and sediment resuspension - Stormwater from urbanized areas - Ongoing development around the Bay - Nutrients from wastewater: septic systems, and bad connections to municipal systems | - Warmer waters may increase bleaching episodes and disease outbreaks. High magnitude, low uncertainty. - Storms may become more intense with longer droughts between them, leading to precipitation events with more runoff carrying sediment and nutrients from land (SWCS 2003). The percent increases in erosion and runoff will likely be greater than the percent increase in precipitation (SWCS 2003; Nearing et al. 2004). High magnitude, low uncertainty. - Stormwater plumes may extend further into the ocean, impacting more coral reefs. High magnitude, low uncertainty. - Sediment and nutrient runoff may be exacerbated by warmer air temperatures that are expected to render soils more erosion-prone (Farrell 2014). Medium magnitude, low uncertainty. - Storms of sufficient intensity to release levels of sediment and nutrients exceeding reefs’ tolerance may occur more frequently. High magnitude, medium uncertainty. - Sea level rise may occur faster than reef accretion, leading to “sinking reefs”. Medium magnitude, low uncertainty. - Ocean acidification could decrease reproduction because colonies will need to put extra energy into skeletal growth. High magnitude, medium uncertainty. - Ocean acidification could decrease successful recruitment if corals cannot find settlement sites (e.g., due to altered chemosensory abilities) or calcify (due to acidic conditions) (Doropoulos et al. 2012- Ecology Letters, Webster et al. 2012- Global Change Biology). It may also reduce existing colonies’ growth rates. High magnitude, medium-high uncertainty. - Sediment and nutrient delivery may become more intermittent due to increased drought periods. Medium magnitude, medium uncertainty. | - Temperature effects have already occurred, with increasing magnitude through mid-century. - Increasingly violent storms are already occurring. - Storm intensity will likely continue to increase over the coming decades. - Acidification beyond coral optima may have already occurred for some taxa and is expected to worsen. | **Effectiveness metrics:** Number of colonies in nurseries. Number of climate-tolerant genotypes in nurseries, and genetic and phenotypic diversity within those genotypes. Colony survival in nurseries. Number of outplants that can be produced from nurseries. Growth rate of colonies in nurseries. Similar conditions in nurseries and outplanting destinations.  **Implications for effectiveness metrics:** Focus more on maintaining diversity of climate-tolerant genotypes, which need to be evaluated along multiple trait axes simultaneously.  **Implications for how to measure effectiveness metrics:** The preferred genotypes may need to be assessed along more trait axes simultaneously (e.g., heat, disease, acidification). Because outplanting conditions will systematically change over time (e.g., water temperature, acidity), monitoring of outplanting sites must be ongoing and systematic. | - What coral species are best to use? Taxa that can be rapidly generated through “fragging” may be preferable. - *Acropora* and *Dendrogyra* are better grown in-situ. Most other species can be grown well in aquaria. - Dave Vaughan (Mote Marine Lab) is reproducing a variety of coral species by fragmentation in lab aquaria. - There are no magic numbers for tolerance of corals to stressors. Response to any stressor affected by the co-occurring stressors (multi-stressor interactions). - Important to consider local effects on ocean, such as coastal buffering. - Some groups have been “stress-hardening” coral colonies in nurseries. - Need to track conditions at outplanting sites to make sure nursery conditions are sufficiently similar, especially for properties than can vary very locally, like acidity. Monitoring may need to be improved to do this. |
| 14 | Outplant corals on reefs around Gilligan’s Island to protect the coastline. | Coral loss from:   - Warmer ocean water - Lower pH ocean water - Terrestrial sediment and nutrients - Sea level rise - Storm damage (especially for acroporids) - Anchor damage - Spills from industrial facilities - Large vessel traffic, and resulting oil spills and sediment resuspension - Stormwater from urbanized areas - Ongoing development around the Bay - Nutrients from wastewater: septic systems, and bad connections to municipal systems | - Warmer waters may increase bleaching episodes and disease outbreaks. High magnitude, low uncertainty. - Storms may become more intense with longer droughts between them, leading to precipitation events with more runoff carrying sediment and nutrients from land (SWCS 2003). The percent increases in erosion and runoff will likely be greater than the percent increase in precipitation (SWCS 2003; Nearing et al. 2004). High magnitude, low uncertainty. - Stormwater plumes may extend further into the ocean, impacting more coral reefs. High magnitude, low uncertainty. - Sediment and nutrient runoff may be exacerbated by warmer air temperatures that are expected to render soils more erosion-prone (Farrell 2014). Medium magnitude, low uncertainty. - Storms of sufficient intensity to release levels of sediment and nutrients exceeding reefs’ tolerance may occur more frequently. High magnitude, medium uncertainty. - Sea level rise may occur faster than reef accretion, leading to “sinking reefs”. Medium magnitude, low uncertainty. - Ocean acidification could decrease reproduction because colonies will need to put extra energy into skeletal growth. High magnitude, medium uncertainty. - Ocean acidification could decrease successful recruitment if corals cannot find settlement sites (e.g., due to altered chemosensory abilities) or calcify (due to acidic conditions) (Doropoulos et al. 2012- Ecology Letters, Webster et al. 2012- Global Change Biology). It may also reduce existing colonies’ growth rates. High magnitude, medium-high uncertainty. - Sediment and nutrient delivery may become more intermittent due to increased drought periods. Medium magnitude, medium uncertainty. | - Temperature effects have already occurred, with increasing magnitude through mid-century. - Increasingly violent storms are already occurring. - Storm intensity will likely continue to increase over the coming decades. - Acidification beyond coral optima may have already occurred for some taxa and is expected to worsen. | **Effectiveness metric:** Survival and growth rates of outplanted colonies, considering maintenance of genotypic and phenotypic diversity. Use of outplanted colonies by other organisms. Sexual and asexual reproduction of colonies. Reduction in storm surge reaching coast. Reduction in coastal storm damage.  **Implications for effectiveness metric:** Survival rates over longer time periods (multiple years) may decrease due to episodic events, like storms or bleaching events. Growth rates may decrease and time until sexually reproductive may increase due to acidification. Settlement of other calcifying organisms among corals may be impacted by climate change, as well. Storm surge will become more extreme.  **Implications for how to measure effectiveness metrics:** Monitor survival for longer after outplanting because of more episodic climate change-associated events, like bleaching or storms. Monitor longer for reproduction because of delayed sexual maturity. Survival surveys should occur immediately after relevant stressor events to characterize responses of outplants, requiring rapid-response monitoring. | - Is a certain level of rugosity of outplants desirable for wave reduction? - Some micro-fragging is being used to “re-sheet” dead boulder colonies, like *Orbicella*. - Since this action is so closely related to the nursery action, it will be important to adjust nursery rearing practices in response to outplanting results. - To what extent will larger storms with longer droughts between increase the delivery of legacy PCBs to reefs? |

| **B1** | **B2** | **B3** | **B4** | **B5** | **B6** | **B7** | **B8** |
| --- | --- | --- | --- | --- | --- | --- | --- |
| **Action number** | **Existing management action** | **Changes in effectiveness of management action due to: climate impacts on target stressor** | **Changes in effectiveness of management action due to: climate impacts on management action** | **Time frame or constraint for using the action and implementation (e.g., urgency, longer or shorter term)** | **What changes are needed to adapt the action (place, time, and engineering design)** | **Climate-Smart Management Action** | **Notes** |
| 7 | Use water conveyance practices (e.g., water bars, culverts) to manage sediment from coffee plantation dirt roads  NOTE: Some flow diversion practices and road stabilization measures are included with water conveyance practices because implementing them together increases their effectiveness. | - Water diversion structures may not be able to divert all water off or under roads, producing downhill road erosion. - Interstices between stones in check dams or swales may become clogged with sediment more quickly from larger or more frequent storms. This will reduce the cross-sectional area available for water to flow through and the area available for conveying water. - Flow and erosion reduction practices may not be able to slow down water sufficiently to prevent further erosion and trap sediment. - Vertical dirt walls along roads may be more likely to collapse under increased precipitation. Sediment trapping structures may not be able to handle the resulting increased loads. | - Larger storms may wash out existing water conveyance structures (e.g., water bars, the stones comprising check dams, or culverts). - Larger storms may wash out existing flow and erosion reduction practices (e.g., vetiver and rock check dams). - Larger storms may overflow culverts or wash them out entirely, leading to stream crossings being washed out. - Road regrading may be complicated by increased risk of raised road banks slipping onto road during or after regrading. Slippage is already happening but could happen more under some precipitation scenarios. - Droughts may compromise the effectiveness of vegetative solutions. Vetiver probably does fine in droughts; native plants (investigated by the Fish and Wildlife Service) may not do as well. - Work completed less than one week to one month before a large storm may be undone, i.e. completed project can take a month to establish. Depending on the pattern of large storms, this could increase, decrease, or simply shift the window for completing projects. | - This suite of actions can be implemented immediately. They have the potential to quickly affect sediment loads. | - Compact dirt roads that have been topped with aggregated crushed stone. - Make road crown higher to consistently drain water to sides of road, if not using insloped or outsloped roads. - Implement stronger and more frequent debris barriers upstream of culverts to prevent culvert clogging. - Increase the size of culverts to some standard minimum size that will handle storms of a specified size (e.g., the predicted 5-year storm). - Use larger rip-rap stones or more extensive vetiver patches on downstream sides of culverts to diffuse faster and larger flows. - Increase frequency of rolling dips or water bars on steeper-sloped roads or stretches likely to experience heavy erosion. - Clean accumulated sediment out of interstices between sediment trap rocks more frequently. - Roads on particularly steep slopes may need to be paved (with asphalt or concrete) if other management actions are not working. - Build maintenance into construction of the projects. Maintenance will have to be more frequent. - Use vegetation that can withstand both dry periods and stronger flows. | Minimize sediment from existing dirt mountain roads by building water diversions more frequently along roads, sloping roads more heavily to promote faster drainage, and augmenting barriers on upstream sides of culverts and flow diffusers on downstream sides of culverts. Culvert size should be increased to a standard minimum size in preparation for consistently larger flows. Locations requiring flow control may change due to altered precipitation patterns. Check integrity and repair diversion structures after larger storms; remove sediment from sediment traps after large storms. Compact roads with surfaces made of small rocks and granular material to stabilize road surfaces. Pave roads that have already repeatedly washed out if other mitigation techniques are not possible. | - These structures and practices are considered together (as a suite of actions) because they must be implemented in combination in order to be effective. - To what extent can dirt roads traversing hills and mountains be retrofitted? - Which roads is it most important to work on? - This is a feedback loop: the worse the road water management actions perform, the more erosion there will be, and the worse they will perform. - When is paving dirt roads an option? Are they generally too temporary to justify paving? - Dirt roads can be stable for a long time if managed properly. However, once they begin to unravel, they can fall apart very quickly. They have a failure threshold and maintenance keeps them from reaching that threshold. They have passed that threshold by the time they are impassible. - Road maintenance is not generally funded in grants, yet it is essential for long-term performance of structures. Thus, maintenance falls to the property owners (farmers). Pretty much all maintenance will be affected by climate change. - No-cost extensions would increase flexibility in completing work under greater weather uncertainty. - Climate change may reduce the lifespans of projects. Standard lifespan now is 25 years. - Project locations are based on which farmers are willing to collaborate, as well as slope, potential of land to erode, connectivity to water bodies, and traffic load. To what extent will climate change affect these? - Supply farmers with culverts of the right size so they are not just using whatever they have handy. |
| 12 | Collect corals and establish aquarium-based coral nurseries  (Since this action is about the “supply side” of the coral nursery plan, this is about how the stressors affect the supply of coral for the nursery and what happens in the nursery. Planting the corals on the reef is part of a separate follow-on action.) | - Fewer fragments of coral colonies (especially of certain species) will be available for collection after storms due to reduced coral cover. - On the other hand, opportunities for collecting new nursery stock may increase since stock will only be collected after storms (and at construction sites). - Available coral colonies may be more resistant to higher temperatures and existing diseases (through natural selection). - Propagating coral genotypes without regard to their resilience to climate stressors will reduce action effectiveness because individuals in the nursery will have the same tolerance of climate change conditions as wild corals. - Conditions at outplanting sites will have directional trends over time (acidity, temperature, sediment and nutrient delivery). If field conditions are not monitored, there may eventually be a mismatch between nursery and outplant site conditions, which will incompletely prepare colonies for outplanting. | - Risk of disruption to nursery operations, such as power failures, may increase due to larger storms. - Nurseries must be prepared for power failures, loss of clean water, and other emergencies. | - Some coral species can be raised to outplanting within one year. Nurseries should probably be started immediately, to counter long-term coral losses. On the other hand, if coastal water quality does not improve, outplanted corals may not survive. | - Preferentially grow coral genotypes that are disease-, heat-, low pH, and sediment-tolerant/resistant, while maintaining phenotypic diversity of colonies. In general, grow genotypes that will be able to survive projected future conditions. As conditions change further on reefs (e.g., disease outbreak, major sedimentation event), collecting new coral fragments for the nursery will introduce colonies that have survived the latest round of selective pressure. - Preferentially grow species that will restore ecosystem function. - Due to increased coral mortality from bleaching and disease, collect more colony fragments due to higher mortality of outplanted colonies. - The similarity between the water supplying the rearing aquaria and the place(s) where the corals will be outplanted also needs to be considered. These should be similar in temperature, pH, light, nutrient/sediment levels, and chemical composition. Site conditions will need to be monitored throughout time to achieve this, since there will be chronic trends on top of natural variability. - Install a backup generator at nurseries to provide power during outages caused by larger storms. Have a plan ready to maintain or relocate organisms due to natural disasters. | Develop multi-species aquarium-based coral nurseries which can produce a continuous supply of coral colonies through repeated fragmentation. Coral strains should be heat-tolerant (to reduce risk of bleaching) and show some resistance to the relevant coral diseases that are associated with higher temperature, while maintaining phenotypic diversity. They should also be effective at removing deposited sediment and maintaining growth in lower pH water.  This may involve collecting new colony fragments that have survived widespread bleaching or disease or survived large sedimentation events. It may be necessary to collect more colony fragments for a successful program.  Water properties used in the aquaria should be similar to that from where the corals will be outplanted in terms of temperature, pH, light, nutrient/sediment levels, and chemical composition. Water at the outplanting sites will need to be monitored through time to ensure that nursery conditions are kept as similar as possible. The nurseries should have disaster plans and backup generators installed to mitigate the increased risk of power outages. | - In what order should heat, disease, and sediment tolerance be prioritized or how should they be balanced? Many organizations are working on creating hybrids that combine these traits. - The nurseries must be large to have an ecologically meaningful amount of coral in them. How much is that? |
| 14 | Outplant corals on reefs around Gilligan’s Island to protect the coastline. | - Outplanting sites selected because they are currently in deep water may be in too deep water in the future due to sea level rise. - Turbidity or coastal erosion plumes/hotspots may change in such a way that outplanting locations selected to avoid hotspots or plumes may no longer do so. - Outplanting locations not currently exposed to legacy sediment contaminants could become exposed from increased resuspension or runoff. - Decreased growth and reproduction rates of outplants from ocean acidification. - Above stressor effects may reduce the genetic diversity of outplanted colonies below the intended level. | - Increased physical destruction of outplanted colonies by storms, especially for more delicate species. - Increased death of outplanted colonies from bleaching and diseases. | - The sooner this is done, the better. - Outplanting will have to be a sustained, long-term effort. | - Focus on outplanting coral strains with a variety of types of tolerance to climate-change effects, including being bred to tolerate multiple stressors. - May need to focus on outplanting at shallower sites due to sea level rise, although that must be balanced against the impacts of larger storm and terrestrial runoff at shallower depths (in general). - If outplanting in deep water, focus on using species with a broad depth range to account for SLR. - Outplant some aquarium-bred colonies with the hope that they will be naturally fragmented and propagate themselves, and outplant others to build up reef where they are. - Cement may need to be used more often to attach colonies to substrate due to increased risk from storms. - Time outplanting to avoid periods with more runoff and land-based pollution. - Factor changes in sediment plume location and direction into outplanting site selection. - Monitoring may need to be extended longer after outplanting to include observation of how outplants handle extreme events and to what extent they reproduce. | Outplant colonies that are heat-, disease-, sediment-, and low pH-tolerant. Species mix should be optimized for robust coastal defense. Outplanting during high sediment or precipitation periods should be avoided to reduce initial stressor exposure to outplants.  Balance outplanting locations between deeper and shallower sites, accounting for how sea level rise may make deeper sites inhospitable in the future and how shallower sites may be more heavily exposed to land-based pollution. Colonies in shallower sites may need to be affixed to reefs using cement more frequently due to larger storms. Colonies in deeper sites should have a broad range of depth tolerances. In either case, it may be desirable to locate branching colonies where they will be naturally fragmented. Site selection may also be affected by shifting plumes of land-based pollutants from the bay.  Because survival through extreme events is an important part of the nursery program, colonies should be monitored through extreme events such as high temperatures and large storms. Monitoring periods for outplants may need to be extended to do this. | - Can bathymetry be used to predict where storms will be most or least damaging? - Reefs to the west of Guánica Bay receive more sedimentation than those to the east. Reef resilience assessment shows some potentially suitable sites to the east of the bay. - Most people are attaching colonies individually at this point. What can be done to usefully attach multiple colonies simultaneously? - Need to focus on other activities that are important for improving the outplanting environment. |
