# Supplementary material for: Incorporating adaptation and resilience into an integrated watershed and coral reef management plan
Source: PLoS One. 2021 Jun 24;16(6):e0253343. doi: 10.1371/journal.pone.0253343 (PMC8224911; doi:10.1371/journal.pone.0253343)
Supplement: S5 Table — (DOCX) [file pone.0253343.s006.docx]

**S5 Table. Five-column summaries of Adaptation Design Tool Worksheets 1A and 1B (effect of climate change on stressors and effects of climate change on management actions and how to adapt management actions, respectively) for 12 management actions for the Guánica Bay watershed and associated coral reefs**

| **Existing management action, and target stressor(s)** | **Climate change effects on stressor(s)** | **Impacts of climate change on existing management action (direct & indirect)** | **Climate-smart management action** | **Refs.** |
| --- | --- | --- | --- | --- |
| 1. Plant cover crops in Guánica Valley farms to manage terrestrial sediment and nutrients | - More intense storms may produce runoff carrying more sediment and nutrients from land. Percent increases in erosion and runoff likely greater than percent increase in precipitation. High magnitude, high certainty. - Stormwater plumes may extend further into the ocean, impacting more coral reefs. High magnitude, high certainty. - Sediment and nutrient runoff may be exacerbated by more erosion-prone soils. Medium magnitude, high certainty. - Storms of sufficient intensity to release levels of sediment and nutrients exceeding reefs’ tolerance may occur more frequently. High magnitude, medium certainty. - More intermittent sediment and nutrient delivery may occur. Medium magnitude, medium certainty. | - Cover crops may become less effective at retaining sediment and nutrients due to more intense precipitation events and more rain. - Soil may become more erosion-prone if it becomes drier and more compacted. - Cover crops may be uprooted by more powerful surface flows from stronger storms, especially in steeper sloped areas (valley sides). - Cover crops may not be sufficiently drought- and heat-resistant to survive and retain soils made drier by warmer air and more intermittent precipitation. - Timing of cover crop planting may need to be altered so that cover crops provide maximal soil coverage during the strongest storms. - Large storms between cash crop harvesting and cover crop planting could cause ponding in poor drainage areas, making it harder to plant cover crops. - Timing of cover crop termination may need to be shifted due to altered soil moisture. | Annually plant a mixture of cover crops that are drought- and flood-resistant, timing it such that the cover crops will protect soil from rain during the strongest storms. Preferentially plant cover crops near irrigation channels and on more erosion-prone soils. Monitor and be prepared to replant cover crops in case of damage from extreme events. | ^[[1]](#footnote-2)^, ^[[2]](#footnote-3)^, ^[[3]](#footnote-4)^, ^[[4]](#footnote-5)^, ^[[5]](#footnote-6)^ |
| 2. Plant riparian buffers along the Rio Loco where it passes through farms to manage terrestrial sediment and nutrients | - More intense storms may produce runoff carrying more sediment and nutrients from land, especially on high slope farms along the Rio Loco. Percent increases in erosion and runoff likely greater than percent increase in precipitation. High magnitude, high certainty. - Stormwater plumes may extend further into the ocean, impacting more coral reefs. High magnitude, high certainty. - Sediment and nutrient runoff may be exacerbated by more erosion-prone soils. Medium magnitude, high certainty. - Storms of sufficient intensity to release levels of sediment and nutrients exceeding reefs’ tolerance may occur more frequently. High magnitude, medium certainty. - More intermittent sediment and nutrient delivery may occur. Medium magnitude, medium certainty. | - Riparian buffers may not be wide enough to slow surface runoff from stronger storms sufficiently to let sediment settle out and prevent formation of channels through the buffers. - Buffers may not be wide enough to allow adequate infiltration for roots to absorb dissolved nutrients. - Runoff balance could shift between subsurface flow and surface runoff, allowing more runoff to bypass roots and reach waterbodies. - Soil may become more erosion-prone (if it becomes drier and more compacted), producing more sediment and surface runoff. - Buffers could be eroded more easily by more powerful surface flows in stronger storms. - Buffers nearest the streams/Rio Loco could be eroded more easily by larger in-stream flows. - Buffer vegetation may need to be more drought- and heat-resistant to survive and retain soils made drier by warmer air and more intermittent precipitation. - Wherever the Rio Loco or its tributaries are very deeply incised, the roots of buffer vegetation may not extend deep enough to reach streamflow or groundwater, and therefore may not hold banks together, may not intercept subsurface flow, and may die. - Grass buffer strips may need to be maintained (cleaned out) more often because of faster sediment accumulation from larger storms. | Plant riparian buffers that will be an adequate width to withstand and intercept potentially larger surface and subsurface flows across farms. The buffers should be planted in locations that will intercept the maximum runoff possible (probably determined by hydrological models). Buffer plants should be able to withstand larger in-stream flows and across-farm flows, hold together drier soils, and withstand higher air temperatures with potentially longer dry periods. Buffers may need to be checked and replanted more frequently because of increased storm intensity. | ^[[6]](#footnote-7)^, ^[[7]](#footnote-8)^, ^[[8]](#footnote-9)^, ^[[9]](#footnote-10)^, ^[[10]](#footnote-11)^, ^[[11]](#footnote-12)^, ^[[12]](#footnote-13)^ |
| 3. Replace sun-grown coffee with shade-grown coffee to manage terrestrial sediment and nutrients | - More intense storms may produce runoff carrying more sediment and nutrients from land, especially on high slope coffee farms. Percent increases in erosion and runoff likely greater than percent increase in precipitation. High magnitude, high certainty. - Stormwater plumes may extend further into the ocean, impacting more coral reefs. High magnitude, high certainty. - Sediment and nutrient runoff may be exacerbated by more erosion-prone soils. Medium magnitude, high certainty. - Storms of sufficient intensity to release levels of sediment and nutrients exceeding reefs’ tolerance may occur more frequently. High magnitude, medium certainty. - More intermittent sediment and nutrient delivery may occur. Medium magnitude, medium certainty. | - Erosion may increase if stronger storms mean that more rain penetrates the shade canopy and reaches the soil. - Increased peak intensity of precipitation may increase erosion. - Higher temperatures may be less suitable for certain shade trees, potentially decreasing their effectiveness. - In warmer air, the decomposition rate of shade tree litter may increase. This could affect nutrient cycling in unknown ways. - Warmer temperatures may shift the balance between coffee plants/shade trees and their herbivores and parasites. How those community interactions will be altered are very uncertain, but negative outcomes could include increased herbivory/parasitism of coffee plants and their shade plants. | Plant shade-tolerant coffee with heat-tolerant shade plants, with priority to more erosion-prone slopes. Plant understory/secondary canopy plants that will effectively use or provide nutrients (e.g., n-fixation if nitrogen is likely to become more limiting) and stabilize soil during more intense storms. Prune shade plants as necessary to allow adequate light and rain to reach coffee plants. | ^[[13]](#footnote-14)^, ^[[14]](#footnote-15)^, ^[[15]](#footnote-16)^, ^[[16]](#footnote-17)^, ^[[17]](#footnote-18)^, ^[[18]](#footnote-19)^, ^[[19]](#footnote-20)^ |
| 4. Hydroseed bare soils associated with roads and homes to manage terrestrial sediment and nutrients | - More intense storms may produce runoff carrying more sediment and nutrients from land. Percent increases in erosion and runoff likely greater than percent increase in precipitation. High magnitude, high certainty. - Stormwater plumes may extend further into the ocean, impacting more coral reefs. High magnitude, high certainty. - Sediment and nutrient runoff may be exacerbated by more erosion-prone soils. Medium magnitude, high certainty. - Storms of sufficient intensity to release levels of sediment and nutrients exceeding reefs’ tolerance may occur more frequently. High magnitude, medium certainty. - More intermittent sediment and nutrient delivery may occur. Medium magnitude, medium certainty. | - Hydroseeded areas may become less effective at retaining soil due to more intense precipitation events and more rain overall. - Soil may become more erosion-prone if it becomes drier and more compacted. - Altered precipitation regime and soil properties may change the kind of erosion (e.g., gulley vs. mass wasting) along road slopes, which may affect the appropriate species for used in hydroseeding. - Deployed hydroseeding mixture or germinated plants could be uprooted more easily by more powerful surface flows in stronger storms. - Hydroseeded locations may have longer dry periods, increasing the risk of seed or grass mortality which could expose more soil surface and increase the potential for erosion and gully formation. - Hydroseeded grass may grow faster due to increased CO_2_ availability, shortening the time until it becomes effective. | Hydroseed bare soils that are especially erosion-prone. Use a hydroseeding mixture that will be able to effectively bind the soil during stronger storms until and after the plants germinate. The plants should be selected to protect soil during longer dry periods. Monitor slopes following large storms and be prepared to reseed more frequently. | ^[[20]](#footnote-21)^, ^[[21]](#footnote-22)^, ^[[22]](#footnote-23)^ |
| 5. Construct or retrofit swales to manage sediment, nutrients, and chemicals in urban stormwater | - More intense storms may produce runoff carrying more sediment, nutrients, and chemicals from land. Percent increases in erosion and runoff likely greater than percent increase in precipitation. High magnitude, high certainty. - Sediment and nutrient runoff may be exacerbated by more erosion-prone soils. Medium magnitude, high certainty. - Storms of sufficient intensity to release levels of sediment and nutrients exceeding reefs’ tolerance may occur more frequently. High magnitude, medium certainty. - More intense storms may erode soils with higher concentrations of legacy contaminants. Medium magnitude, low certainty. | - Swales may be flooded by more intense storms producing larger runoff volumes, thereby allowing stormwater to avoid infiltration. - Water may stay in swales longer if infiltration rates are slower, keeping swale water levels higher. - Swales may fill more rapidly with sediment and debris (trash, plants, etc.) that are carried by stormwater. - Salty Bay water may flood swales closest to the coast during storms, killing freshwater plants and reducing volume available for urban stormwater. - Larger storms may cause swales to erode, releasing more sediment. - Longer dry periods may hurt or kill plants in swales. | Construct swales in areas that will not likely be flooded by storm surges or use saltwater-tolerant plants in swales if they are likely to be repeatedly inundated by storm surges. Size new ones or resize existing ones according to projected future storm intensities, with side slopes that are less erodible. Size them to retain greater volumes of water, for longer after storms, and handle more debris and sediment. | ^[[23]](#footnote-24)^, ^[[24]](#footnote-25)^ |
| 6. Restore Guánica Lagoon to manage terrestrial sediment and nutrients | - More intense storms may produce runoff carrying more sediment and nutrients from land. Percent increases in erosion and runoff likely greater than percent increase in precipitation. High magnitude, high certainty. - Stormwater plumes may extend further into the ocean, impacting more coral reefs. High magnitude, high certainty. - Sediment and nutrient runoff may be exacerbated by more erosion-prone soils. Medium magnitude, high certainty. - Storms of sufficient intensity to release levels of sediment and nutrients exceeding reefs’ tolerance may occur more frequently. High magnitude, medium certainty. - More intermittent sediment and nutrient delivery may occur. Medium magnitude, medium certainty. | - Lagoon restoration may not be sufficient to manage the greater amounts of sediment, nutrients, and debris due to stronger storms that will cause more Lajas Valley irrigation ditch erosion (more precipitation, larger peak flows). - Drier, more compact soil could be eroded more easily, exacerbating over-loading of lagoon’s retention capacity. - Lagoon may become more effective at nutrient removal; some evidence shows that warmer water leads to faster nutrient uptake. - Maintaining preferred water depth in the lagoon could become more difficult during more intense storms. Also, longer dry periods and higher temperatures could make water levels too low. - Warmer temperatures could harm the lagoon’s flora and fauna, reducing nutrient processing efficiency. - More rapid sediment accumulation could alter flow through the lagoon or fill it up. - Storm surge reaching further inland from larger storms on top of sea level rise could introduce salty Bay water to the lagoon and kill flora. | Restore Guánica Lagoon to handle flows and associated sediment loads projected by climate models. Consider extra sediment settling or diversion mechanisms to keep the lagoon from filling up with larger sediment loads during storms, and additional sediment removal following storms. The lagoon should also be designed to handle potentially longer dry periods, during which water levels will not be replenished. The lagoon should be physically protected from storm surges by berms of adequate height. | ^[[25]](#footnote-26)^, ^[[26]](#footnote-27)^, ^[[27]](#footnote-28)^, ^[[28]](#footnote-29)^ |
| 7. Use water diversion structures and flow reduction practices (e.g., water bars, vetiver and rock check dams, culverts) to manage sediment from coffee plantation dirt roads | - More intense storms may erode dirt roads faster. Percent increases in erosion and runoff likely greater than percent increase in precipitation. High magnitude, high certainty. - The rainfall threshold for erosion of dirt roads appears to be around 0.1 cm, with dirt released within 1-2 minutes. This threshold may be reached during a higher proportion of storms. High magnitude, low uncertainty. - Stormwater plumes may extend further into the ocean, impacting more coral reefs. High magnitude, high certainty. - Storms of sufficient intensity to release levels of sediment and nutrients exceeding reefs’ tolerance may occur more frequently. High magnitude, medium certainty. - More intermittent road erosion due to changing precipitation patterns may occur. Medium magnitude, medium certainty. | - Water diversion structures may not be able to divert all water off roads, producing downhill road erosion. - Flow and erosion reduction practices may not be able to slow down water sufficiently to prevent further erosion and trap sediment. - Vertical dirt walls along roads may be more likely to collapse under increased precipitation. - Interstices between stones in check dams or stone swales may become clogged with sediment more quickly from larger or more frequent storms. - Larger storms may wash out existing water diversion structures (e.g., water bars). - Larger storms may wash out existing flow and erosion reduction practices (e.g., vetiver and rock check dams). - Larger storms may overflow culverts or wash them out entirely. - Road regrading may be complicated by increased risk of raised road banks slipping onto road during or after regrading. Slippage is already happening but could happen more under some precipitation scenarios. | Minimize sediment from existing dirt mountain roads by building water diversions fore frequently along roads, sloping roads more heavily to promote faster drainage, and augmenting barriers on upstream sides of culverts and flow diffusers on downstream sides of culverts. Locations requiring flow control may change due to altered precipitation patterns. Check integrity and repair diversion structures after larger storms; remove sediment from sediment traps after large storms. Compact roads with surfaces made of small rocks and granular material to stabilize road surfaces. Pave roads that have already repeatedly washed out if other mitigation techniques are not possible. | ^[[29]](#footnote-30)^, ^[[30]](#footnote-31)^, ^^[[31]](#footnote-32)^^, ^[[32]](#footnote-33)^ |
| 8. Construct wetlands for tertiary treatment at the Guánica WWTP to manage nutrients released from the WWTP | Climate change is not likely to affect nutrient input to the wetland because:   - What and how much effluent is released from secondary treatment and enters the wetlands is human-controlled, and; - The catchment for the constructed wetlands is small and runoff to the wetlands is minimal. | - Wetlands may become more effective at nutrient removal because warmer water can lead to faster nutrient uptake. - Maintaining optimal water depth (1-2 ft.) for effective treatment beds could become more difficult during more intense storms. Also, longer dry periods and higher temperatures could lower water levels below that of a functional wetland. - Warmer water in wetlands may be injurious or lethal to the current plant community (whether it is natural or human assembled). - With sea levels expected to rise between 0.5 and 2 m by 2100, saltwater intrusion could occur, or storm surges could overtop the protective berms (2.2 m above MSL), exposing freshwater wetland plants to lethal salinities. | Construct tertiary treatment wetlands at the GB WWTP using grass species that are tolerant of warmer water. Use linings to protect from saltwater intrusion, or else prepare for a shift to salt tolerant plants in the future. The protective berms should be tall enough to withstand storm surge from higher sea levels and potentially more powerful storms. Water level control in the cells will need to consider greater evaporation rates and larger storms. | ^[[33]](#footnote-34)^, ^[[34]](#footnote-35)^,  Pers. comm from Evelyn Huertas and Paul Sturm |
| 9. Protect seagrass meadows to manage terrestrial sediment and nutrient runoff; to manage coastal and near-shore erosion and sediment release (seagrass protects shoreline and binds sediment); and, to improve inter-habitat connectivity (seagrass provides nursery habitat for young fish stock and hunting grounds for adults) | - More intense storms may produce runoff carrying more sediment and nutrients from land. Percent increases in erosion and runoff likely greater than percent increase in precipitation. High magnitude, high certainty. - Stormwater plumes may extend further into the ocean, impacting more coral reefs. High magnitude, high certainty. - Coastal erosion and near-shore sediment resuspension may increase with stronger storms. Medium magnitude, high certainty. - Olfactory abilities of fish may be altered due to ocean acidification, which could reduce inter-habitat connectivity by reducing larvae’s ability to find seagrass nurseries, juvenile fishes’ ability to find the coral reef once they’re ready to move out of the seagrass, and adult fishes’ ability to nocturnally forage in seagrass. Unknown magnitude, medium certainty. | - Seagrass acreage may be inadequate to filter or trap enough sediment/nutrients in runoff produced by larger storms. - Seagrass acreage and density may be inadequate to sufficiently mitigate resuspension of sediments during larger storms. - Protected seagrass meadows may not be optimally located to maintain nursery and predation ground connectivity with reefs. - Seagrass acreage may be inadequate for fish nurseries. - Warming water may kill seagrasses that are near the upper limits of their thermal tolerance. - Rising sea levels will reduce light reaching seagrasses at marginal depths and may slow their growth. - Epiphytic or benthic algae could have a competitive advantage over seagrasses due to slower seagrass growth rates. - Stronger and/or more frequent storms will increase the disturbance regime, which could kill more seagrasses. - Increased CO_2_ in the water could alter competition between species by differentially affecting growth rates of species. The resulting shift in seagrass community composition could affect terrestrial sediment and nutrient baffling and connectivity to reefs. | Preferentially protect seagrass meadows between large freshwater inputs and priority reef tracts, especially in shallower locations.  Protect seagrass meadows that will maintain their function as nurseries and feeding grounds given potentially altered abilities of fish to complete habitat shifts (meadows nearer reefs). | ^[[35]](#footnote-36)^, ^[[36]](#footnote-37)^, ^[[37]](#footnote-38)^, ^[[38]](#footnote-39)^, ^[[39]](#footnote-40)^, ^[[40]](#footnote-41)^, ^[[41]](#footnote-42)^ |
| 10. Protect mangrove forests to manage terrestrial sediment and nutrient runoff; to manage coastal erosion (seagrass protects shoreline and binds sediment); and, to improve inter-habitat connectivity (mangroves provide nursery habitat for young fish) | - More intense storms may produce runoff carrying more sediment and nutrients from land. Percent increases in erosion and runoff likely greater than percent increase in precipitation. High magnitude, high certainty. - Stormwater plumes may extend further into the ocean, impacting more coral reefs. High magnitude, high certainty. - Coastal erosion and near-shore sediment resuspension may increase with stronger storms. Medium magnitude, high certainty. - Olfactory abilities of fish may be altered due to ocean acidification, which could reduce inter-habitat connectivity by reducing either larvae’s ability to find mangrove nurseries or juvenile fishes’ ability to find the coral reef once they’re ready to move out of the mangroves. Unknown magnitude, medium certainty. | - Extent of mangroves may be inadequate to filter or trap enough terrestrial runoff produced by larger storms. - Extent of mangroves may be inadequate to prevent excessive coastal erosion. - Protected mangroves may not be optimally located to maintain nursery connectivity with reefs. - Extent of mangroves may be inadequate for fish nurseries. - Stronger and/or more frequent storms will increase the likelihood of mangrove destruction and severe coastal erosion. - If mangrove sediment accretion rates are lower than sea level rise rates, mangrove forests retreat landward. If there is limited space for landward migration, the mangrove forest will become narrower. - More intense storms interspersed with longer dry periods will cause salinity in mangrove forests to fluctuate more, which will decrease productivity. - Higher temperatures and higher CO_2_ levels could increase productivity of mangroves, though this depends on species, salinity, etc. | Protect mangroves that will be positioned to intercept as much runoff as possible from climate-altered hydrology. Also, protect mangroves that serve as climate refugia for reef organisms and that are likely to continue to be nurseries, taking into account potentially altered reef-mangrove connectivity.  Where possible, preserve landward space for mangroves to retreat into if necessary. | ^[[42]](#footnote-43)^, ^[[43]](#footnote-44)^, ^[[44]](#footnote-45)^, ^[[45]](#footnote-46)^, ^[[46]](#footnote-47)^, ^[[47]](#footnote-48)^, ^[[48]](#footnote-49)^ |
| 11. Capture larval fish of target species and establish reef fish aquarium-based nurseries, for later use in replenishing reef fish populations depleted by fishing pressure and reduced fish recruitment  (Since this action is about the “supply side” of the fish replenishment plan, this is about how climate change affects the supply of larvae for the management action and what happens in the nursery. Releasing the fish is part of a separate follow-on action, which would benefit from a resilience assessment.) | - Water temperature and chemistry changes could alter fish recruitment to reefs due to range shifts or increased mortality. High magnitude, low certainty. - Fishing pressure around Guánica may increase as land-based livelihoods (e.g., farming) become more challenging. Medium magnitude, low certainty. - Outbreaks of fish diseases may increase with warmer water. Low magnitude, medium certainty. - The number of fishing days may be reduced due to more intense storms, concentrating fishing into a smaller number of days per year, which may intersect differently with fish spawning and recruitment periods or locations. Medium magnitude, low certainty. - Fishing infrastructure may be impaired due to more intense storms, reducing fishing pressure. Low magnitude, low certainty. | - Supply of post-larvae for the rearing program may be affected by changing water chemistry and temperature. These could alter spawning timing, location, and amount of reproductive fish, the effects from which may be perpetuated from pelagic development to post-larval return and capture. | Establish reef fish aquarium-based nurseries that use water similar to where the fish will be released. Capture and rear more fish to release since mortality may be higher from multiple causes during rearing and after release. Post-larvae may be found in different places due to altered water chemistry and circulation.  Species that can survive in the current reef matrix should be used until habitat is restored for coral-dependent species. Among those, species that rely less on having nearby mangroves and seagrass are especially important to capture and rear. | ^[[49]](#footnote-50)^, ^[[50]](#footnote-51)^, ^[[51]](#footnote-52)^, ^[[52]](#footnote-53)^, ^[[53]](#footnote-54)^, ^[[54]](#footnote-55)^, ^[[55]](#footnote-56)^, ^[[56]](#footnote-57)^ |
| 12. Collect corals and establish aquarium-based coral nurseries, to counteract coral loss from warmer ocean water, lower pH water, terrestrial sediment and nutrients, and sea level rise  (Since this action is about the “supply side” of the coral nursery plan, this is about how the stressors affect the supply of coral for the nursery and what happens in the nursery. Planting the corals on the reef is part of a separate follow-on action, which would benefit from a resilience assessment.) | - Warmer waters may increase bleaching episodes and disease outbreaks. High magnitude, high certainty. - More intense storms may produce runoff carrying more sediment and nutrients from land. Percent increases in erosion and runoff likely greater than percent increase in precipitation. High magnitude, high certainty. - Stormwater plumes may extend further into the ocean, impacting more coral reefs. High magnitude, high certainty. - Sediment and nutrient runoff may be exacerbated by more erosion-prone soils. Medium magnitude, high certainty. - Storms of sufficient intensity to release levels of sediment and nutrients exceeding reefs’ tolerance may occur more frequently. High magnitude, medium certainty. - Sea level rise may occur faster than reef accretion, leading to “sinking reefs”. Medium magnitude, high certainty. - Ocean acidification could decrease successful recruitment if corals cannot find settlement sites (e.g., due to altered chemosensory abilities) or calcify (due to acidic conditions). It may also reduce existing colonies’ growth rates while increasing dissolution rates. High magnitude, medium-low certainty. - More intermittent sediment and nutrient delivery may occur. Medium magnitude, medium certainty. | - Fewer fragments of coral colonies (especially of certain species) will be available for collection after storms due to reduced coral cover. - On the other hand, opportunities for collecting new nursery stock may increase since stock will only be collected after storms (and at construction sites). - Available coral colonies may be more resistant to higher temperatures and existing diseases (through natural selection). - Propagating coral genotypes without regard to their resilience to climate stressors will reduce action effectiveness because individuals in the nursery will have the same tolerance of climate change conditions as wild corals. | Develop multi-species aquarium-based coral nurseries which can produce a continuous supply of coral colonies through repeated fragmentation. Species that can survive the current temperature, pH, sediment and nutrient regime should be used until water quality is restored. This may involve collecting new colony fragments that have survived widespread bleaching or disease or survived large sedimentation events.  Coral strains should be heat-tolerant (to reduce risk of bleaching) and show some resistance to the relevant coral diseases that are associated with higher temperature. They should also be effective at removing deposited sediment and maintaining growth in lower pH water. Water used in the aquaria should be from the general area where the corals will be outplanted. | ^[[57]](#footnote-58)^, ^[[58]](#footnote-59)^, ^[[59]](#footnote-60)^, ^[[60]](#footnote-61)^, ^[[61]](#footnote-62)^ |

1. Puerto Rico Climate Change Council (PRCCC). 2013. “Puerto Rico’s State of the Climate 2010-2013: Assessing Puerto Rico’s Social-Ecological Vulnerabilities in a Changing Climate.” Puerto Rico Coastal Zone Management Program, Department of Natural and Environmental Resources, NOAA Office of Ocean and Coastal Resource Management. San Juan, PR. [↑](#footnote-ref-2)
2. Farrell P. 2014. The impact of climate change on soil erosion. *REACCH Annual Report*, Year 4. [↑](#footnote-ref-3)
3. Nearing, M, Pruski, F, and O’Neal, M. 2004. Expected climate change impacts on soil erosion rates: a review. *Journal of Soil and Water Conservation* **59(1)**: 43-50. [↑](#footnote-ref-4)
4. Soil and Water Conservation Society. 2003. *Conservation implications of climate change: Soil erosion and runoff from cropland*. [↑](#footnote-ref-5)
5. USDA Natural Resources Conservation Service. No date. http://www.nrcs.usda.gov/wps/portal/nrcs/detail/pr/soils/health/?cid=stelprdb1252178. Accessed October 2016. [↑](#footnote-ref-6)
6. Farrell P. 2014. The impact of climate change on soil erosion. *REACCH Annual Report*, Year 4. [↑](#footnote-ref-7)
7. Nearing, M, Pruski, F, and O’Neal, M. 2004. Expected climate change impacts on soil erosion rates: a review. *Journal of Soil and Water Conservation* **59(1)**: 43-50. [↑](#footnote-ref-8)
8. Soil and Water Conservation Society. 2003. *Conservation implications of climate change: Soil erosion and runoff from cropland*. [↑](#footnote-ref-9)
9. USDA Natural Resources Conservation Service. No date. http://www.nrcs.usda.gov/wps/portal/nrcs/detail/pr/newsroom/stories/?cid=nrcseprd888883. Accessed October 2016. [↑](#footnote-ref-10)
10. Scatena F. 1990. Selection of riparian buffer zones in humid tropical steeplands. *Proceedings of the Fiji Symposium* **192:** 328-337. [↑](#footnote-ref-11)
11. Department of Natural and Environmental Resources. 2011. “Puerto Rico Forest Action Plan.” [↑](#footnote-ref-12)
12. North Caroline State University. No date. http://www.soil.ncsu.edu/publications/BMPs/buffers.html. Accessed October 2016. [↑](#footnote-ref-13)
13. Farrell P. 2014. The impact of climate change on soil erosion. *REACCH Annual Report*, Year 4. [↑](#footnote-ref-14)
14. Nearing, M, Pruski, F, and O’Neal, M. 2004. Expected climate change impacts on soil erosion rates: a review. *Journal of Soil and Water Conservation* **59(1)**: 43-50. [↑](#footnote-ref-15)
15. Soil and Water Conservation Society. 2003. *Conservation implications of climate change: Soil erosion and runoff from cropland*. [↑](#footnote-ref-16)
16. USDA. No date. http://blogs.usda.gov/2013/04/08/shade-grown-coffee-protects-puerto-rico-bay-coral-reefs/. Accessed October 2016. [↑](#footnote-ref-17)
17. USDA Natural Resources Conservation Service. 2012. “Managing Shade Coffee Fact Sheet.” [↑](#footnote-ref-18)
18. US Fish and Wildlife Service. No date. http://nctc.fws.gov/Pubs4/Shade_Coffee.pdf. Accessed October 2016. [↑](#footnote-ref-19)
19. Borkhataria R, Collazo J, Groom M and Jordan-Garcia. 2012. Shade-grown coffee in Puerto Rico: Opportunities to preserve biodiversity while reinvigorating a struggling agricultural commodity. *Agriculture, Ecosystems & Environment* **149**: 164-170. [↑](#footnote-ref-20)
20. Farrell P. 2014. The impact of climate change on soil erosion. *REACCH Annual Report*, Year 4. [↑](#footnote-ref-21)
21. Nearing, M, Pruski, F, and O’Neal, M. 2004. Expected climate change impacts on soil erosion rates: a review. *Journal of Soil and Water Conservation* **59(1)**: 43-50. [↑](#footnote-ref-22)
22. Soil and Water Conservation Society. 2003. *Conservation implications of climate change: Soil erosion and runoff from cropland*. [↑](#footnote-ref-23)
23. Nearing, M, Pruski, F, and O’Neal, M. 2004. Expected climate change impacts on soil erosion rates: a review. *Journal of Soil and Water Conservation* **59(1)**: 43-50. [↑](#footnote-ref-24)
24. Soil and Water Conservation Society. 2003. *Conservation implications of climate change: Soil erosion and runoff from cropland*. [↑](#footnote-ref-25)
25. Farrell P. 2014. The impact of climate change on soil erosion. *REACCH Annual Report*, Year 4. [↑](#footnote-ref-26)
26. Nearing, M, Pruski, F, and O’Neal, M. 2004. Expected climate change impacts on soil erosion rates: a review. *Journal of Soil and Water Conservation* **59(1)**: 43-50. [↑](#footnote-ref-27)
27. Soil and Water Conservation Society. 2003. *Conservation implications of climate change: Soil erosion and runoff from cropland*. [↑](#footnote-ref-28)
28. Greg L. Morris Engineering, Inc. 2014. *60% Design of Guánica Lagoon Restoration*. [↑](#footnote-ref-29)
29. Nearing, M, Pruski, F, and O’Neal, M. 2004. Expected climate change impacts on soil erosion rates: a review. *Journal of Soil and Water Conservation* **59(1)**: 43-50. [↑](#footnote-ref-30)
30. Soil and Water Conservation Society. 2003. *Conservation implications of climate change: Soil erosion and runoff from cropland*. [↑](#footnote-ref-31)
31. Protectores de Cuencas. 2016. Expanding Conservation/Sediment Control Practices in Priority Form Areas of the Guánica Bay Watershed [↑](#footnote-ref-32)
32. Ramos-Scharron, C and Thomaz, E. 2016. Runoff development and soil erosion in a wet tropical montane setting under coffee cultivation. *Land Degradation and Development*. DOI: 10.1002/ldr.2567 [↑](#footnote-ref-33)
33. Puerto Rico Climate Change Council (PRCCC). 2013. “Puerto Rico’s State of the Climate 2010-2013: Assessing Puerto Rico’s Social-Ecological Vulnerabilities in a Changing Climate.” Puerto Rico Coastal Zone Management Program, Department of Natural and Environmental Resources, NOAA Office of Ocean and Coastal Resource Management. San Juan, PR. [↑](#footnote-ref-34)
34. Greg L. Morris Engineering, Inc. 2016. *100% Design of Guánica Wastewater Treatment Wetland*. [↑](#footnote-ref-35)
35. Soil and Water Conservation Society. 2003. *Conservation implications of climate change: Soil erosion and runoff from cropland*. [↑](#footnote-ref-36)
36. Nearing, M, Pruski, F, and O’Neal, M. 2004. Expected climate change impacts on soil erosion rates: a review. *Journal of Soil and Water Conservation* **59(1)**: 43-50. [↑](#footnote-ref-37)
37. Whitall D, Bauer L, Sherman C, Edwards K, Mason A, Pait T and Caldow C. 2013. *Baseline Assessment of Guánica Bay, Puerto Rico in Support of Watershed Restoration*. NOAA Technical Memorandum NOS NCCOS 176. [↑](#footnote-ref-38)
38. Fourqurean J, Powell G, Kenworthy W, Zieman J. 1995. The effects of long-term manipulation of nutrient supply on competition between the seagrasses *Thalassia testudinum* and *Halodule wrightii* in Florida Bay. *OIKOS* **72**: 349-358. [↑](#footnote-ref-39)
39. Munday P, Dixson D, Donelson J, Jones G, Pratchett M, Devitsina G and Døving K. 2009. Ocean acidification impairs olfactory discrimination and homing ability of a marine fish. *Proceedings of the National Academy of Science*s **106(6)**: 1848–1852. [↑](#footnote-ref-40)
40. Devine B and Munday P. 2013. Habitat preferences of coral-associated fishes are altered by short-term exposure to elevated CO_2_. *Marine Biology* **160**: 1955. doi:10.1007/s00227-012-2051-1. [↑](#footnote-ref-41)
41. Munday P, Cheal A, Dixon D, Rummer J and Fabricius K. 2014. Behavioural impairment in reef fishes caused by ocean acidification at CO_2_ seeps. *Nature Climate Change* **4**: 487-492. [↑](#footnote-ref-42)
42. Soil and Water Conservation Society. 2003. *Conservation implications of climate change: Soil erosion and runoff from cropland*. [↑](#footnote-ref-43)
43. Nearing, M, Pruski, F, and O’Neal, M. 2004. Expected climate change impacts on soil erosion rates: a review. *Journal of Soil and Water Conservation* **59(1)**: 43-50. [↑](#footnote-ref-44)
44. Whitall D, Bauer L, Sherman C, Edwards K, Mason A, Pait T and Caldow C. 2013. *Baseline Assessment of Guánica Bay, Puerto Rico in Support of Watershed Restoration*. NOAA Technical Memorandum NOS NCCOS 176. [↑](#footnote-ref-45)
45. Gilman E, Ellison J, Duke N, Field C. 2008. Threats to mangroves from climate change and adaptation options. Aquatic Botany **89(2)**: 237-250. [↑](#footnote-ref-46)
46. Alongi D. 2015. The impact of climate change on mangrove forests. *Current Climate Change Reports* **1**:30. [↑](#footnote-ref-47)
47. Munday P, Dixson D, Donelson J, Jones G, Pratchett M, Devitsina G and Døving K. 2009. Ocean acidification impairs olfactory discrimination and homing ability of a marine fish. *Proceedings of the National Academy of Science*s **106(6)**: 1848–1852. [↑](#footnote-ref-48)
48. Munday P, Cheal A, Dixon D, Rummer J and Fabricius K. 2014. Behavioural impairment in reef fishes caused by ocean acidification at CO_2_ seeps. *Nature Climate Change* **4**: 487-492. [↑](#footnote-ref-49)
49. Ecocean. No date. <http://www.ecocean.fr/en/> Accessed August 2016. [↑](#footnote-ref-50)
50. Daw T, Adger W, Brown K, and Badjeck M-C. 2009. Climate change and capture fisheries: potential impacts, adaptation and mitigation. In K. Cochrane, C. De Young, D. Soto and T. Bahri (eds). *Climate change implications for fisheries and aquaculture: overview of current scientific knowledge*. FAO Fisheries and Aquaculture Technical Paper. No. 530. Rome, FAO. pp. 107-150. [↑](#footnote-ref-51)
51. Munday P, Dixson D, Donelson J, Jones G, Pratchett M, Devitsina G and Døving K. 2009. Ocean acidification impairs olfactory discrimination and homing ability of a marine fish. *Proceedings of the National Academy of Science*s **106(6)**: 1848–1852. [↑](#footnote-ref-52)
52. Pankhurst N and Munday P. 2011. Effects of climate change on fish reproduction and early life history stages. *Marine and Freshwater Research* **62(9)**: 1015-1026. [↑](#footnote-ref-53)
53. Dixson D, Munday P, Pratchett M, and Jones G. 2011. Ontogenetic changes in response to settlement cues by anemonefish. *Coral Reefs* **30**: 903-910. [↑](#footnote-ref-54)
54. Munday P, Dixson D, Donelson J, Jones G, Pratchett M, Devitsina G and Døving K. 2009. Ocean acidification impairs olfactory discrimination and homing ability of a marine fish. *Proceedings of the National Academy of Science*s **106(6)**: 1848–1852. [↑](#footnote-ref-55)
55. Miller G, Waston SA, McCormick M and Munday P. 2013. Increased CO_2_ stimulates reproduction in a coral reef fish. *Global Change* Biology doi: 10.1111/gcb.12259. [↑](#footnote-ref-56)
56. Munday P, Cheal A, Dixon D, Rummer J and Fabricius K. 2014. Behavioural impairment in reef fishes caused by ocean acidification at CO_2_ seeps. *Nature Climate Change* **4**: 487-492. [↑](#footnote-ref-57)
57. Soil and Water Conservation Society. 2003. *Conservation implications of climate change: Soil erosion and runoff from cropland*. [↑](#footnote-ref-58)
58. Nearing, M, Pruski, F, and O’Neal, M. 2004. Expected climate change impacts on soil erosion rates: a review. *Journal of Soil and Water Conservation* **59(1)**: 43-50. [↑](#footnote-ref-59)
59. Whitall D, Bauer L, Sherman C, Edwards K, Mason A, Pait T and Caldow C. 2013. *Baseline Assessment of Guánica Bay, Puerto Rico in Support of Watershed Restoration*. NOAA Technical Memorandum NOS NCCOS 176. [↑](#footnote-ref-60)
60. Doropoulos C, Ward S, Diaz-Pulido G, Hoegh-Gulberg O, Mumby P. 2011. Ocean acidification reduces coral recruitment by disrupting intimate larval-algal settlement interactions. *Ecology Letters* **15**: 338-346. [↑](#footnote-ref-61)
61. Webster N, Uthicke S, Botte E, Flores F and Negri A. 2013. Ocean acidification reduces induction of coral settlement by crustose coralline algae. *Global Change Biology* **19(1)**: 303-315. [↑](#footnote-ref-62)
